# Three-component systems represent a common pathway for extracytoplasmic addition of pentofuranose sugars into bacterial glycans

**DOI:** 10.1073/pnas.2402554121

**Published:** 2024-05-15

**Authors:** Steven D. Kelly, Nam Ha Duong, Jeremy T. Nothof, Todd L. Lowary, Chris Whitfield

**Affiliations:** ^a^Department of Molecular and Cellular Biology, University of Guelph, Guelph, ON N1G 2W1, Canada; ^b^Institute of Biological Chemistry, Academia Sinica, Nangang, Taipei 11529, Taiwan; ^c^Chemical Biology and Molecular Biophysics, Taiwan International Graduate Program, Academia Sinica, Nangang, Taipei 11529, Taiwan; ^d^Department of Chemistry, National Tsing Hua University, Hsinchu 300044, Taiwan; ^e^Department of Chemistry, University of Alberta, Edmonton, AB T6G 2G2, Canada; ^f^Institute of Biochemical Sciences, National Taiwan University, Taipei 10617, Taiwan

**Keywords:** microbial glycobiology, polysaccharides, glycosylation, glycosyltransferases, pentofuranoses

## Abstract

Cell surface glycans are major drivers of antigenic diversity in bacteria. The biochemistry and molecular biology underpinning their synthesis are important in understanding host–pathogen interactions and for vaccine development with emerging chemoenzymatic and glycoengineering approaches. Structural diversity in glycostructures arises from the action of glycosyltransferases (GTs) that use an immense catalog of activated sugar donors to build the repeating unit and modifying enzymes that add further heterogeneity. Classical Leloir GTs incorporate α- or β-linked sugars by inverting or retaining mechanisms, depending on the nucleotide sugar donor. In contrast, the mechanism of known ribofuranosyltransferases is confined to β-linkages, so the existence of α-linked ribofuranose in some glycans dictates an alternative strategy. Here, we use *Citrobacter youngae* O1 and O2 lipopolysaccharide O antigens as prototypes to describe a widespread, versatile pathway for incorporating side-chain α-linked pentofuranoses by extracytoplasmic postpolymerization glycosylation. The pathway requires a polyprenyl phosphoribose synthase to generate a lipid-linked donor, a MATE-family flippase to transport the donor to the periplasm, and a GT-C type GT (founding the GT136 family) that performs the final glycosylation reaction. The characterized system shares similarities, but also fundamental differences, with both cell wall arabinan biosynthesis in mycobacteria, and periplasmic glucosylation of O antigens first discovered in *Salmonella* and *Shigella*. The participation of auxiliary epimerases allows the diversification of incorporated pentofuranoses. The results offer insight into a broad concept in microbial glycobiology and provide prototype systems and bioinformatic guides that facilitate discovery of further examples from diverse species, some in currently unknown glycans.

## Significance

Pathogenic bacteria produce diverse polysaccharides that are important for virulence and can be exploited in the development of vaccines and immunotherapies. Resolving the mechanisms of polysaccharide biosynthesis is vital for understanding the molecular basis of antigenic diversity and for exploiting the pathways in glycoengineering applications. Here, we elucidate the enzymatic origin of α-ribofuranose residues in a prototypical system from gram-negative lipopolysaccharide O antigens and reveal unanticipated relationships to processes used in the assembly of mycobacterial cell walls. The periplasmic glycosylation pathway can introduce different pentofuranoses through the action of different epimerases. The identification of this pathway also expands the toolbox of enzymes that may be deployed in glycoengineering and recombinant production of polysaccharides with desired structures and properties.

The bacterial cell surface displays structurally diverse glycoconjugates with crucial roles in cell viability, host–pathogen and symbiont interactions, and protection from extracellular environmental factors. The immense diversity in bacterial glycans arises from a large collection of component sugars, different linkage configurations, and noncarbohydrate substituents. The majority of sugars in glycans across all forms of life are assembled into natural products by Leloir glycosyltransferase (GT) enzymes, which use cytoplasmic nucleotide mono- and diphosphosugars as the activated donors and proteins with similar folds to catalyze formation of either α- or β-glycosidic linkages (1). We recently described a notable exception—ribofuranose (Rib*f*)—where a prototypical family of cytoplasmic enzymes use phosphoribosylpyrophosphate (PRPP) as a donor for incorporation of Rib*f* into polysaccharides (2). The identified Rib*f* GTs are substantially different from conventional Leloir GTs because they perform a two-step reaction. They are composed of a glycan phosphoribosyltransferase (gPRT) domain to transfer β-linked Rib*f*-5-P to an acceptor sugar and a haloacid dehalogenase (HAD)-family phosphoribose phosphatase (PRP) domain to dephosphorylate the gPRT product. Using the PRP domain as a bioinformatic marker for Rib*f-*adding enzymes, we identified family members spanning bacterial genera involved in synthesis of different types of complex carbohydrate end products. In all cases, the bipartite Rib*f*-GTs transfer β-linked Rib*f* residues. Bioinformatic screens also identified some sequence-based outliers compared to the prototypical enzymes. One group is involved in the synthesis of lipopolysaccharide (LPS) in *Citrobacter* and *Stenotrophomonas*. The genetic loci responsible for synthesis of these OPSs lack genes for a prototypical gPRT, and the products contain α-linked Rib*f*, suggesting a fundamentally different mechanism for Rib*f* incorporation. The objective of this study was to elucidate this mechanism.

LPS is a major component of the outer membrane in almost all gram-negative bacteria. This important glycolipid contains the well-conserved lipid A, providing the lipid component of the outer leaflet of the outer membrane. Lipid A is linked to a core oligosaccharide that shows some variation within and between species. The core is capped with a structurally hypervariable O-antigen polysaccharide (OPS) providing the basis for O-antigen serotyping (3). OPS biosynthesis is almost always performed using one of two major strategies, known as the Wzx/Wzy-dependent pathway and the ABC transporter–dependent pathway (reviewed in ref. 4). In the most common Wzx/Wzy-dependent pathway, single repeating units are built on a membrane-embedded undecaprenyl-pyrophosphate (undPP) carrier by the combined activities of an initiating phosphoglycosyltransferase (PGT) and specific GTs. UndPP-linked repeat units are then flipped to the periplasm by the Wzx flippase. A membrane-embedded Wzy polymerase then elongates the OPS by an *en bloc* process using individual undPP-linked repeat units as glycosyl donors. In contrast, the biosynthesis of some OPSs follows a fundamentally different pathway, where the glycan backbone is fully polymerized inside the cell prior to export by an ATP-binding cassette (ABC) transporter. Broadly applicable prototypes for this assembly strategy have been described in *Klebsiella pneumoniae* O2a and in *Escherichia coli* O9a (4). In these systems, a PGT and structure-specific GTs perform sequential reactions in the cytoplasm to create a full-length undPP-linked polysaccharide, which is a substrate for the ABC transporter. The mature OPS produced by either of the two assembly pathways is ligated to the lipid A-core oligosaccharide (lipid A-core) by the OPS ligase enzyme (WaaL) and the completed LPS is transported to the outer leaflet of the outer membrane by conserved LPS transport (Lpt) machinery.

**Fig. 1.**
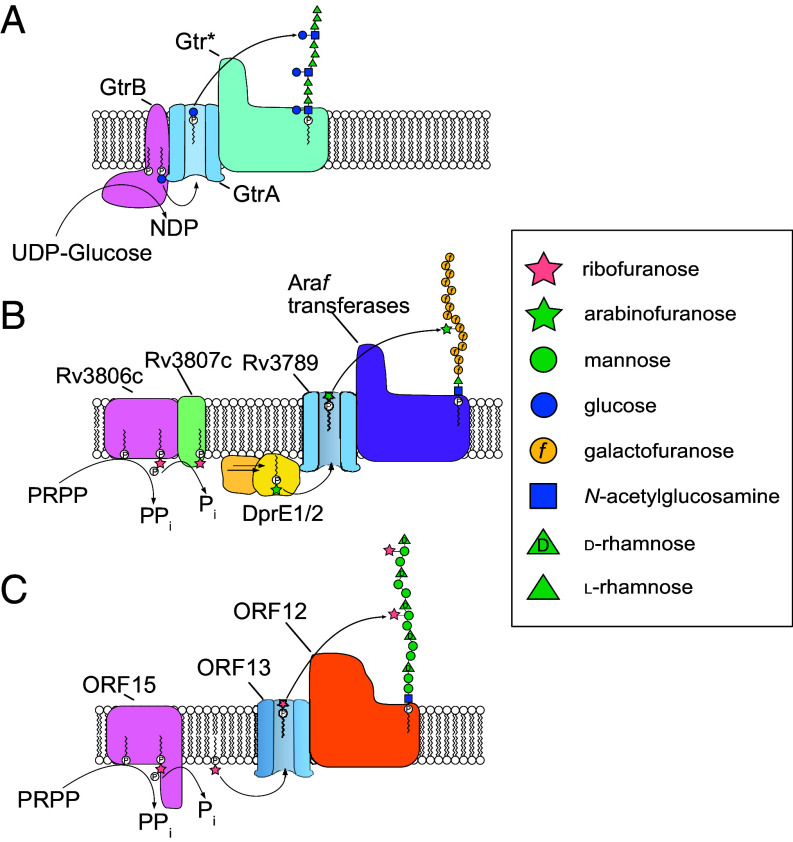
Models for extracytoplasmic glycosylation prototypes. (*A*) Some *Shigella flexneri* O-serotypes glucosylates a serotype Y O-polysaccharide backbone at specific sites to create new O serotypes. The immediate donor for periplasmic glycosylation is undecaprenyl-phosphate-linked glucose (undP-Glc) (8), distinguishing the donor from the undPP-linked repeating units. GtrB is a undP-Glc synthase (9), whose activity is analogous to eukaryotic dolichol-phosphomannose synthase participating in *N*-linked protein glycosylation (5). GtrA is a proposed flippase that exports undP-Glc to the periplasm. A serotype-specific Gtr* (GT-C) enzyme completes the glycosylation process. (*B*) Mycobacterial cell wall arabinosylation uses phosphoribosylpyrophosphate (PRPP) as the initial donor and begins with the transfer of ribose-5-phosphate from PRPP to decP catalyzed by the Rv3806c DPPRS. Rv3807c dephosphorylates the 5′ position of DPPR to make decP-Rib*f* (DPR), which is then epimerized by DprE1/E2 epimerize to form decP-Ara*f* (DPA). Epimerization is shown in the cytoplasm, although there is some uncertainty about the location of this step (see the text). Rc3789 is a small multidrug-resistant (SMR) family exporter that is proposed to flip DPA to the periplasm, where several different GT-C Ara*f* transferases glycosylate a galactan. (*C*) *C. youngae* O1 OPS possesses Rib*f* side chain residues introduced using processes elucidated in this study. The ORF15^C^ PPPRS synthesizes undP-Rib-5-P from PRPP and the PRP module (ORF15^N^) removes the 5′-phosphate. The ORF13 MATE transporter flips undP-Rib*f* to the periplasm where the ORF12 GT-C uses it as an activated donor to glycosylate the polymerized OPS backbone. Sugars are represented using the symbol nomenclature for glycans (SNFG) (10).

While OPS biosynthesis is accomplished predominantly via the primary pathways, some bacteria employ additional postpolymerization sugar additions to undPP-linked OPS intermediates in the periplasm, prior to ligation to lipid A-core. The classical examples involve glucosylation of OPS in *Salmonella*, *Shigella,* and *Escherichia*, which build their OPS by Wzx/Wzy-dependent mechanisms and use a three-component system for periplasmic glycosylation. The hallmark features of the glycosylation processes are a GT-C-fold GT and a lipid-linked donor substrate (reviewed in ref. 5) (Fig. 1*A*). Such modifications result in O-antigen seroconversion; for example, in *Shigella*, glucosylation of a shared backbone repeating unit (serotype Y) in different linkages/positions creates distinct serotypes (6). More recently, a similar three-component seroconversion pathway involving galactose-addition has been reported in *Klebsiella*, where the OPSs are synthesized in ABC transporter-dependent processes (7).

The study of bacterial glycan structures is vitally important in the era of widespread antibiotic resistance because these glycans are important candidates for vaccine design or the production of protective antibodies against clinically important pathogens (11). Defining the pathways for glycan biosynthesis is essential to enable contemporary approaches for in vivo engineering of protein–polysaccharide bioconjugate vaccines (12). Here, we describe a three-component system that catalyzes postpolymerization (periplasmic) glycosylation with α-Rib*f* and is flexibly diversified to add xylofuranose (α-Xyl*f*) and, by extension, other pentofuranoses. The system combines features seen in periplasmic OPS hexose modification in gram-negative bacteria, and extracytoplasmic cell wall arabinan biosynthesis, which is essential in mycobacteria. These systems reveal an elegant and shared solution for the synthesis and diversification of important bacterial glycans.

## Results

### Bioinformatic Analysis of the OPS Biosynthesis Loci Predicts Closely Related Assembly Pathways in *Citrobacter youngae* O1 and O2.

**Fig. 2.**
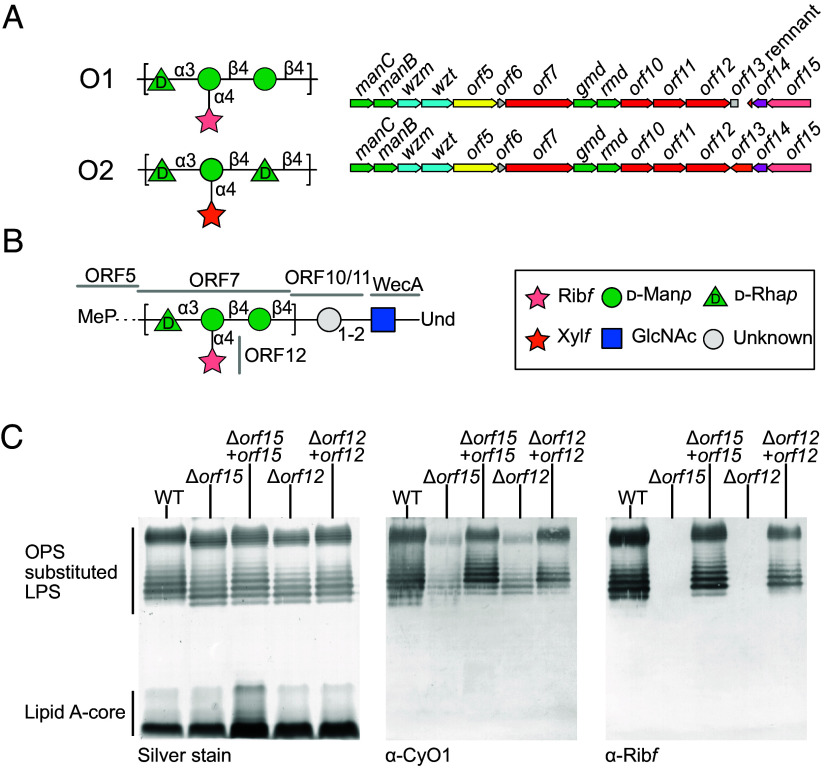
*C. youngae* O1 and O2 OPS repeat unit structures, corresponding OPS-biosynthesis genetic loci and LPS phenotypes of *C. youngae* O1 Δ*orf1*2 and Δ*orf15* mutants. (*A*) Genes are colored according to predicted functions: activated sugar donor synthesis (green), ABC transporter (cyan), terminator (yellow), glycosyltransferase (red), hypothetical/truncated (gray), MATE family transporter (pink), epimerase (orange), PRP encoding gene (salmon). (*B*) Based on similarities of the encoded proteins to characterized prototypes, predicted functions for the ORFs can be made in serotype O1. WecA is the PGT enzyme initiating biosynthesis by the transfer of GlcNAc-1-P to undP. ORF10 and ORF11 share similarity with adapter synthesizing mannosyltransferases from *E. coli* O9a, but the reducing end sugars in O1 are unknown. ORF7 and ORF5 polymerize and terminate the main chain repeat unit, respectively, and ORF12 modifies the backbone with Rib*f*. (*C*) Whole-cell lysates from the mutants and from mutants carrying a plasmid-encoded complementing gene, were separated by SDS-PAGE and detected by silver staining. The same samples were also probed by western immunoblotting using antiserum against the cells containing the O1 antigen, and with anti-Rib*f* side-chain antiserum obtained by adsorbing the O1 antiserum with cells of the Δ*orf15* mutant (shown to lack the Rib*f* side-chain in this study).

*C. youngae* O1 and O2 antigens were highlighted for investigation because the genetic loci for their production contain a protein with a PRP domain. *C. youngae* serotype O1 OPS has a repeating unit backbone composed of [→4)-α-ᴅ-Rha*p*-(1→3)-β-ᴅ-Man*p*^I^-(1→4)-β-ᴅ-Man*p*^II^-(1→], in which ~50% of Man*p*^I^ is modified at C4 with an α-ᴅ-Rib*f* residue (13) (Fig. 2*A*). Serotype O2 possesses a similar backbone to O1, differing by replacing the ᴅ-Man*p*^II^ residue with ᴅ-Rha*p*, but the two repeating-unit structures are further distinguished by the identity of a side-chain pentofuranose residue on Man*p*^I^; in serotype O2, Rib*f* is replaced by its C-3 epimer, xylofuranose (Xyl*f*) (14) (Fig. 2*A*). Not surprisingly, the corresponding OPS biosynthesis genetic loci share most of the same genes (Fig. 2*A*), and the sequences of the corresponding gene products are almost identical (96% identity across the entire cluster). Activities of several gene products can be confidently assigned by sequences shared with well-characterized proteins (*SI Appendix*, Table S1). These include Wzm and Wzt proteins, the characteristic transmembrane, and nucleotide-binding domains of the pathway-defining ABC transporter (15). Four gene products are involved in the generation of activated donors for the Man and Rha residues. ManB (phosphomannomutase) and ManC (mannose-1-phosphate guanylyltransferase) act sequentially to convert Man-6-P to GDP-Man (16). Gmd (GDP-Man-4,6-dehydratase) and Rmd (GDP-6-deoxy-D-*lyxo*-hexos-4-ulose-4-reductase) convert GDP-Man to GDP-Rha (17, 18).

The initiating PGT enzyme for OPS synthesized in ABC transporter-dependent pathways is frequently provided by an undecaprenyl-phosphate: α-N-acetylglucosaminyl-1-phosphate transferase, known as WecA, encoded by a gene located elsewhere on the chromosome (19). The WecA product (undPP-GlcNAc) is then extended by one or more “adapter” GTs that transition the conserved reducing terminal GlcNAc to an acceptor appropriate for the serotype-specific (repeating unit) GTs that sequentially polymerize the glycan chain. In these systems, the polymerizing GT modules are frequently located in multimodule proteins (4). Sequence data for the *C. youngae* OPS loci predict five conventional Leloir GTs modules, whose proposed roles are shown in Fig. 2*B*. ORF7 is a large protein with three putative GT modules (a central GT1-family enzyme flanked by two GT4s) and is hypothesized to be the polymerase, generating the Rha*p*-Man*p*-Man*p*/Rha*p* trisaccharide repeating unit backbone shared by serotypes O1 and O2. In this scenario, ORF10 and ORF11 (which encode monofunctional GTs belonging to the GT4 family) would synthesize the adaptor. Consistent with this, ORF10 and ORF11 share similarity with two characterized mannosyltransferases (WbdB and WbdC), which create the adaptor in several OPSs, including the influential model system from *E. coli* O9a (20). Unfortunately, the reported *C. youngae* OPS structures focus on the repeating-unit domain, and attempts to retroactively identify the reducing terminus and the adaptor regions of the *C. youngae* O1 and O2 OPS from the published data were unsuccessful, due to low signal intensity compared to the repeating unit.

A subset of OPSs assembled by ABC transporter-dependent pathways contain nonreducing chain terminating residues; these can also be difficult to identify in complex NMR spectra and can be overlooked. During biosynthesis, a chain-terminating enzyme possessing a hallmark coiled-coil molecular ruler acts to block further elongation once a particular glycan size is achieved (4). ORF5 contains a C-terminal coiled-coil predicted by DeepCoil (21) and shares 56% similarity with the bifunctional methyltransferase-kinase chain-terminator prototype [WbdD; (22)] from *E. coli* O9a (*SI Appendix*, Table S1). In support of this assignment, NMR spectra collected in this study (see below) are consistent with a terminal methyl-phosphate moiety. Chain-terminating enzymes are typically accompanied by an ABC transporter whose NBD possesses a C-terminal carbohydrate-binding module (CBM), which recognizes the terminal modification to prevent premature export of unmodified glycan (4). The *C. youngae* O1 and O2 Wzt proteins share 76% similarity with the *E. coli* O9a Wzt protein, extending into a predicted CBM (*SI Appendix*, Table S1). This and the chain termination strongly suggest a similar coupling of termination and export in these bacteria.

Together, the predicted activities of ORFs 1 to 11 are sufficient for the initiation, polymerization, and termination/export of the main chain rhamnose/mannose backbone (Fig. 2*B*). However, the loci possess three additional downstream genes in O1 and four in O2 (*orf12* to *orf15*) (Fig. 2*A*) that have no assigned function in glycan backbone synthesis. One of these, ORF15, contains the putative PRP domain identified in a previous bioinformatics screen (2). Further bioinformatic and biochemical analysis presented below indicates that these proteins form a three-component system responsible for periplasmic postpolymerization addition of pentofuranose residues in *C. youngae* O1 and O2. These pathways require i) an enzyme (ORF15) to charge polyprenyl phosphate (presumably undP in *Citrobacter*) with the modifying pentofuranose; ii) a flippase (ORF14) to export this lipid glycosylation donor across the cytoplasmic membrane; and iii) a GT-C family enzyme (ORF12) to add the pentofuranose residue to the repeating unit in the periplasm. Data confirming these assignments (and the pathway shown in Fig. 1*C*) are presented below.

### Mutant Phenotypes Confirm Postpolymerization Modification of *C. youngae* O1 OPS.

To definitively establish the involvement of the putative three-component modification system in OPS biosynthesis in *C. youngae* O1 (and by extension O2), single gene mutants were constructed by replacing the chromosomal *orf12* and *orf15* genes with kanamycin-resistance cassettes. These mutations were designed to be nonpolar, and the transcription of downstream genes was confirmed by restoration of wild-type LPS phenotypes by single gene complementation. SDS-PAGE of whole cell lysates of wild-type and mutants showed a ladder pattern typical of OPS-substituted LPS molecules but both mutations resulted in a slight downward shift in the average chain lengths in the clusters of high molecular weight LPS bands (Fig. 2*C*). When the LPS species were probed by western immunoblotting with antibodies raised against serotype O1, immunoreactivity was substantially reduced in the mutants, suggesting loss of a major epitope in their OPS-substituted LPS. In each case, the wild-type SDS-PAGE profile and antibody reactivity were restored by transformation of the mutants with a plasmid containing the corresponding deleted gene.

The structures of OPSs from the Δ*orf15* and Δ*orf12* mutants and the corresponding complemented strains were then determined by NMR spectroscopy (*SI Appendix*, Table S2 and Fig. S1 *A* and *B*). Chemical shift data from one-dimensional ^1^H and ^13^C, and two-dimensional ^1^H, ^13^C HSQC experiments for OPS from the mutants were identical to the published data for chemically deribosylated O1 OPS, containing a trisaccharide repeat unit (13) (*SI Appendix*, Table S2). Cells from the Δ*orf15* mutant were used to adsorb the O1 antiserum, removing antibodies reacting against the backbone epitopes. Adsorbed Rib*f*-specific antiserum retained reactivity with the wild-type LPS but lost all residual activity against the mutants, reinforcing the structural and bioinformatic-based data showing that *orf12* and *orf15* (and by inference *orf14*) are involved in generation of a side-chain epitope distinct from the glycan backbone. Consistent with the PAGE and western immunoblot results above, the NMR data for OPS from the complemented mutants were identical to authentic serotype O1 OPS. The presence in the NMR signals and PAGE profiles of some wild-type OPS molecules lacking Rib*f* is typical of nonstoichiometric postpolymerization modification reactions (7).

### ORF15 Is a Polyprenyl Phosphoribofuranose Synthase (PPPRS).

**Fig. 3.**
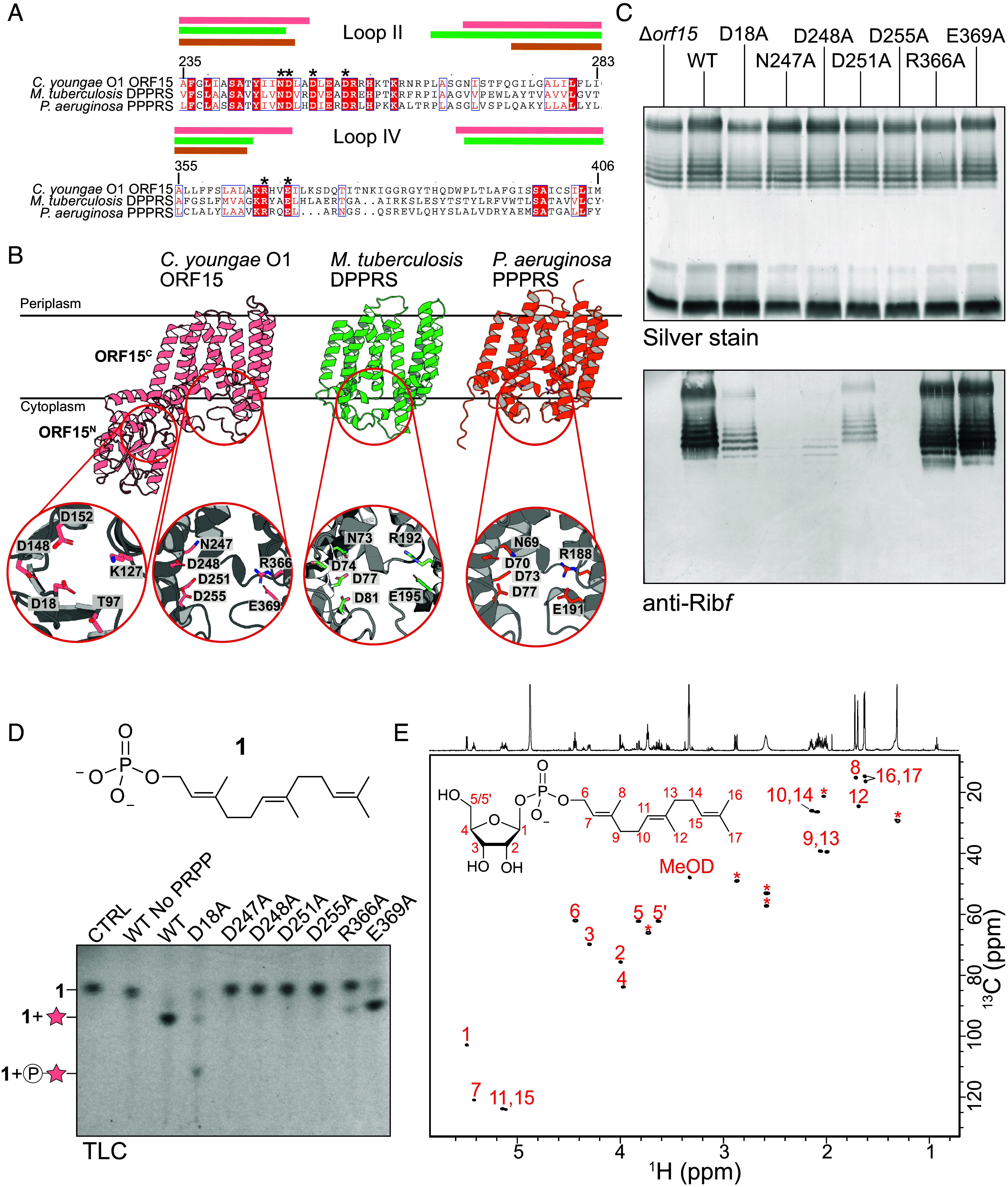
Predicted structure and activity of ORF15, a polyprenylphosphoryl-5′-phosphoribosyltransferase enzyme. (*A*) Multiple sequence alignment of conserved regions of polyprenylphosphoryl-5′-phosphoribosyltransferases from *M. tuberculosis* Rv3806c (P9WFR5), *P. aeruginosa* PSPA7_6246 (WP_003152550.1), and *C. youngae* O1 ORF15 (AWU66546.1). The colored bars (color coded to match the AlphaFold models) above the alignment indicate transmembrane helices predicted by TMHMM. The residue numbers correspond to *C. youngae* ORF15, and the asterisks indicate residues targeted for alanine replacement. (*B*) AlphaFold models for ORF15, and *P. aeruginosa* PPPRS compared to the solved structure of *M. tuberculosis* DPPRS [8j8j, (23)]. The mycobacterial enzyme forms a trimer in the membrane, but only one protomer is shown here for clarity. ORF15 is distinguished by possession of a phosphoribosyl-phosphatase (PRP) domain. The active sites for the PRP and PPPRS domains are expanded (the *M. tuberculosis* enzyme is named DPPPRS because the lipid substrate is known to be a decP). (*C*) Structure-guided alanine-replacement mutants in the PRP (D18A) and PPPRS (D247A, D248A, D251A, R366A, and E369A) modules were tested for in vivo activity by genetic complementation of the *C. youngae* O1 Δ*orf15* mutant. Whole-cell lysates were examined by silver-stained SDS-PAGE and western immunoblotting with antiserum specific for the Rib*f* side-chain. (*D*) The same variants were tested in in vitro reactions using membranes as a source of enzyme, farnesylP (**1**; shown) acceptor and PRPP donor. The reaction mixtures were separated by TLC (*Left*) (*E*) ^1^H,^13^C HSQC of the scaled-up in vitro reaction product produced by wild-type ORF15. The product was confirmed to the farnesylP-Rib*f* (chemical structure shown on the spectrum) and the critical NMR peaks are labeled. Peaks marked with an asterisk were due to an undetermined contaminant, but these did not hinder the interpretation of the product.

The N-terminal domain of ORF15 (ORF15^N^) was identified as a putative PRP in our earlier bioinformatic survey (2), suggesting a role in Rib*f* incorporation. AlphaFold modeling of ORF15^N^ predicts a soluble domain containing the expected central Rossmann domain and an α-helical “cap,” together with the conserved active site loops typical of structures of other proteins in the HAD superfamily, including all previously identified PRP domains (Fig. 3*B*) (2).

The remaining part of the protein (ORF15^C^) shares no similarity with known gPRT proteins, so the potential role of ORF15^N^ in Rib*f*-5-P GT activity was investigated. BLAST comparisons suggested ORF15^C^ is an integral membrane protein belonging to the UbiA family of prenyltransferases, which perform diverse prenylation reactions at the membrane interface (24). Although many of the BLAST “hits” were uncharacterized proteins, the closest characterized homolog (31% sequence identity, 53% similarity) was found to be *Mycobacterium tuberculosis* decaprenylphosphoryl-5′-phosphoribosyltransferase (DPPRS). DPPRS is part of the pathway that generates cell wall–associated arabinogalactan and lipoarabinomannan in the extracellular space of mycobacteria (23, 25, 26) (Fig. 1*B*). DPPRS uses PRPP as the donor to transfer Rib*f*-5-P to decaprenyl-phosphate (decP) in the cytoplasm as the first committed step for arabinose transfer (27). Note that mycobacteria employ decaprenol for glycan and peptidoglycan biosynthesis, instead of the undecaprenol typical in gram-negative bacteria (28). In *M. tuberculosis*, *Rv3807c* encodes a phosphatase (unrelated to HAD-family PRPs) that removes the 5′-phosphate, producing decP-Rib*f* (DPR) (26). DPR is then epimerized by DprE1 (oxidase) and DprE2 (reductase) to make decP-arabinofuranose (DPA). The final steps in the mycobacterial pathways resemble the periplasmic glycosylation of OPS but differ in using a decaprenyl-linked Ara*f* donor. A DPPRS ortholog is also proposed to synthesize undP-Ara*f* as the donor for type IV pilus *O*-glycosylation in *Pseudomonas aeruginosa* (29). The *P. aeruginosa* enzyme is designated as PPPRS (with the initial “P” referring to “polyprenyl”) because the lipid acceptor has not been experimentally established, although it is expected to be undP because typical gram-negative bacteria do not synthesize decP. *P. aeruginosa* PPPRS shares 34% identity/53% similarity with *C. youngae* ORF15^C^. AlphaFold models of the three proteins show a similar integral membrane protein component, with folds resembling archetypal UbiA family members, composed of nine transmembrane helices and a central cavity housing two aspartate-rich regions for Mg^2+^-mediated coordination of phospholipid substrates (Fig. 3 *A* and *B*) (24).

The PPPRS activity of ORF15^C^ was definitively established by biochemical experiments. Farnesyl-phosphate (farnesylP; **1**) (30) was used as an acceptor instead of undP because its improved solubility resulted in relatively easier handling. ORF15 is an integral membrane protein, and we were only able to purify small amounts of protein that was no longer active protein for in vitro assays, so membranes prepared from cells expressing plasmid-encoded ORF15 were used as the source of active enzyme. The products of reactions containing membranes, PRPP donor, and farnesylP acceptor were analyzed by thin layer chromatography (TLC), revealing production of a slower migrating species that was dependent on the presence of PRPP (Fig. 3*D*). The mass spectrum of the reaction mixture showed a major species with *m/z* 435, expected for the addition of ribose (exact mass 132) to farnesylP (exact mass 303) (*SI Appendix*, Fig. S3). This reaction was scaled up and the purified product was analyzed by ^1^H,^13^C HSQC, ^1^H,^1^H COSY, and ^1^H,^1^H TOCSY NMR experiments, which confirmed the identity of the product (Fig. 3*E*).

Given the similarity shared by ORF15^C^ and mycobacterial DPPRS, we investigated the importance of conserved residues identified by multiple sequence alignment with *M. tuberculosis* DPPRS and *P. aeruginosa* PPPRS and AlphaFold modeling (Fig. 3 *A* and *B*). Conserved motifs were identified in cytoplasmic loops II (including ORF15^C^ residues N247, D248, D251, and D255) and IV (R366 and E369) (Fig. 3*A*). The equivalent residues have been shown to be required for activity of *M. tuberculosis* DPPRS in vitro (31). Of these mutants, only R366A and E369A retained any detectable ORF15^C^ activity in vitro (Fig. 3*D*). The in vivo activities of the mutant proteins were also assessed by their ability to restore Rib*f* side-chain addition (and reactivity with the Rib*f*-specific antiserum) in the Δ*orf15* mutant (Fig. 3*C*). Immunoreactive LPS profiles indistinguishable from WT were observed in transformants expressing ORF15^R366A^ and ORF15^E369A^. In contrast, ORF15^D251A^ showed severely reduced reactivity, while signals for ORF15^D248A^, ORF15^N247A^, and ORF15^D255A^ were barely detected under normal exposure. These results are consistent with the in vitro experiments, except for ORF15^D251A^, which retained some activity in vivo but none in vitro. This difference likely reflects the different sensitivities of the approaches. As this work was in the final stages of revision, a structure of the mycobacterial PPRS was published (23). The substrate-bound DPPRS reveals the active site and adds a structural explanation to the results for ORF15^C^. The two structures are very similar (Z-score 25.9, RMSD of 2.7 Å) and, as expected, conserved residues of the active sites are positioned identically (*SI Appendix*, Fig. S2). ORF15^N247^ is critical as it coordinates a Mg^++^ ion and interacts with Rib*f* in the donor substrate, while ORF15^D255^ only interacts with the Mg^++^. ORF15^R366^ and ORF15^E369^ are located in the active site but do not mediate substrate interactions, explaining the retained activity. ORF15^D248^ and ORF15^D255^ do not interact with substrate but presumably contribute to active site structure.

The distinguishing feature of ORF15^C^ is the PRP domain. Activity of PRP enzymes requires a conserved aspartic acid in loop I of the active site as a catalytic base to act as a nucleophile to dephosphorylate transferred Rib*f*-5′-phosphate (2). In ORF15^N^, this residue is D18 (Fig. 3*B*). In vitro reactions containing ORF15^D18A^ produced a slower migrating species (Fig. 3*D*), and mass spectrometry of the reaction products revealed a species with *m/z* 513, as expected for the addition of Rib*f*-5-P (exact mass 210) to FP acceptor (exact mass 303) (*SI Appendix*, Fig. S3*A*). Notably, the D18A mutant did not fully inactivate ORF15^C^; a small amount of farnesylP-Rib*f* was also detected both by TLC and mass spectrometry (Fig. 3*D* and *SI Appendix*, Fig. S3*A*), and in the western immunoblot of whole cell lysates Rib*f* side chains were detected (Fig. 3*C*). The molecular basis for this difference from the canonical PRP is unclear but did not compromise the conclusion that ORF15 participates in Rib*f* incorporation into the predicted lipid carrier of undP.

### ORF14 Is a Predicted MATE Transporter.

BLAST searches predict ORF14 is a MATE family protein. MATE family transporters export a wide range of compounds through an antiport mechanism with a coupling cation like H^+^ or Na^+^ (32). ORF14 shares 52% similarity (62% sequence coverage) with the functionally characterized EmrE protein, which effluxes several different toxic compounds from the cytoplasm. Notably, ORF14 also shares 48% similarity (46% sequence coverage) with ArnE, a MATE family transporter responsible for flipping undP-linked 4-aminoarabinose (Ara4N). This compound is the donor for a well-studied lipid A modification that provides resistance to polymyxins (33). The AlphaFold model of ORF14 shows four transmembrane helices and is consistent with structures of other MATE transporters, (*SI Appendix*, Fig. S4). Furthermore, modeling ORF14 as a dimer predicted an antiparallel orientation of the protomers, a feature seen in other MATE transporters (32). While not experimentally verified, the data are entirely consistent with ORF14 providing the required transporter for undP-Rib*f*.

### ORF12 Is the Founding Member of the GT136 Family.

The AlphaFold model of ORF12 predicts a large integral membrane domain with 11 transmembrane helices, a small predominantly α-helical domain located after transmembrane helix 1, and an additional domain rich in β-strand content located after transmembrane helix 11 toward the C terminus (*SI Appendix*, Fig. S5). The extramembrane domains are predicted by DeepTMHMM to be positioned in the periplasm. Notably, the extensive transmembrane helices, as well as the predicted periplasmic domain located after transmembrane helix 1, are shared with structures of GT enzymes possessing GT-C folds (*SI Appendix*, Fig. S6). Examples of GT-C fold enzymes include AglB (3WAK) (34), ALG6 (6SNI) (35), ArnT (5EZM) (36), EmbA, B (7BVG) and C (7BVH) (37, 38), Pmt1 and 2 (6P2R) (39), OST-A (6S7O) and B (6S7T) (40), PglB (3RCE) (41), AftA (8IF8) (42), and AftD (6W98) (43). The predicted structure of ORF12 suggests it catalyzes the final reaction in the pathway for Rib*f-*addition (Fig. 1*C*).

**Fig. 4.**
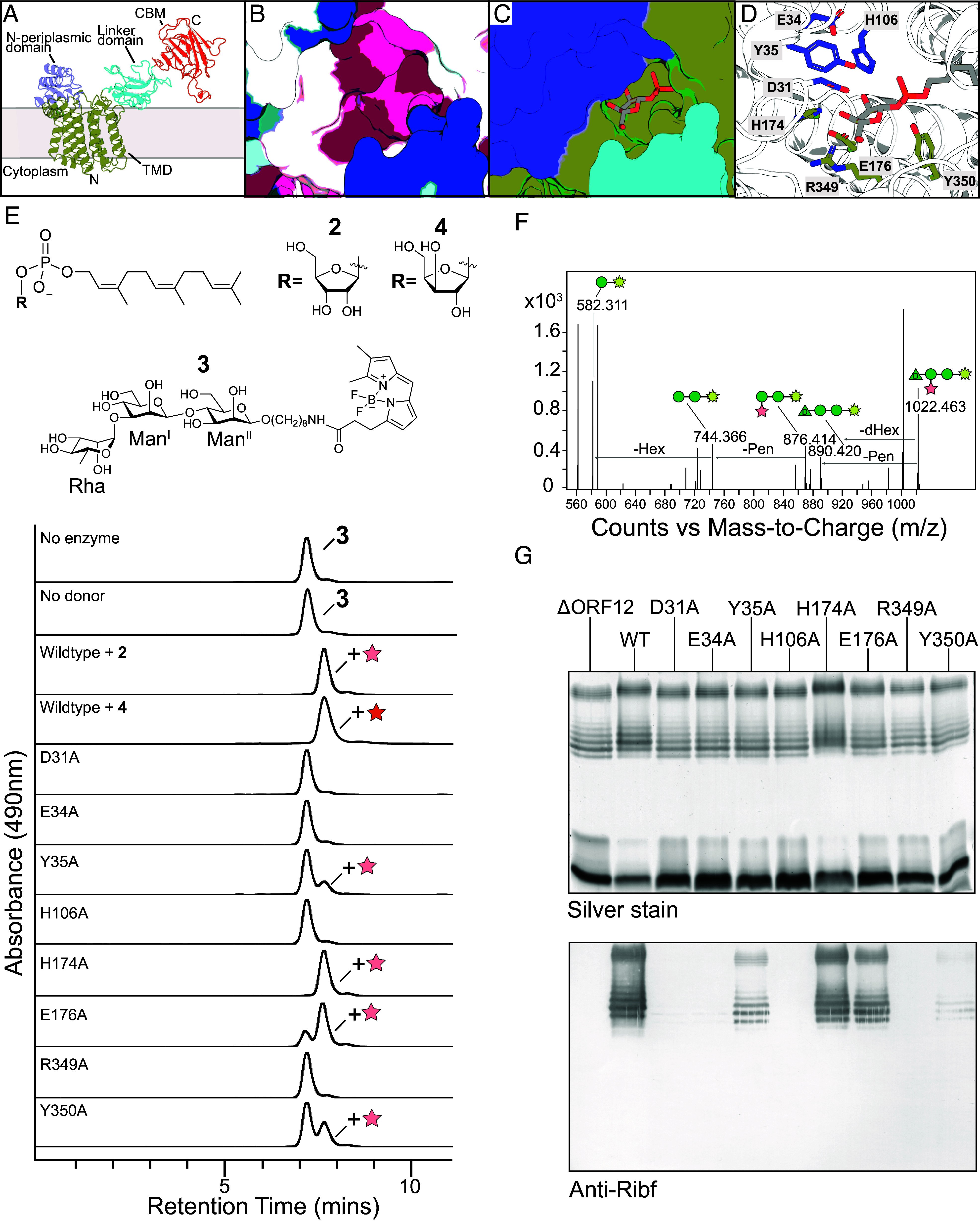
Predicted structure and activity of ORF12, a glycosyltransferase possessing a GT-C fold. (*A*) Each structural domain in ORF12 is colored separately for clarity. While the structures predicted for the domains can be viewed with some confidence based on crystal structures of related domains, the relative positioning of the GT-C enzyme and the CBM should be interpreted with caution. (*B*) A conserved pocket of ORF12 colored according to residue conservation with ConSurf, created using the multiple-sequence alignment shown in *SI Appendix*, Fig. S7. The most conserved regions are in purple, while blue shows regions that are not conserved. (*C*) A model of the proposed GT-C active-site groove containing undP-Rib*f* (as sticks), with ORF12 presented as a surface from the perspective of top–down toward the TM domain. (*D*) Proposed active site with conserved residues shown as sticks. The marked residues were all targeted for alanine replacement. (*E*) HPLC separation of in vitro reactions performed with membrane preparations as a source of enzyme for wild-type ORF12 or selected alanine variants with synthetic farnesylP-Rib*f* (**2**) donor and BODIPY-tagged O1 repeat unit mimic as the acceptor (**3**). The activity of wild-type ORF12 was also tested with synthetic farnesylP-Xyl*f* (**4**) as donor. The reaction products were separated using a GlycoSep N column with normal phase separation. (*F*) The later migrating product peak in HPLC was confirmed by MS/MS to reflect the addition of a pentose to Man^I^ (the internal Man) of **3** (shown as a cartoon with the yellow star representing the BODIPY tag and sugars represented using the SNFG system) (*G*) The same mutants were tested for in vivo activity by genetic complementation of the *C. youngae* O1 Δ*orf12* mutant. Whole-cell lysates were examined by silver-stained SDS-PAGE and western immunoblotting with antiserum specific for the Rib*f* side-chain.

To verify the function of ORF12 in Rib*f* transfer, the FPR donor (**2**) was used with a fluorescently labeled acceptor mimic (**3**). Membrane preparations containing wild-type ORF12 were incubated with **2** and **3** and the reaction mixture was separated by HPLC. A product distinct from **3** was observed (Fig. 4*E*) and mass spectrometry of the reaction mixture showed a major species with *m/z* 1022, consistent with the addition of ribose (exact mass 132) to **3** (exact mass 889) (*SI Appendix*, Fig. S3*B*). Furthermore, MS/MS analysis showed a fragment with *m/z* 876, consistent with the fragmentation loss of the terminal D-Rha (Fig. 4*F*). This fragment, together with the lack of a detectable fragment at *m/z* 714 (which would be expected in Rib*f* was linked to Man^II^), is consistent with the presence of Rib*f* being added to Man^I^ (the internal Man of the synthetic mimic). This is identical to the authentic O1 OPS natural product (Fig. 2*A*). The verification of function from ORF12, along with its lack of sequence similarity to current GT families, resulted in it becoming the founding member of the new GT136 family in the CAZy database.

Multiple sequence alignment of ORF12 orthologs found in gene clusters from candidate pentofuranose-modified glycan producers (*SI Appendix*, Fig. S7), combined with AlphaFold modeling, revealed a groove containing conserved residues located between the first periplasmic domain and the upper part of the transmembrane domain (Fig. 4*A* and *SI Appendix*, Fig. S5). This proposed active site location is consistent with other GT-C enzymes with experimentally solved structures (*SI Appendix*, Fig. S6). The closest example (from a sequence perspective) is ArnT, which performs the addition of Ara4N from undP-Ara4N to lipid-A in the periplasm, and was crystallized in the presence of undP (36). The ArnT structure shows the phospholipid emerging from a hydrophobic hole in the enzyme, and a hydrophilic pocket that makes contact with the phosphate head group. Additional residues in ArnT are appropriately positioned to bind to the Ara4N portion of the native undPP-Ara4N donor. To probe the importance of the corresponding region for activity in *C. youngae* O1 ORF12, substrate modeling was performed using the structure of ArnT with bound undP as a precedent (Fig. 4 *A–D*). The undecaprenyl portion of the donor can be accommodated by a hole formed above transmembrane helix 7 (Tα7) and Tα12 and below a loop following Tα9 (*SI Appendix*, Fig. S5). This hole is in a similar position in ORF12 and ArnT. A large hydrophobic patch located outside the hole could accommodate the lipid tail of the donor in a similar manner to ArnT. In the ORF12 model, Y350 and H106 are suitably positioned to coordinate the phosphate of undP-Rib*f*. In this scenario, Y35, H174, and R349 would make contacts with the hydroxyl groups of Rib*f*. In several experimentally solved GT-C structures, the proposed catalytic Asp is located after the first transmembrane helix and, in ORF12, D31 may fulfill this role. It precedes Tα1 and is located in proximity to C-1 of Rib*f* in the substrate model, to facilitate deprotonation of the incoming C-4 hydroxyl of the acceptor Man*p*.

To test the importance of conserved residues, alanine-replacement variants were generated and the mutated genes were used for complementation studies in *C. youngae* O1 Δ*orf12* (Fig. 4*G*). OPS produced by cells expressing OFR12^H174A^ or ORF12^E176A^ showed reactivity with Rib*f*-specific antiserum, comparable to the wild-type. The ORF12^Y35A^ mutant produced markedly less Rib*f* modification, and a trace amount of reactivity was retained by ORF12^Y350A^. In contrast, ORF12^D31A^, ORF12^E34A^, ORF12^H106A^, or ORF12^R349A^ all showed no antiserum reactivity when expressed in vivo. To corroborate the in vivo results, in vitro reactions were conducted using **2** and **3** as substrates (Fig. 4*E*). The results were consistent with the in vivo phenotypes. As expected, the addition of Rib*f* depended on the presence of FPR (**2**).

### ORF12^C^ Is a CBM Involved in Substrate Binding.

The AlphaFold model of ORF12 also contains a domain rich in β-strands, typical of an immunoglobulin-like fold found in carbohydrate binding modules (CBMs) (Fig. 4*A*). CBMs are found in many enzymes involved in glycan processing and synthesis and are typically used to bind their substrates (44). Examples include other GT-C enzymes, including EmbA, B, and C and AftD from mycobacterial arabinan biosynthesis (*SI Appendix*, Fig. S6). In EmbC and AftD, the CBMs were shown to interact with the acceptor polysaccharide, and in EmbC, the CBM is important for catalysis, with CBM mutants producing substantially less and smaller length arabinan (38, 43, 45). To test the hypothesis that the ORF12 CBM domain was also involved in substrate binding, an in vitro binding assay was performed using purified CBM (ORF12^503–652^) and LPS from *C. youngae* O1 Δ*orf15* LPS (*SI Appendix*, Fig. S8*A*). This experimental strategy was used previously to investigate the specificity of CBMs in OPS ABC transporters for their nascent polysaccharide substrates (46). ORF12^503–652^ was capable of binding to the *C. youngae* O1 Δ*orf15* LPS but did not bind the control LPS (*SI Appendix*, Fig. S8*A*). To assess the importance of the ORF12 CBM in OPS modification in vivo, a truncated version of ORF12 (ORF12^1–502^) lacking the CBM was used to transform *C. youngae* O1 Δ*orf12* (*SI Appendix*, Fig. S8*B*). Cells expressing ORF12^1–502^ produced ribosylated OPS, evident by the reactivity in western immunoblotting and ORF12^1–502^ retained in vitro activity (*SI Appendix*, Fig. S8*C*). Collectively, these results indicate that ORF12^503–652^ is capable of binding deribosylated OPS, but the CBM is not essential for the function of ORF12 in the ribosylation reaction under the conditions tested.

### ORF13 Is an Epimerase Dictating the Identity of the Pentofuranose Side-Chain.

In the biosynthesis of mycobacterial cell walls, DprE1 and DprE2 are epimerases that convert decP-Rib*f* to decP-Ara*f* (Fig. 1*B*). Although classically thought to be a cytoplasmic reaction, studies with spheroplasts and labeling with fluorescent probes led to a proposed extracytoplasmic location for DprE1 (47, 48). As noted by the authors, it is uncertain how proteins lacking N-terminal signal sequences access this location, so the precise location is still subject to debate. The O2 cluster contains an additional gene, *orf13,* predicted to encode a cytoplasmic short-chain dehydrogenase/reductase (SDR) epimerase. The sequence of ORF13 was aligned with the well-characterized SDR representative UDP-glucose 4-epimerase (GalE; a reversible enzyme that epimerizes UDP-glucose to UDP-galactose). The alignment revealed conserved TGXXGXXG and YXXXK motifs used by GalE to bind NADH and catalyze inversion, respectively (*SI Appendix*, Fig. S9). Serotype O1 retains remnants of the O2 gene sequences, suggesting loss of the *orf13* underlies the different side chains in O1 (Rib*f*) and O2 (Xyl*f*). To test this, the chromosomal *orf13* gene in O2 was replaced with a kanamycin-resistance cassette and the LPS was examined. SDS-PAGE of whole-cell lysates resulted in no obvious change in the LPS banding pattern from wild-type *C. youngae* O2 (*SI Appendix*, Fig. S10); this was not surprising given the change from Xyl*f* to Rib*f* would not generate an altered mass. The OPS structure from the *orf13* mutant was then determined by NMR spectroscopy and chemical shift data from two-dimensional ^1^H, ^13^C HSQC experiments were compared to the published data for *C. youngae* O1 and O2 (13, 14) (*SI Appendix*, Fig. S1*C* and Table S2). The chemical shifts for the *orf13* mutant revealed ribosylated *C. youngae* O1 OPS (13). Transformation of the *orf13* mutant with a plasmid carrying the *orf13* gene restored the *C. youngae* O2 structure (14). These data supported the epimerase assignment and indicated that ORF13 alone determines the identity of the pentofuranose modification, but not its addition to the repeating unit. To confirm the latter, the in vitro activity of ORF12 was tested using farnesylP-Xyl*f* (**4**) as a donor (Fig. 4 and *SI Appendix*, Fig. S3). The positive result showed ORF12 is promiscuous toward other pentoses and explains its conservation in serotypes O1 and O2.

## Discussion

The incorporation of pentofuranoses into bacterial polysaccharides represents a mechanistic challenge not seen with most other sugars, such as hexoses. NDP-sugar-dependent Leloir GT enzymes can operate via either inverting or retaining activities, resulting in products with α- or β-linked sugars. In contrast, the prototypical PRPP-dependent Rib*f* transferases previously identified are limited to the incorporation of β-linked Rib*f* due to their inverting mechanism and must operate in the cytoplasm to access the PRPP donor. The addition of α-linked Rib*f* requires either a GT with a retaining mechanism or a fundamentally different immediate donor. Here, we establish that the latter approach is used in a range of bacterial species and different glycans; the donor is undP-Rib*f* and it participates in a postpolymerization periplasmic modification system. Prototypes for periplasmic postpolymerization modification systems were first identified in OPS biosynthesis systems from *Salmonella* and *Shigella* (4) but, in most cases, biochemical details for those pathways have not been reported. Here, we characterized the machinery introducing Rib*f* side chains (and by extension other pentofuranoses), based on a prototype system from *Citrobacter*. The model we propose retains similarities to the current archetypal periplasmic side chain glucosylation and galactosylation in gram-negative bacteria, but more closely resembles enzymes involved in arabinofuranose polymerization in mycobacterial cell wall biogenesis (Fig. 1).

Based on the data presented here, the pathway leading to the introduction of the pentofuranose side chains begins with the creation of the lipid donor undP-Rib*f* by sequential activities of the PPPRS and PRP domains of ORF15 (Fig. 1*C*). The PPPRS shares no similarity to the GtrB-type enzymes used in periplasmic side-chain hexose addition prototypes, which have a small (1 to 2) transmembrane helix anchor, and a cytoplasmic GT domain that uses a UDP-sugar as the donor (49). Instead, ORF15 resembles mycobacterial DPPRS and *Pseudomonas* PPPRS. However, neither of these latter proteins possesses a PRP domain like ORF15 and instead requires a separate enzyme (Rv3807c in *M. tuberculosis*, or *P. aeruginosa* PA7_6246), belonging to different structural families, to dephosphorylate the product. Interestingly, a PPPRS candidate identified in the plant pathogen *Pseudomonas syringae* pv *syringae* B728a resembles ORF15 more closely; it contains a C-terminal PRP domain. This protein is predicted to also be involved in *O*-glycosylation of its pilin with Ara*f* (like the *P. aeruginosa* enzyme) (29), but no biochemical data are available. Regardless of the domain identity and organization, the final reaction product in all of the systems is known (or predicted) to be polyprenyl phosphate-linked Rib*f*. The newly released structure of the mycobacterial DPPRS provides important insight into the reaction mechanism (23). While the und lipid is shared by both pathways, the use of undP-linked donors for periplasmic modification offers segregation from undPP-linked oligo- and polysaccharides participating in the central pathways for microbial glycan synthesis, where the main glycosylation reactions occur in the cytoplasm.

We provide compelling evidence for additional enzymes that convert Rib*f* precursors to other pentoses for subsequent incorporation into OPS. This process is analogous to the two-enzyme system (DprE1/E2), which converts DPR to DPA in mycobacteria. We are not aware of any other examples of epimerase enzymes in OPS side-chain glycosylation systems. The sequence of *ORF13* predicts an epimerase, consistent with the need to convert undP-Rib*f* to undP-Xyl*f*. ORF13 lacks a signal sequence, indicating it operates in the cytoplasm. Epimerization is expected to occur after the transfer of Rib*f* to undP (and before export) because PRPP is used in nucleotide biosynthesis and epimerization of PRPP itself is expected to be detrimental. As indicated above, there are some data suggesting that the analogous epimerases in *M. tuberculosis* operate outside of the cytoplasm (48), but a mechanism to get these proteins (and the needed redox cofactor) to this location is not apparent and further investigation is warranted.

Searches for Rib*f* epimers (Xyl*f*, Lyx*f*, and Ara*f*) present in other polysaccharides identified further candidate systems with Rib*f*, Xyl*f,* and Ara*f* that have the necessary three-component modification machinery (*SI Appendix*, Fig. S11); Lyx*f* is not found in any solved carbohydrate structures. In all isolates where pentofuranose side chain additions and a genome sequence were available, the anticipated candidate three-component glycosylation system was identified. The candidate modification systems contained an ORF13 homolog in the case of Xyl*f*-containing glycans, or DprE1/E2 homologs for those containing Ara*f* (*SI Appendix*, Fig. S11). We postulate that a common entry point for all of these pentofuranoses is the pathway we have elucidated here, involving the synthesis of undP-Rib*f* from PRPP and subsequent epimerization of the C2 and/or C3 hydroxyl groups. For Ara*f* and Xyl*f*, a single epimerization is required at C2 or C3, respectively. The requirement to invert both the C2 and C3 stereocenters for Lyx*f* could explain why no examples of Lyx*f* containing polysaccharides are (currently) known. In all but one example (see below), the pentofuranose side-chains were α-linked, but this pathway does not preclude the addition of β-linked side chain pentofuranoses. For the addition of β-linked pentofuranose side chains, the GT-C of the system would adopt a retaining (as opposed to inverting) mechanism and precedent is provided by AftB from *M. tuberculosis*, which has been implicated in the addition of terminal β-linked Ara*f* residues of arabinogalactan (50). Notably, no other examples of β-linked side chain Ara*f* or Xyl*f* were identified, and all β-linked Rib*f* residues we identified were accounted for by direct incorporation from PRPP-utilizing enzymes identified previously (2).

After epimerization, the undP-linked donor must be flipped to the periplasm where it can be used for side-chain modification. Bioinformatics and modeling suggest that ORF14 is a MATE family protein responsible for this function. In contrast, in *M. tuberculosis*, the proposed Rv3789 transporter responsible for export of DPR is a small multidrug-resistant (SMR)-family transporter, like those used in the Glc/Gal OPS side-chain prototypes (51). Direct experimental evidence for flippase activity has not been shown for these proteins and was not pursued in this study. However, bioinformatic analysis combined with the genetic context of ORF14 strongly implicates its role as a undP-Rib*f* flippase.

Finally, we determined the activity of a member of the GT-C family of enzymes, forming the new GT136 family. The amount of similarity shared by different GT-C members varies substantially. Two structural subclasses of GT-C enzymes (GT-C_A_ and GT-C_B_) have been proposed (52). At the time of the proposal, the GT-C_A_ fold was represented in 5 GT families and GT136 can now be added to this group. Fewer examples possess a GT-C_B_ fold. However, recent sequence-based classification suggests the diversity of the GT-C family may be more expansive, with two sequence clans forming outside the GT-C_A_ or GT-C_B_ fold clans, and additional outliers present (53). At the most fundamental level, GT-C enzymes have a 7 to 13 transmembrane helical domain and a small extracytoplasmic domain (usually located after TM helix 1), which is responsible for binding and catalyzing sugar additions using lipid-linked donors (54). Some GT-C enzymes have more elaborate structures that include additional extracytoplasmic domains, such as the CBM domain in ORF12 identified here, that bind the unmodified O1 OPS backbone. CBMs are found in other GT-C enzymes, including the arabinosyltransferases, EmbA, B, and C as well as AftD (38, 43, 45). The EmbC CBM was shown to be required for efficient enzyme activity, yet some arabinan was still made in all of the CBM mutants tested in vitro and in vivo, while the CBM from ORF12 appears to be disposable for catalysis, at least under the conditions tested. The precise physiological role of the ORF12 CBM is therefore presently unclear. The CBM may act to increase the local concentration of the ORF12 substrate enhancing catalysis. Expression of plasmid-encoded genes is expected to increase the amount of the enzyme in the membrane (relative to chromosomal copy) and this higher abundance of a CBM-less protein may serve to mask inefficiencies in the truncated enzyme. This question can only be unequivocally resolved using purified enzyme and natural (undPP-glycan) acceptor substrate and is not feasible at this time.

A key feature of ORF12 is its apparent promiscuity for the use of Rib*f* or Xyl*f* donors. This may reflect the cellular (serotype) background, where only one donor is typically available, and there is no need to discriminate between different (potentially competing) substrates. Substrate differentiation is assumed to be more important for cytoplasmic Leloir enzymes, which are potentially exposed to multiple NDP sugars. Nevertheless, relaxed specificity has been observed in some Leloir glycosyltransferases. For example, the eukaryotic β-(1→4)-galactosyltransferase-1 (β4GalT1) typically attaches galactose to a GlcNAc acceptor, but it can also use UDP-Glc and UDP-GalNAc donors, albeit with a substantially reduced efficiency compared to UDP-Gal (55). In the context of postpolymerization OPS modifications, the lack of donor specificity in ORF12 potentially affords facile expansion of serotype diversity by addition of different pentoses when epimerase genes are acquired or lost. It is currently unclear whether other GT-C enzymes from different glycosylation systems also have some donor promiscuity.

Structural diversity in bacterial glycans arises from many different factors, including mutations that affect GT activity and specificity, genetic transfer and integration of full or partial gene clusters, and auxiliary extracytoplasmic modification systems. The extracytoplasmic modification systems alter the immunochemistry of the bacterial surface, creating additional epitopes, while masking others. Here, we have described several previously unrecognized systems for the addition of pentofuranoses and bioinformatic searches indicate that this is a widely used strategy for glycan diversification. Using ORF15 as a search probe, we also identified additional candidate pentofuranose glycosylation systems in bacterial species where the glycoconjugate type and carbohydrate structure remain unknown. These include examples from gram-negative and gram-positive bacteria and one example encoded by the genome of a bacteriophage belonging to the *Caudoviricetes* (*SI Appendix*, Fig. S12). The latter could facilitate phage-mediated seroconversion, similar to the classical *Shigella* and *Salmonella* paradigms (4). More elaborate systems containing multiple candidate epimerases or GT-Cs were found in examples from *Xanthomonas* and in a gram-positive representative belonging to the genus *Bacillus*. The functional significance of this complexity warrants investigation. While the use of the three-component system described here is widespread, a different method for pentofuranose side-chain addition is used for the incorporation of the modified xylofuranose (5′-methyl-thioxylose or MTX). MTX is added as a capping motif in the *M. tuberculosis* lipoarabinomannan (56). Mtx is added in an extracytoplasmic reaction, using a dec-P-linked donor and a GT-C enzyme. However, the initial donor in the pathway is the adenosine metabolite 5′-methyl-thioadenosine, rather than PRPP. It is unclear whether this modification reaction exists outside lipoarabinomannan assembly.

In conclusion, this study provides a prototype for a widespread antigenic diversification strategy and criteria for predicting side-chain modification reactions in glycans from other organisms, where those glycans play important biological roles. It also expands the toolbox of enzymes that may be deployed in glycoengineering and recombinant production of polysaccharides with pentofuranose side chains for glycoconjugate/vaccine purposes.

## Experimental Procedures

Detailed methods are provided in *SI Appendix*.

### Bacterial Strains, Plasmids, and Growth Conditions.

Bacterial strains and plasmids used in this study are listed in *SI Appendix*, Table S3. Cultures were grown in lysogeny broth (LB) with antibiotics used where appropriate (34 μg/mL chloramphenicol, 25 μg/mL kanamycin). Chromosomal mutations were introduced by Lambda red recombination as described previously (57). Recombinant plasmids were constructed using linear PCR-amplified DNA fragments and site-directed changes were introduced by inverse PCR of plasmids using primers including the desired mutation, which were designed in NEBasechanger (New England Biolabs). Gene expression of pBAD-based constructs in mutant complementation studies relied on leaky expression (no inducer added) and protein purification was performed from cultures grown with 0.2% ʟ-arabinose.

### Antiserum Production.

Rabbit antiserum recognizing *C. youngae* O1 was produced using formalin-killed *C. youngae* O1 PCM1492 cells as the antigen. To prepare antiserum specific for the Rib*f-*modification, the O1 antiserum was adsorbed using *C. youngae* O1 PCM1492 Δ*orf12* cells.

### SDS-PAGE and Immunoblotting.

Proteinase K-digested whole-cell lysates were used for examination of LPS by SDS-PAGE as described previously (58). Samples were separated on 12% gels with Tris-glycine buffer. LPS samples were separated on 12% gels with Tris-glycine buffer and were visualized by silver staining or probed by western immunoblotting using rabbit anti-O1 and Rib*f*-specific antiserum. The secondary antibody was goat anti-rabbit-conjugated alkaline phosphatase and detection was performed using nitroblue tetrazolium and 5-bromo-4-chloro-3-indolyl phosphate (Roche Applied Science). Proteins were analyzed by SDS-PAGE and Coomassie blue staining.

### Purification of OPS.

LPS was purified using the hot water-phenol method from cells grown in 10 L cultures (59). LPS was hydrolyzed at 100 °C in 2% (v/v) acetic acid and the released OPS was purified by chromatography on a Sephadex G-50 superfine column eluted in 50 mM pyridinium acetate buffer (pH 4.5). Fractions containing OPS were pooled and lyophilized.

### In Vitro Glycan Synthesis Assays.

GT activities were examined in reactions containing either bacterial membranes or ORF12^503–652^-His_6_ purified by affinity chromatography on Ni-NTA resin. Reactions were performed in 20 μL volumes containing 50 mM HEPES pH 7.5, 20 mM MgCl_2_, and 5 mM PRPP where applicable. For PPPRS reactions, 1 mM of **1** [(2*E*,6*E*)-farnesylP] was used as an acceptor, prepared as in ref. 30. For ORF12 reactions **2** [(2*Z*,6*Z*)-farnesylP-β-D-Rib*f*] or **4** [(2*Z*,6*Z*)-farnesylP-β-D-Xyl*f*] were used as donor for reaction with **3** as an acceptor (*SI Appendix*, Figs. S13–S15). Incubation was performed at 30 °C for 18 h and reactions were stopped by adding of an equal volume of cold acetonitrile. FarnesylP-Rib*f* for NMR, a large-scale reaction was performed in a (6.6 mL) reaction and purified for NMR by separation using silica gel.

The reaction mixtures were separated by normal phase HPLC as described previously (2, 60), using an Agilent 1260 Infinity II LC system equipped with a GLYCOSEP N column (4.6 × 250 mm, Prozyme). Solvent A contained 10 mM ammonium formate pH 4.4 in 80% acetonitrile, solvent B contained 30 mM ammonium formate pH 4.4 in 40% acetonitrile, and solvent C contained 0.5% formic acid. All HPLC analysis was performed using OpenLAB revision A.02.16 (Agilent). TLC was performed on aluminum foil silica gel 60 F_254_ TLC plates (EDM Millipore). Separated molecular species were detected by charring and imaged with a ChemiDoc (BioRad).

### Mass Spectrometry.

LC–MS was performed with an Agilent 1260 HPLC liquid chromatograph interfaced with an Agilent UHD 6530 Q-TOF mass spectrometer in the University of Guelph Advanced Analysis Centre. The mass-to-charge ratio was scanned across the m/z range of 50 to 1,500 m/z in 4 GHz (extended dynamic range) positive ion mode. The acquisition rate was set at 2 spectra/s.

### NMR Spectroscopy.

NMR analysis of OPS and in vitro reaction products was performed at Advanced Analysis Centre, University of Guelph. Spectra for deuterium exchanged OPS samples were recorded in 99.9% D_2_O with chemical shifts referenced to a 3-trimethylsilylpropanoate-2,2,3,3-d_4_ (δ_H_ 0 ppm, δ_C_ –1.6 ppm) internal standard. NMR analysis of farnesylP-Rib*f* was performed in MeOD, at 22 °C with trace MeOH as an internal standard.

### CBM-Mediated Binding Assay.

LPS pulldown assays were performed as described previously (46). One milliliter reaction volumes containing 200 μg of LPS and 200 μg of ORF12^503–652^-His_6_ were incubated on a rotary shaker for 30 min at room temperature and protein was recovered using PureProteome Nickel Magnetic Beads (Millipore). Protein was eluted from the beads with 500 mM imidazole and examined by silver-stained SDS-PAGE and western immunoblotting.

### Bioinformatics Analyses.

Multiple sequence alignments were performed in ClustalW (61) and TCoffee (62) (where indicated) and visualized using ESPript (63). Transmembrane helix prediction was performed using DeepTMHMM (64) and structural models were made using AlphaFold through the Colabfold notebook (65, 66). All AlphaFold models were individually assessed and contained pLDDT values greater than 80 across the majority of the proteins, classifying them as confident models. Structural conservation was shown using Consurf (67) and 3D structure alignments were performed using Dali (68). Protein visualization was performed in PyMol v2.5.4.

## Supplementary Material

Appendix 01 (PDF)

## Data Availability

All study data are included in the article and/or *SI Appendix*.
