# Supplementary material for: Three-component systems represent a common pathway for extracytoplasmic addition of pentofuranose sugars into bacterial glycans
Source: Proc Natl Acad Sci U S A. 2024 May 15;121(21):e2402554121. doi: 10.1073/pnas.2402554121 (PMC11127046; doi:10.1073/pnas.2402554121)
Supplement: Supplementary file 1 — Appendix 01 (PDF) [file pnas.2402554121.sapp.pdf]

SUPPLEMENTARY INFORMATION

Three-component systems represent a common pathway for extracytoplasmic addition of pentofuranose sugars into bacterial glycans

Steven D. Kelly[1], Nam Ha Duong[†,2,5,6], Jeremy T. Nothof[†,3], Todd L. Lowary*[2,3,4] and Chris Whitfield*[1]

[1]Department of Molecular and Cellular Biology, University of Guelph, Guelph, Ontario N1G 2W1, Canada
[2]Institute of Biological Chemistry, Academia Sinica, Academia Road, Section 2, #128, Nangang, Taipei, 11529, Taiwan
[3]Department of Chemistry, University of Alberta, Edmonton, Alberta, Canada T6G 2G2
[4]Institute of Biochemical Sciences, National Taiwan University, Section 4, #1, Roosevelt Road, Taipei 10617, Taiwan
[5]Chemical Biology and Molecular Biophysics, Taiwan International Graduate Program, Academia Sinica, Academia Road, Section 2, #128, Nangang, Taipei 11529, Taiwan
[6]Department of Chemistry, National Tsing Hua University, Hsinchu 300044, Taiwan

[†]Authors contributed equally
Corresponding authors
Todd L. Lowary. Email: tlowary@gate.sinica.edu.tw
Chris Whitfield. Email: cwhitfie@uoguelph.ca.

Detailed experimental procedures

Construction of recombinant plasmids and mutant strains. KOD Hotstart polymerase (Novagen) was used to generate linear fragments by PCR. Oligonucleotide primers (obtained from Integrated DNA Technologies) are listed in Table S4. PCR products and plasmids were purified with the GeneJET purification kit and GeneJET plasmid miniprep kit, respectively (Fisher Scientific). Construction of site directed mutants was accomplished by inverse PCR of plasmids using primers including the desired mutation, which were designed in NEBasechanger (New England Biolabs). The linear fragment was then treated with DpnI, T4 polynucleotide kinase, and T4 DNA ligase, according to manufacturer's recommendations, and transformed into *E. coli* DH5α. The sequences of plasmid constructs were confirmed at the Advanced Analysis Centre, Genomics Facility, University of Guelph.

Antiserum production. Rabbit antiserum recognizing *C. youngae* O1 was produced using formalin-killed *C. youngae* O1 PCM1492 cells as the antigen. Cells were suspended in 0.85% (w/v) NaCl at ~10^8 cfu/ml and mixed 1:1 with Freund's incomplete adjuvant (Sigma) and injections were performed intramuscularly in a New Zealand white rabbit every two weeks for 6 weeks total. After 6 weeks blood was collected and the antiserum was stored at $^-$80°C. Animal handling and immunization was performed at the University of Guelph Central Animal Facility. To prepare antiserum specific for the Rib*f*-modification, the O1 antiserum was adsorbed using *C. youngae* O1 PCM1492 Δ*orf12* cells. Cells from 200 mL of overnight culture were collected and resuspended in 16 mL sterile PBS and divided into 4 equal aliquots. Cells were collected from one aliquot and the pellet was resuspended in 5.2 mL of antiserum and incubated at 37°C for 1 hour before the cells were removed. The process was repeated with the remaining 3 aliquots. The adsorbed antiserum was then frozen in aliquots at $^-$20°C.

SDS-PAGE and immunoblotting. Proteinase K-digested whole-cell lysates were used for examination of LPS by SDS-PAGE as described previously (1). An amount of cells equivalent to 1 A_{600nm} unit was collected and resuspended in loading buffer, heated at 100°C for 10 minutes, and subsequently treated with proteinase K at 55°C for 1 hour. Samples were separated on 12% gels with Tris-glycine buffer. LPS was visualized by silver staining or by western immunoblotting after transfer to nitrocellulose membranes

(Protran, GE Healthcare). Transfer was performed using a constant current of 200 mA for 45 minutes in buffer containing 25 mM Tris, 150 mM glycine, and 20% (v/v) methanol. Membranes were blocked in 5% skim milk (BD Difco) prepared in TBST (10 mM Tris-Cl, pH 7.5, 150 mM NaCl, 0.005% (v/v) Tween 20). Rabbit anti-O1 and Rib*f*-specific antiserum were used at 1:1000 dilutions in TBST containing 5% (w/v) skim milk powder. Goat anti-rabbit-conjugated alkaline phosphatase (1:3000) (Cedar Lane) was used as a secondary antibody and detection was performed using nitroblue tetrazolium and 5-bromo-4-chloro-3-indolyl phosphate (Roche Applied Science). Proteins were analyzed by mixing samples with an equal volume of loading buffer and separating by SDS-PAGE as above. Detection was achieved using Coomassie blue staining using the Pierce Power Stainer. The SeeBlue Plus2 protein ladder provided molecular weight markers (Invitrogen).

Purification of OPS. LPS was purified using the hot water-phenol method from cells grown in 10 L cultures (2). After phase-separation, the LPS-containing aqueous fraction was dialyzed against water to remove residual phenol. Proteins and nucleic acids were removed by precipitation using cold, aqueous trichloroacetic acid followed by centrifugation at 12,000 × *g* for 20 min. The supernatant was then dialyzed against water until neutral pH was achieved and the solution was concentrated in a rotovap and the LPS was lyophilized. OPS was isolated by hydrolysis of purified LPS at 100 °C in 2% (v/v) acetic acid, followed by centrifugation to remove the lipid precipitate. The carbohydrate-containing supernatant was then separated on a Sephadex G-50 superfine column (2.5 cm × 75 cm) in 50 mM pyridinium acetate buffer (pH 4.5) at a flow rate of 0.6 ml/min. Elution was monitored with a Smartline 2300 refractive index detector (Knauer) and fractions were collected at 10 min intervals. Fractions containing to the OPS peak were combined and concentrated.

Membrane preparation and protein purification for in vitro investigations. To prepare membranes for *in vitro* reactions, cultures were grown to A_{600nm} 0.6 and expression of plasmid-encoded proteins was induced overnight at 18°C following addition of 0.2% L-arabinose. Cells were collected by centrifugation and resuspended in buffer containing 50 mM HEPES, 500 mM NaCl at pH 7.4, prior to lysis by sonication. Lysates were cleared by centrifugation at 12,000 × *g* for 20 min, and membranes were collected by

centrifugation of the supernatant at 100,000 × *g* for 1 h. Membranes were resuspended in buffer A with 150 mM NaCl and total protein concentration was measured using the DC protein assay (Bio-Rad). ORF12$^{503\text{-}652}$-His$_6$ was purified by passing cleared cell lysate through a column with Ni-NTA resin. The column was washed using buffer A with 10 mM imidazole, followed by a second wash with buffer A containing 30 mM imidazole. Protein was eluted using buffer A containing 250 mM imidazole. Purified ORF12$^{503\text{-}652}$-His$_6$ was buffer exchanged into buffer B (50 mM HEPES, 150 mM NaCl at pH 7.4) and concentrated with a Vivaspin 20 with a 10 kDa molecular weight cut-off. Protein concentration was estimated using a nanodrop with a theoretical extinction coefficient obtained from Protparam (https://web.expasy.org/protparam/).

In vitro glycan synthesis assays. Reactions were performed in 20 µL volumes containing 50 mM HEPES pH 7.5, 20 mM $MgCl_2$, and 5 mM PRPP where applicable. For PPPRS reactions, 1 mM of **1** ((2*E*,6*E*)-farnesylP) was used as an acceptor, prepared as in (3). For ORF12 reactions **2** ((2*Z*,6*Z*)-farnesylP-β-D-Rib*f*) or **4** ((2*Z*,6*Z*)-farnesylP-β-D-Xyl*f*) were used as donor for reaction with **3** as an acceptor (Figure S13,S14,S15). Reactions contained aliquots of membrane containing 20 µg of total protein. Reaction mixtures were incubated at 30 °C for 18 hrs. Reactions were stopped by the addition of an equal volume of cold acetonitrile and precipitated protein was removed by centrifugation at 12,000 × *g* for 5 min. For purification of farnesylP-Rib*f* for NMR, a large-scale reaction was performed in a 6.6 mL reaction. The reaction mixture was dried and resuspended in methanol and separated using silica gel (pore size 60Å, 230–400 mesh particle size, 40–63 µm particle size) eluting in 1:1 methanol–ethyl acetate.

The reaction mixtures were separated by normal phase HPLC as described previously (4, 5), using an Agilent 1260 Infinity II LC system. 10 µL of sample was injected and separated with a GLYCOSEP N column (4.6 × 250 mm, Prozyme). Solvent A contained 10 mM ammonium formate pH 4.4 in 80% acetonitrile, solvent B contained 30 mM ammonium formate pH 4.4 in 40% acetonitrile, and solvent C contained 0.5% formic acid. The separation sequence was as follows: a linear gradient of 100% A to 100% B over 160 min (0.4 ml/min), followed by a 2 min gradient of 100% B to 100% C.; returning to 100% A over 2 min and holding for 15 min (1 ml/min); followed by 0.4 ml/min for 5 min in A. The column temperature was 30°C and elution was monitored at

260nm. All HPLC analysis was performed using OpenLAB revision A.02.16 (Agilent). TLC was performed by spotting 3 µL reaction mixture onto an aluminum foil silica gel 60 F_{254} TLC plate (EDM Millipore). TLC plates were developed using ethyl acetate:butanol:water:acetic acid (25:40:20:12.5). The plates were dried and then dipped in a solution of 3.7% (v/v) sulfuric acid and 1.1% (v/v) glacial acetic acid made in anhydrous ethanol and then heated using a hairdryer until dark blue spots appeared. TLC plates were then imaged with a ChemiDoc (BioRad).

Mass spectrometry. LC-MS was performed with an Agilent 1260 HPLC liquid chromatograph interfaced with an Agilent UHD 6530 Q-TOF mass spectrometer in the University of Guelph Advanced Analysis Centre. *In vitro* reaction mixtures were filtered using a Microcon-10 kDa centrifugal filter, or extracted with an equal amount of chloroform followed by centrifugation. Samples were separated on a C18 column (Agilent Poroshell 120, EC-C18 50 mm × 3.0 mm, 2.7 µm) with the following solvents: water with 0.1% formic acid (A) and acetonitrile with 0.1% formic acid (B). The mobile phase gradient was as follows: initial conditions were, 10% B for 1 min; increasing to 100% B in 29 min; column wash at 100% B for 5 min; followed by 20 min re-equilibration. The flow rate was maintained at 0.4 mL/min. The mass spectrometer electrospray capillary voltage was maintained at 4.0 kV, and the drying gas temperature at 250 °C, with a flow rate of 8 L/min. Nebulizer pressure was 30 psi and the fragmentor was set to 160. Nozzle, skimmer, and octapole RF voltages were set at 1000 V, 65 V and 750 V, respectively. Nitrogen (purity >98%) was used as nebulizing, drying, and collision gas. The mass-to-charge ratio was scanned across the m/z range of 50-1500 m/z in 4 GHz (extended dynamic range) positive ion mode. The acquisition rate was set at 2 spectra/s. The mass axis was calibrated using the Agilent tuning mix HP0321 (Agilent technologies) prepared in acetonitrile. Sample injection volume was 10 µL.

Nuclear magnetic resonance spectroscopy. NMR analysis of OPS and *in vitro* reaction products was performed at Advanced Analysis Centre, University of Guelph. All OPS samples subjected to NMR analysis were deuterium exchanged by lyophilizing twice with 99.0% D_2O. Spectra were recorded in 99.9% D_2O with chemical shifts referenced to a 3-trimethylsilylpropanoate-2,2,3,3-d_4 (δ_H 0 ppm, δ_C –1.6 ppm) internal standard.

NMR spectra were obtained at 50°C. NMR analysis of farnesylP-Rib*f* was performed in MeOD, at 22°C with trace MeOH as an internal standard.

CBM-mediated binding assay. LPS pulldown assays were performed as described previously (6). Briefly, 1 ml reaction volumes containing 200 µg of LPS and 200 µg of ORF12$^{503\text{-}652}$ in buffer B (25 mM Bis-Tris, pH 7.0, 250 mM NaCl) were incubated on a rotary shaker for 30 minutes at room temperature. This mixture was added to a 50 µL aliquot of PureProteome Nickel Magnetic Beads (Millipore), which were pre-equilibrated with buffer B, and incubated for 30 min at room temperature on a rotary shaker. The beads were collected with a magnet and washed three times with 500 µL of buffer B. Protein was eluted stepwise from the beads using three washes with 100 µL buffer B containing 500 mM imidazole. The loading, wash and elution fractions were examined by silver-stained SDS-PAGE and western immunoblotting.

Bioinformatics analyses. Multiple sequence alignments were performed in ClustalW (7) and TCoffee (8) (where indicated), and visualized using ESPript (9). Transmembrane helix prediction was performed using DeepTMHMM (10) and structural models were made using AlphaFold through the Colabfold notebook (11, 12). All AlphaFold models were individually assessed and contained pLDDT values greater than 80 across the majority of the proteins, classifying them as confident models. Structural conservation was shown using Consurf (13) and 3D structure alignments were performed using Dali (14). Protein visualization was performed in PyMol v2.5.4.

a

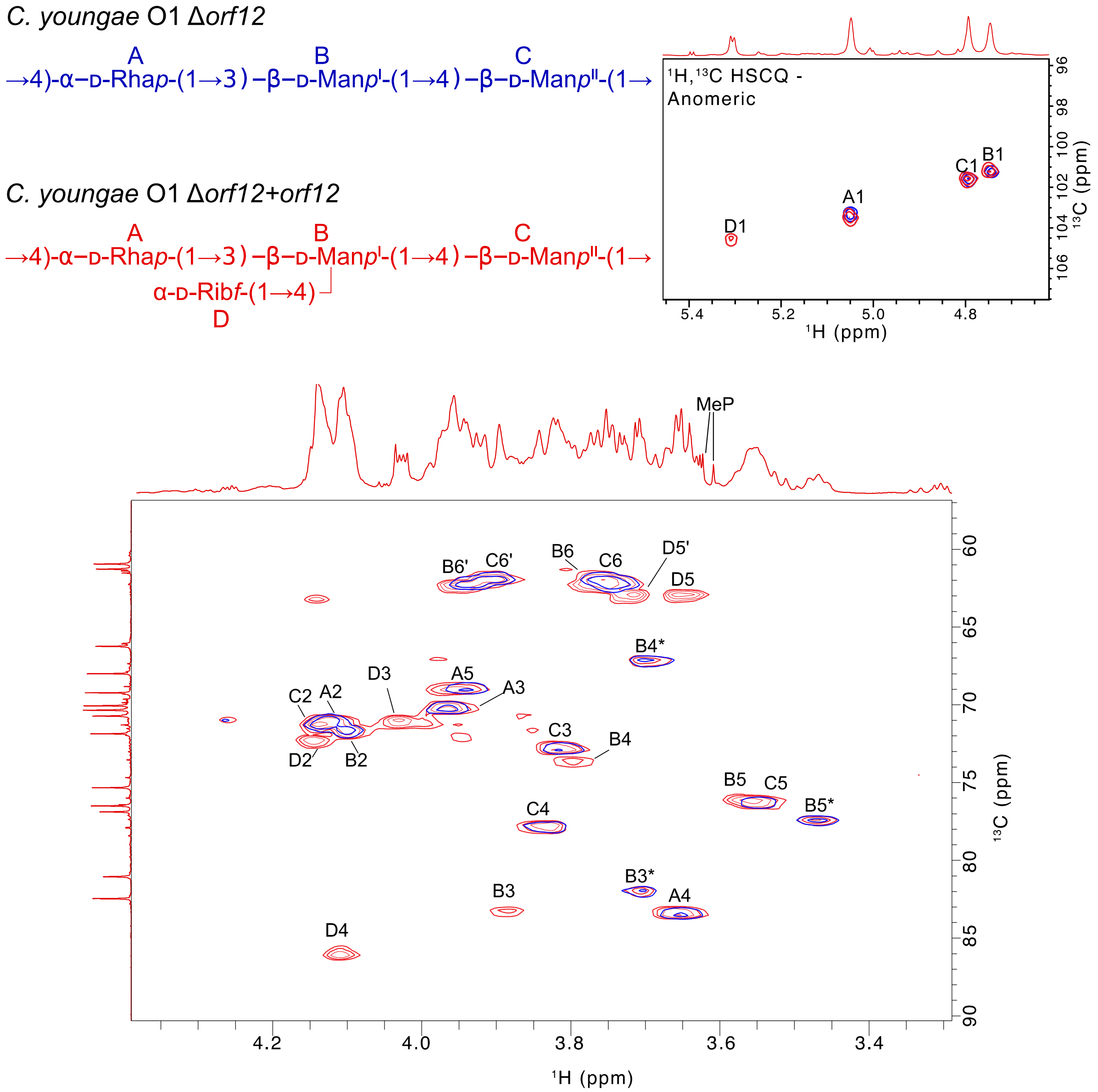

C. youngae O1 Δorf12
→4)-α-D-Rhap-(1→3)-β-D-Manp^I-(1→4)-β-D-Manp^II-(1→
A
B
C
C. youngae O1 Δorf12+orf12
→4)-α-D-Rhap-(1→3)-β-D-Manp^I-(1→4)-β-D-Manp^II-(1→
α-D-Ribf-(1→4)
D
1H,13C HSCQ - Anomeric
D1
A1
C1
B1
MeP
B6'
C6'
B6
C6
D5'
D5
B4*
D3
A5
A3
C2
A2
D2
B2
C3
B4
B5
C5
B5*
C4
B3*
A4
B3
D4
1H (ppm)
13C (ppm)

b

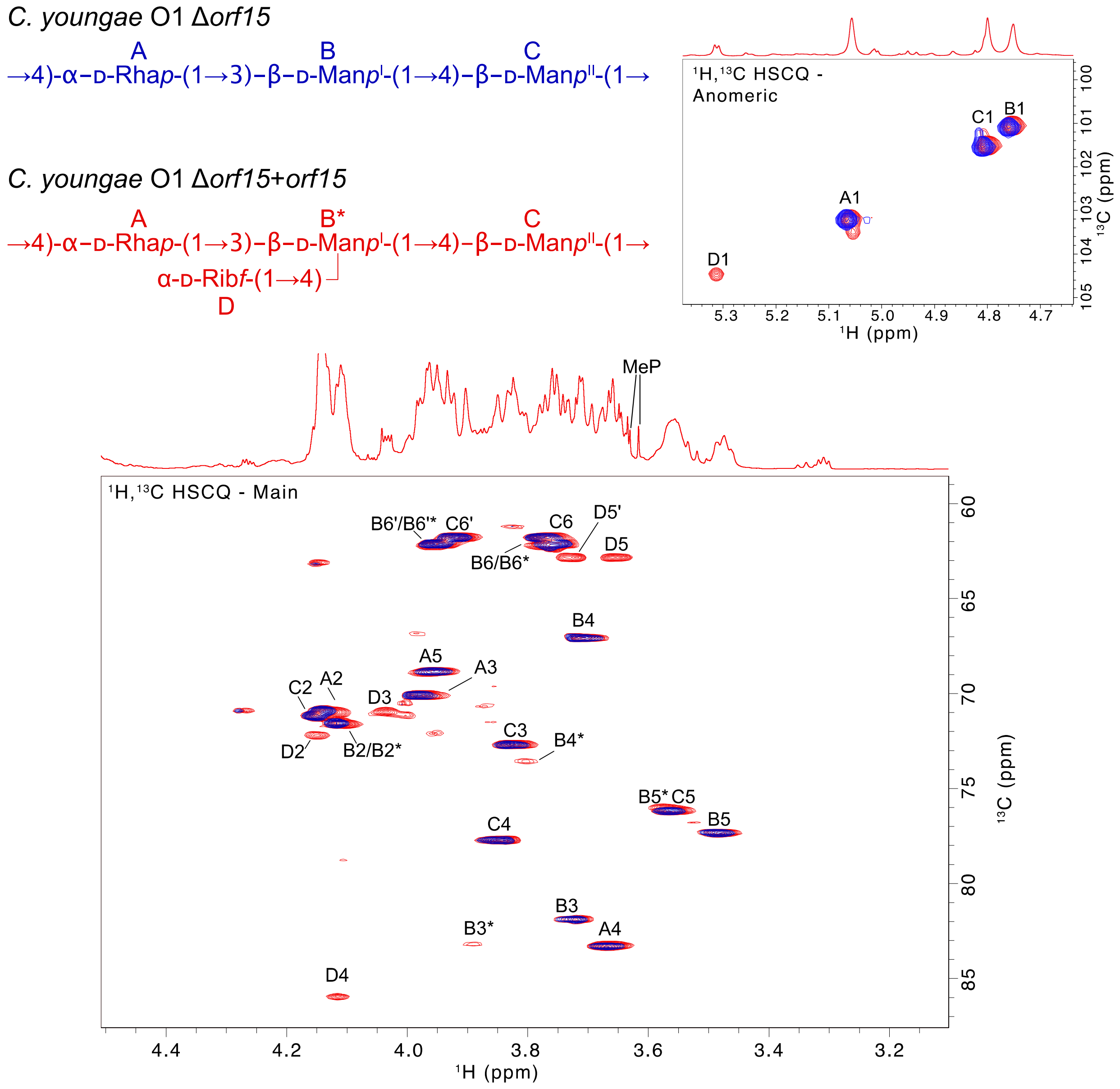

C. youngae O1 Δorf15
A B C
→4)-α-D-Rhap-(1→3)-β-D-Manp[I]-(1→4)-β-D-Manp[II]-(1→
C. youngae O1 Δorf15+orf15
A B* C
→4)-α-D-Rhap-(1→3)-β-D-Manp[I]-(1→4)-β-D-Manp[II]-(1→
α-D-Ribf-(1→4)
D
1H,13C HSCQ - Anomeric
C1
B1
A1
D1
1H (ppm)
13C (ppm)
MeP
1H,13C HSCQ - Main
B6'/B6'*
C6'
C6
D5'
D5
B6/B6*
B4
A5
A3
C2
A2
D3
C3
B4*
D2
B2/B2*
B5*
C5
B5
C4
B3
B3*
A4
D4
1H (ppm)
13C (ppm)

c

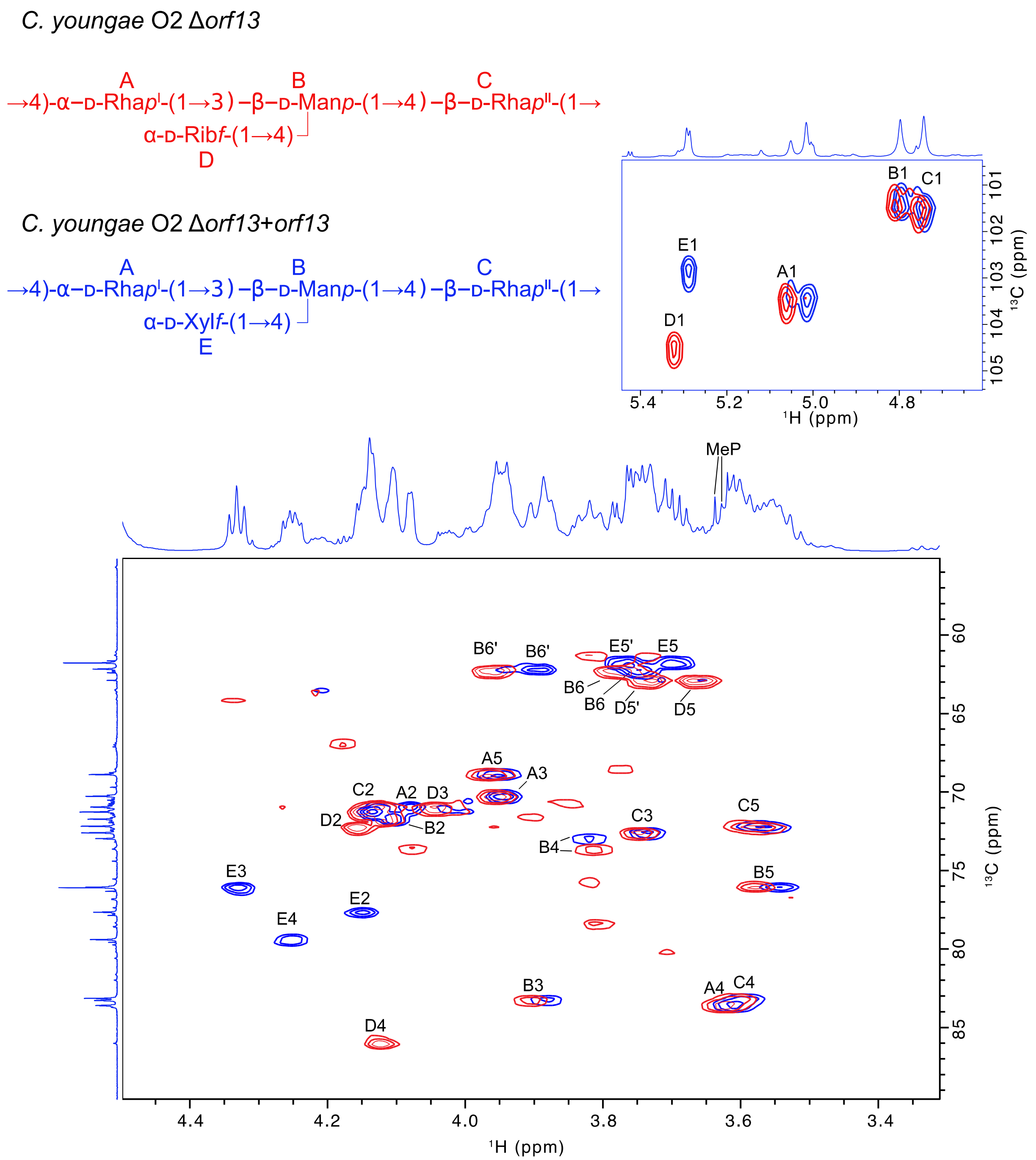

Figure S1: **NMR spectroscopic analysis of OPS from *C. youngae* O1 and O2 mutants and the corresponding mutants complemented with plasmids carrying the relevant genes. a**, overlay of ^{1}H,^{13}C HSQC spectra from *C. youngae* O1 Δ*orf12* (blue) and complement (red). **b**, overlay of ^{1}H,^{13}C HSQC spectra from *C. youngae* O1 Δ*orf15* (blue) and the plasmid-complemented mutant (red). Minor peaks observed in the complemented *orf12* and *orf15* mutants reflect non-stoichiometric modification of Rib*f*

addition reported previously (15, 16), and peak integration comparing the backbone repeat unit and the Rib*f* confirmed the side chain Rib*f* to be attached to 50% of the main chain repeat units. The proposed methylphosphate modification is labelled on each ^{1}H spectrum; this signal appears as a doublet due to splitting by the ^{31}P atom in the phosphate. This is a result of the methyl group neighbouring the NMR active ^{31}P atom, which causes spin coupling and peak splitting into n+1 (two) peaks where n is the number of neighbouring NMR active atoms. **c**, overlay of ^{1}H,^{13}C HSQC spectra from *C. youngae* O2 Δ*orf13* (red) and complement (blue). Minor peaks were observed, consistent with the non-stoichiometric modification (creating unmodified repeating units) as well as incomplete epimerization of Xyl*f* to Rib*f* resulting in a small amount of Rib*f* modified glycan chains in the transformant.

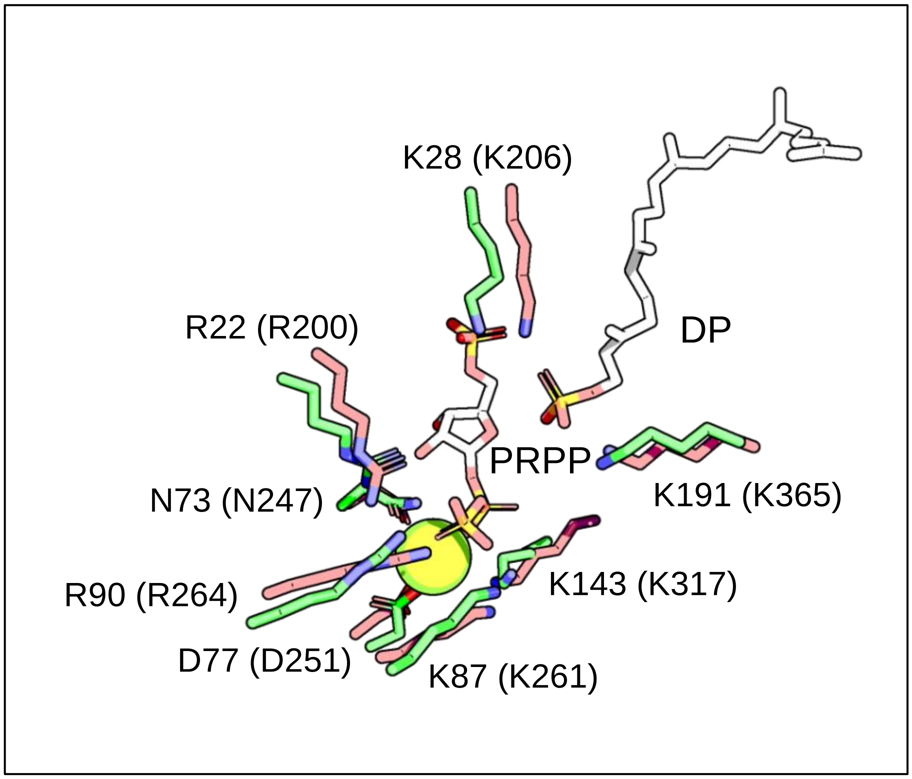

Figure S2: Overlay of the active site of the solved structure of the DPPRS from *Mycobacterium tuberculosis* (Rv3806c) with the AlphaFold model of *C. youngae* O1 PPPRS. Overlay of the AlphaFold model was performed using Dali with the PRPP bound state of Rv3806c (PDB:8j8k). The lipid donor state (PDB:8j8j) was aligned to the PRPP bound state in Pymol to position the decaprenol-phosphate (DP) donor in the active site in a single model with PRPP. Active site residues are shown as green sticks for Rv3806c and the corresponding residues from the ORF15^{C} model are shown has pink sticks with the corresponding residues labelled in brackets. Mg^{++} is shown as a sphere.

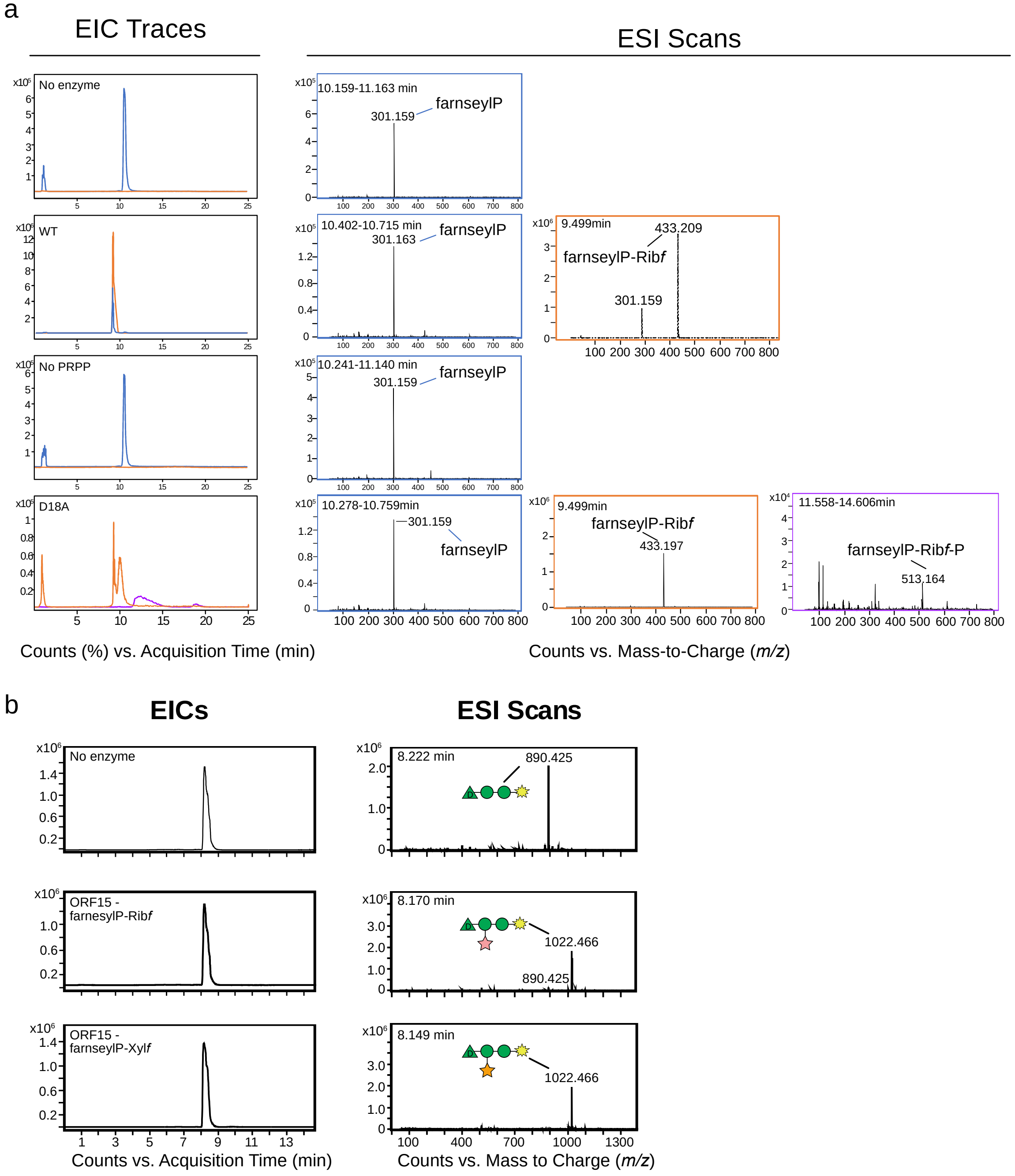

Figure S3: **Mass spectra confirming the identity of ORF15 (a) and ORF12 (b) reaction products**. **a**, overlaid extracted ion chromatograms for farnesyl phosphate (blue; *m/z* 301.16), farnesyl phosphoribose (orange; *m/z* 433.20) and farnesyl phosphoribose-5-phosphate (magenta; *m/z* 514.17) are shown on the left, with corresponding ESI scans shown on the right. **b**, Mass spectrometry spectra confirming the identity of ORF15 reaction products. Extracted ion chromatograms for **3** (*m/z* 890.41) and **3** + Rib*f*/Xyl*f* (*m/z* 1022.46) are shown on the left with corresponding ESI scans shown on the right.

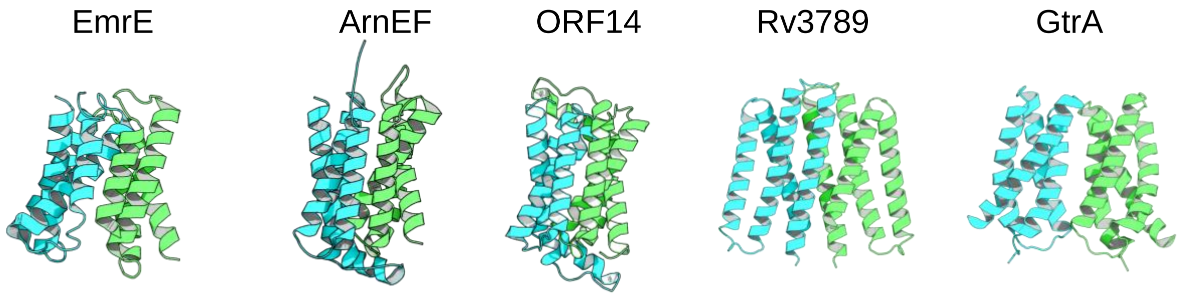

Figure S4: Models of MATE and SMR-family transporters. The models are shown as homo/heterodimers with each chain coloured differently. The structure of EmrE has been solved (7MH6). EmrE adopts a head-to-tail dimerization of subunits, a hallmark of the MATE transporter family. ORF14 (AWU66545.1) is predicted to be a MATE transporter with similarity to ArnEF (Q47377, P76474). AlphaFold modelling of ORF14 and ArnEF predict a similar anti-parallel organization of subunits to the EmrE prototype. AlphaFold models of the mycobacterial decP-Ara*f* transporter (Rv3789 – P9WMS9) and the undP-Glc transporter (GtrA – P77682) adopt an SMR fold, lacking the characteristic antiparallel orientation seen in the MATE family.

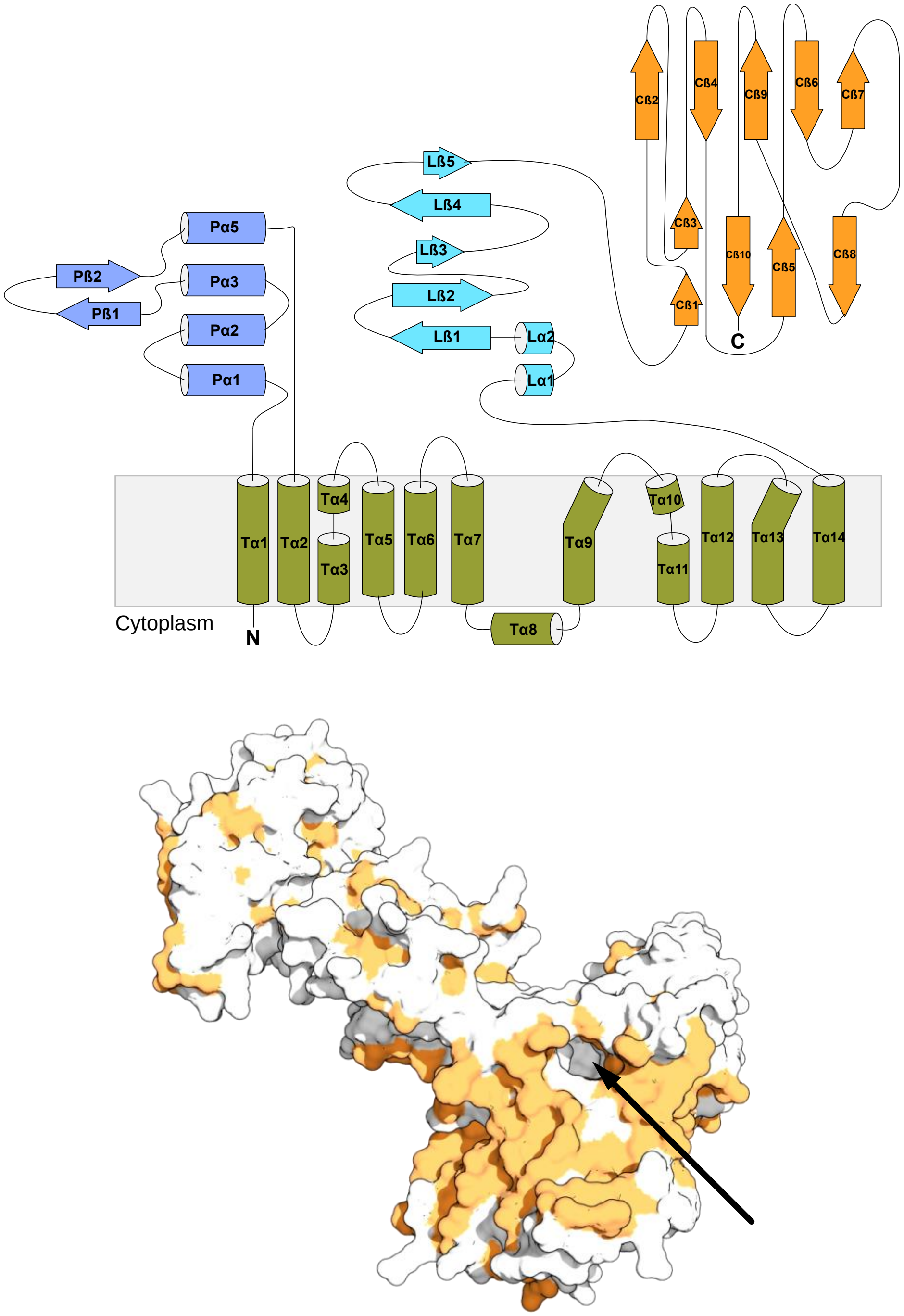

Figure S5: Predicted and structure of ORF12. The *upper* panel shows the predicted domain topology map. The sequence elements are labelled with T (transmembrane helices), P (first periplasmic domain), L (linker region) and C (CBM domain). Domain colours correspond to those used in **Figure 4**. The *lower* panel shows a surface representation of the ORF12 model with hydrophobic residues coloured orange. The region of hydrophobic residues leads to a hole (shown with an arrow) that accesses the proposed active site and could accommodate the lipid tail of the lipid-phosphate linked donor.

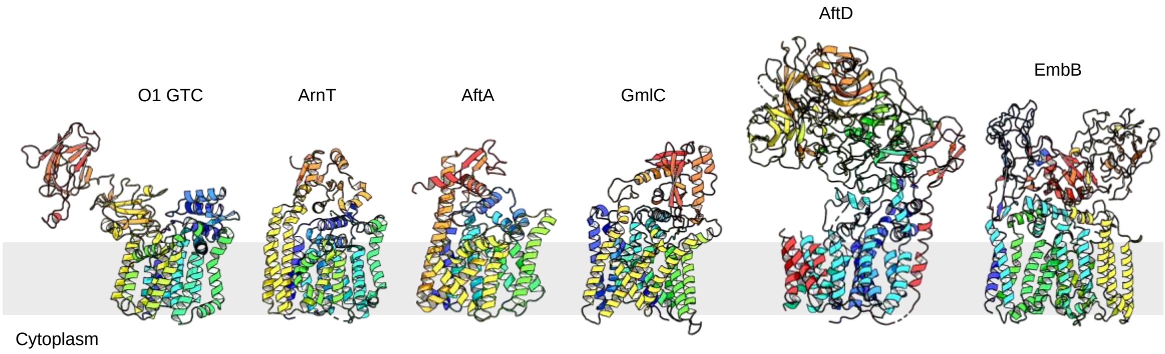

Figure S6: **Structural comparison of ORF12 and selected GT-C enzymes.** An AlphaFold model of ORF12 (O1 GTC) compared to crystal structures of GT-C enzymes ArnT (5F15), AftA (8IF8), AftD (6WBY), EmbB (7BWR) and AlphaFold model of GmlC (AVA30552.1). Structures are coloured as a gradient from green (N-terminus) to red (C-terminus).

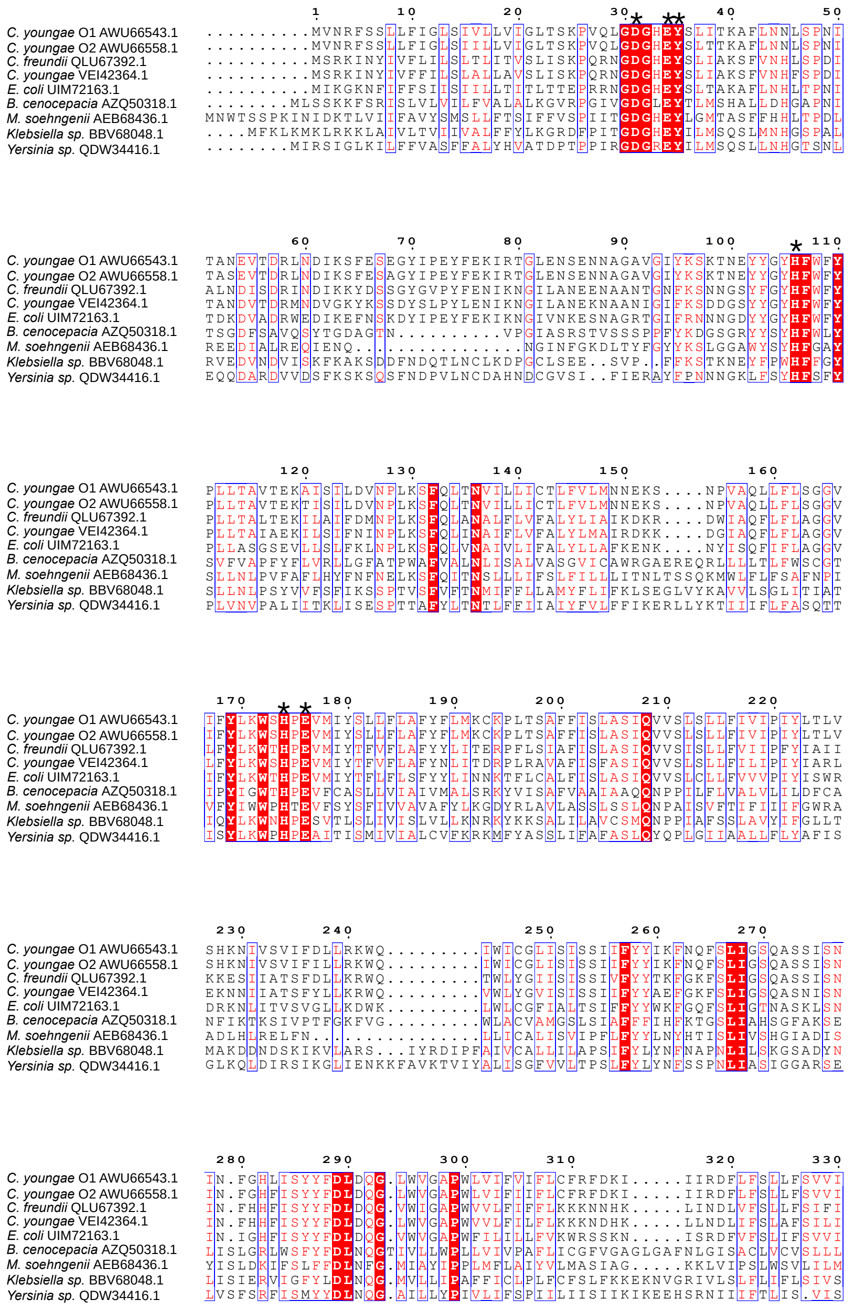
C. youngae O1 AWU66543.1
C. youngae O2 AWU66558.1
C. freundii QLU67392.1
C. youngae VEI42364.1
E. coli UIM72163.1
B. cenocepacia AZQ50318.1
M. soehngenii AEB68436.1
Klebsiella sp. BBV68048.1
Yersinia sp. QDW34416.1

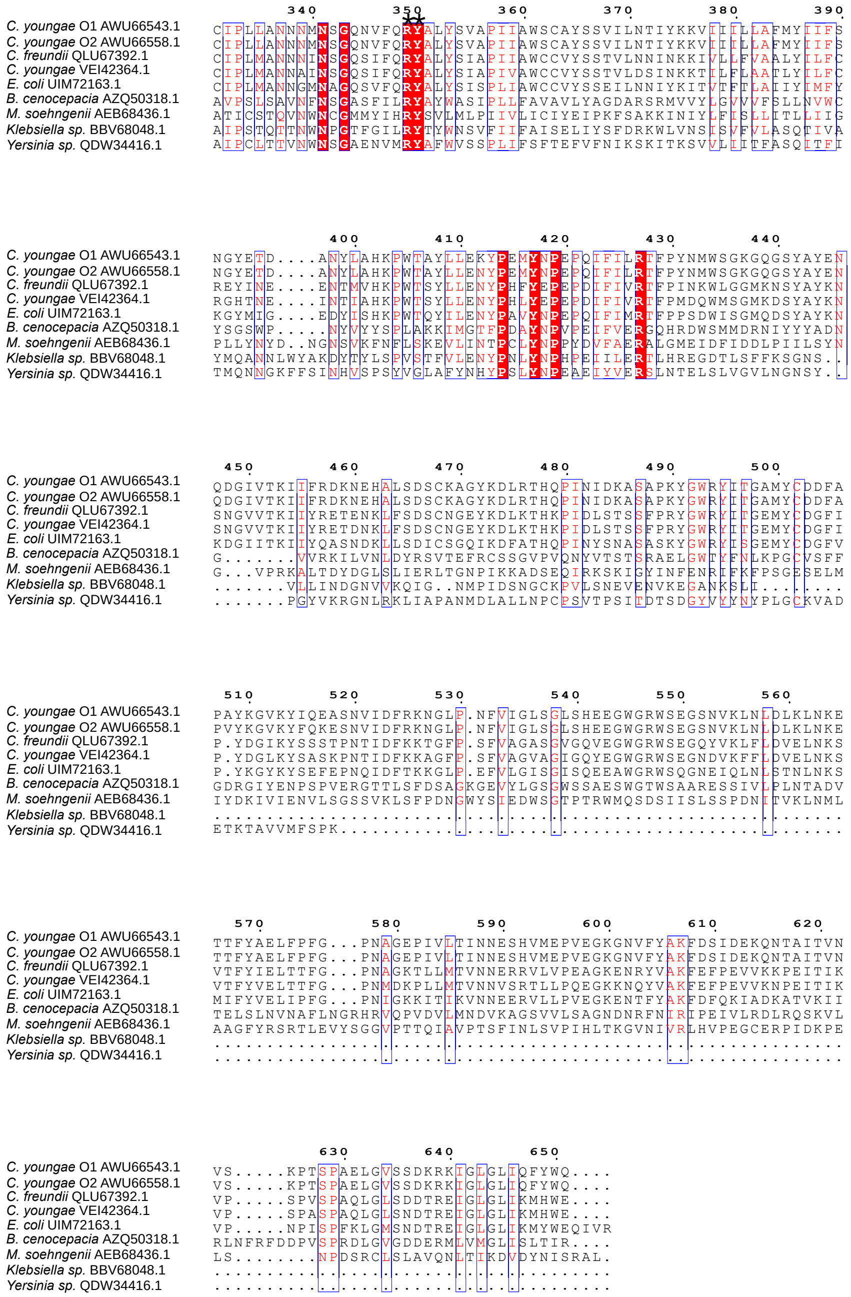

Figure S7: **Multiple sequence alignment of ORF12 and its orthologs.** A tBLASTn search was conducted using ORF12 and orthologs found in candidate three-component modification systems were retained. The alignment includes *C. youngae* O1 (AWU66543.1), *C. youngae* O2 (AWU66558.1), *C. freundii* RHBSTW-00355 (QLU67392.1), *C. youngae* NCTC13709 (VEI42364.1), *E. coli* CU37RT-2 (UIM72163.1), *B. cenocepacia* YG-3 (AZQ50318.1), *M. soehngenii* GP-6 (AEB68436.1), *Klebsiella sp.* STW0522-44 (BBV68048.1), *Yersinia sp*. KBS0713 (QDW34416.1). Conserved residues marked with an asterisk were targeted for alanine replacement. This multiple

sequence alignment was also used for the construction of the CONSURF map used in **Figure 4.**

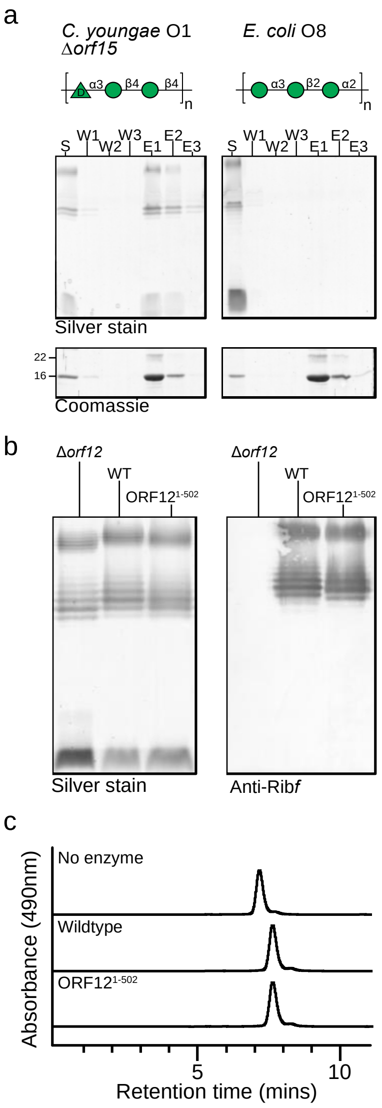

Figure S8: The CBM in ORF12 binds the unmodified O1 OPS backbone but is not essential for *in vitro* or *in vivo* activity. a, to test the ability of the CBM domain to bind O1 OPS, purified ORF12$^{503\text{-}652}$-His$_6$ was incubated with LPS from *C. youngae* O1 Δ*orf15*, or control LPS from *E. coli* O8. The hexahistidine-tagged CBM was then recovered using magnetic nickel beads, and the fractions (S, reaction sample, W, wash; and E, elution with 250mM imidazole) were assessed by silver staining (after proteinase K treatment to highlight LPS) and by Coomassie blue to show protein. *C. youngae* O1 Δ*orf12* LPS co-eluted with the CBM, while the control LPS was not retained by eluted CBM. **b**, the requirement for the CBM *in vivo* was assessed by the ability of plasmid-encoded ORF12$^{1\text{-}502}$ (lacking the CBM) to retore the Rib*f* side chain epitope in the*Δorf12* mutant. Whole-cell lysates were separated by PAGE and analyzed with silver staining or western immunoblotting with antiserum specific for the Rib*f* side chain **c,** the activity of ORF12$^{1\text{-}502}$ was also tested *in vitro.* Reaction products were conducted using synthetic farnesyl-phosphoribose (**2**) and BODIPY-tagged O1 repeat unit mimic (**3**), and separated using HPLC with a GlycoSep N column and normal phase separation.

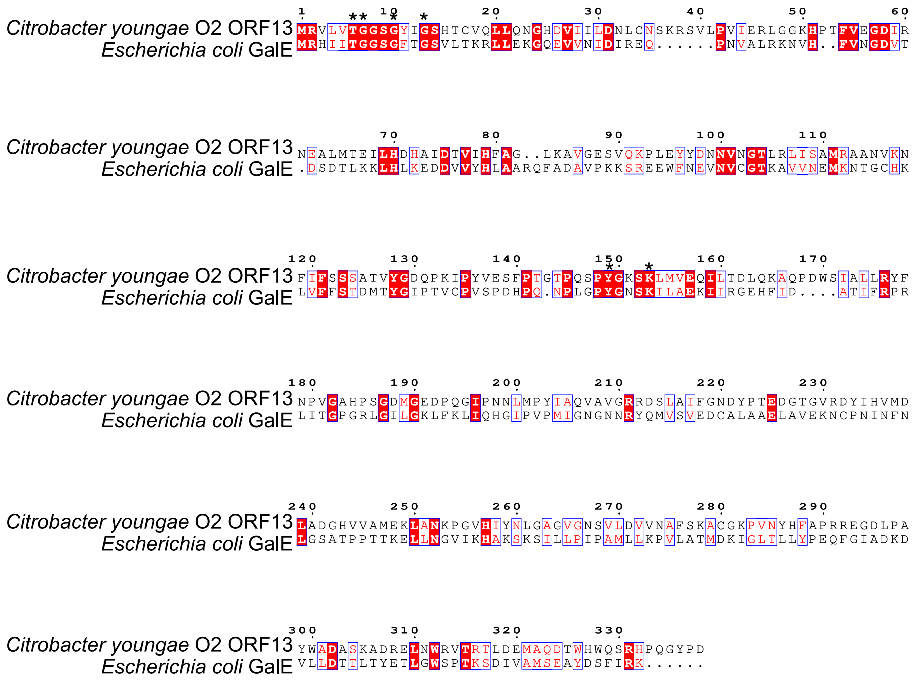

Figure S9: **Multiple sequence alignment of *C. youngae* O2 ORF13 with UDP-galactose-4-epimerase GalE from *E. coli* (P09147).** Asterisks mark the TGXXGXGG and YXXXK motifs used by SDR epimerases to bind NADH and catalyze epimerization respectively (17).

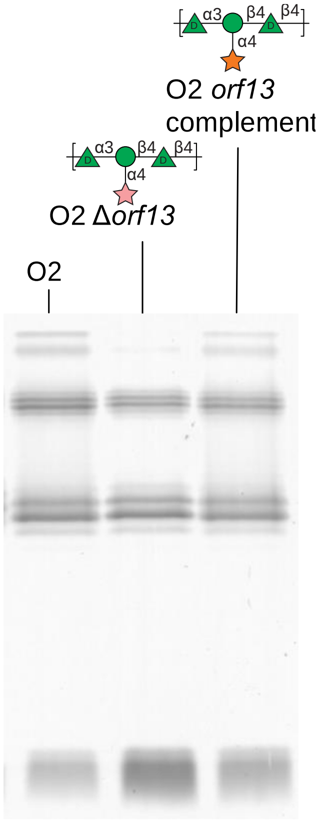

Figure S10: **Silver-stained PAGE of LPS in whole-cell lysates of *C. youngae* O2 Δ*orf13* and the mutant complemented using a plasmid carrying *orf13*.** The observed LPS phenotype of identical length is consistent with the maintenance of the side-chain modification, consistent with the promiscuity of ORF12.

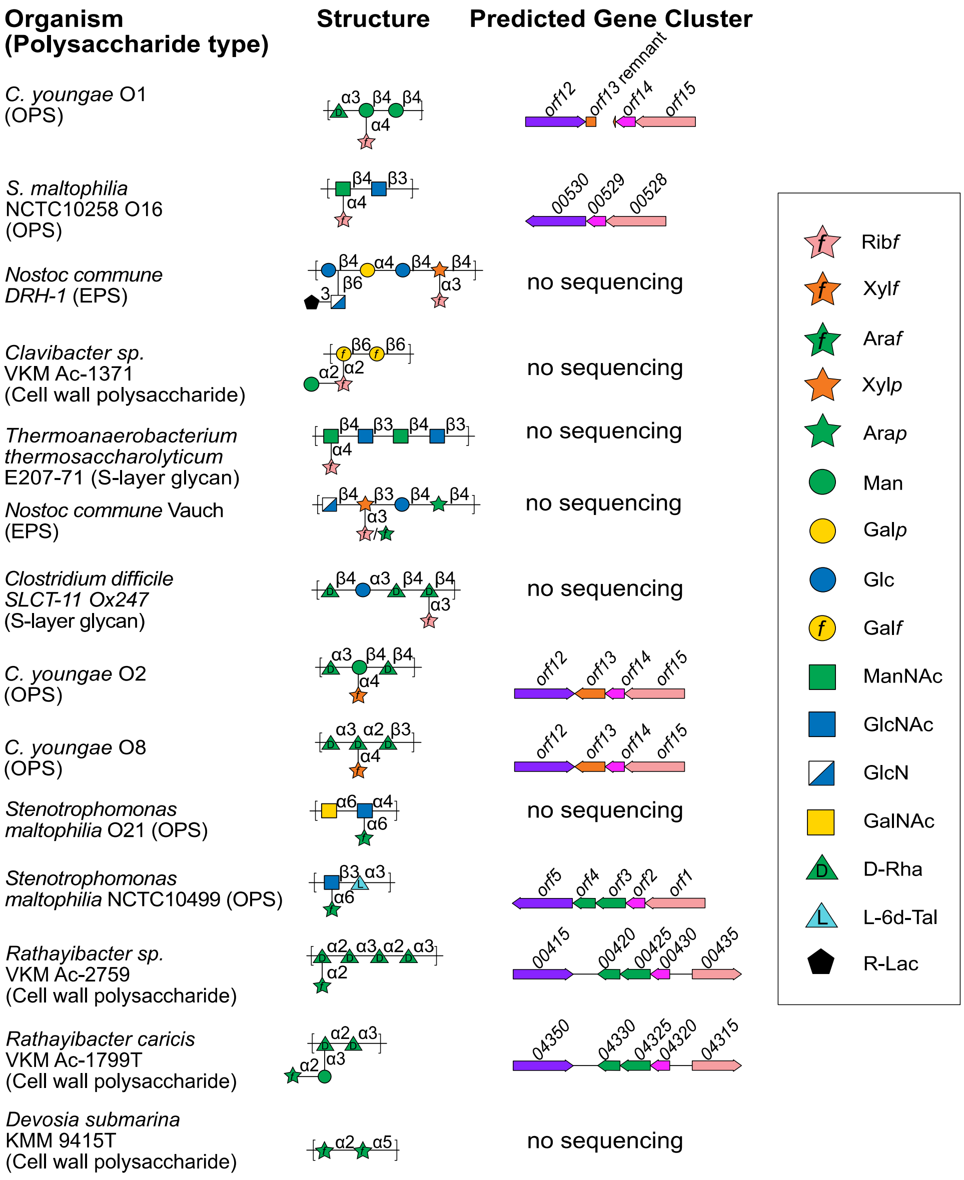

Figure S11: Additional microbial glycans with α-pentofuranose side-chain modifications. The Carbohydrate Structure Database was queried for polysaccharide structures containing α-pentofuranoses, and only some structures contained corresponding sequence for the locus from Genbank. In addition to the *C. youngae* O1 (MH325883.1) prototype, the search identified *S. maltophilia* O16 (NCTC10258) (LS483377.1), *C. youngae* O2 (MH325884.1), *C. youngae* O8 (MH325890.1), *S. maltophilia* NCTC10499 (CABEER010000007.1), *Rathayibacter sp.* VKM Ac-2759 (NZ_CP047176.1), and *Rathayibacter caricis.* VKM Ac-1799T (NZ_PZPL01000001.1). The polysaccharide repeat-unit structures are shown and the genes encoding enzyme homologs are coordinated by color (PPPRS in salmon, GT-C in purple, transporter in pink and Xyl*f*/Ara*f* epimerases in orange and green respectively). A genomic sequence was available for *Agrobacterium tumefaciens* (since renamed *Rhizobium radiobacter*) DSM 30205=NCTC 13543 (GCA_900455815), but orthologs of the three-component

system proteins were not identifiable. OPS and EPS designate O-polysaccharide and exopolysaccharide, respectively. The glycan from *Devosia submarinarum* was a component of extracted “cell wall polysaccharides” (18) and is structurally related to mycobacterial arabinan and *P. aeruginosa O*-glycans, so may share similar extracytoplasmic biosynthesis. Sugars are represented using the symbol nomenclature for glycans (SNFG) (19). Abbreviated sugars are as follows: Rib*f* (ribofuranose), Xyl*f* (xylofuranose), Ara*f* (arabinofuranose), Xyl*p* (xylopyranose), Ara*p* (arabinopyranose), Man (mannose), Gal*p* (galactopyranose), Glc (glucose), Gal*f* (galactofuranose), ManNAc (*N*-acetylmannosamine), GlcNAc (*N*-acetylglucosamine), GlcN (glucosamine), GalNAc (*N*-acetylgalactosamine), ᴅ-Rha (ᴅ-rhamnose), ʟ-6d-Tal (6-deoxy-talose), R-Lac (3-O-[(R)-1-carboxyethyl]-ᴅ-glucuronic acid; nosturonic acid)

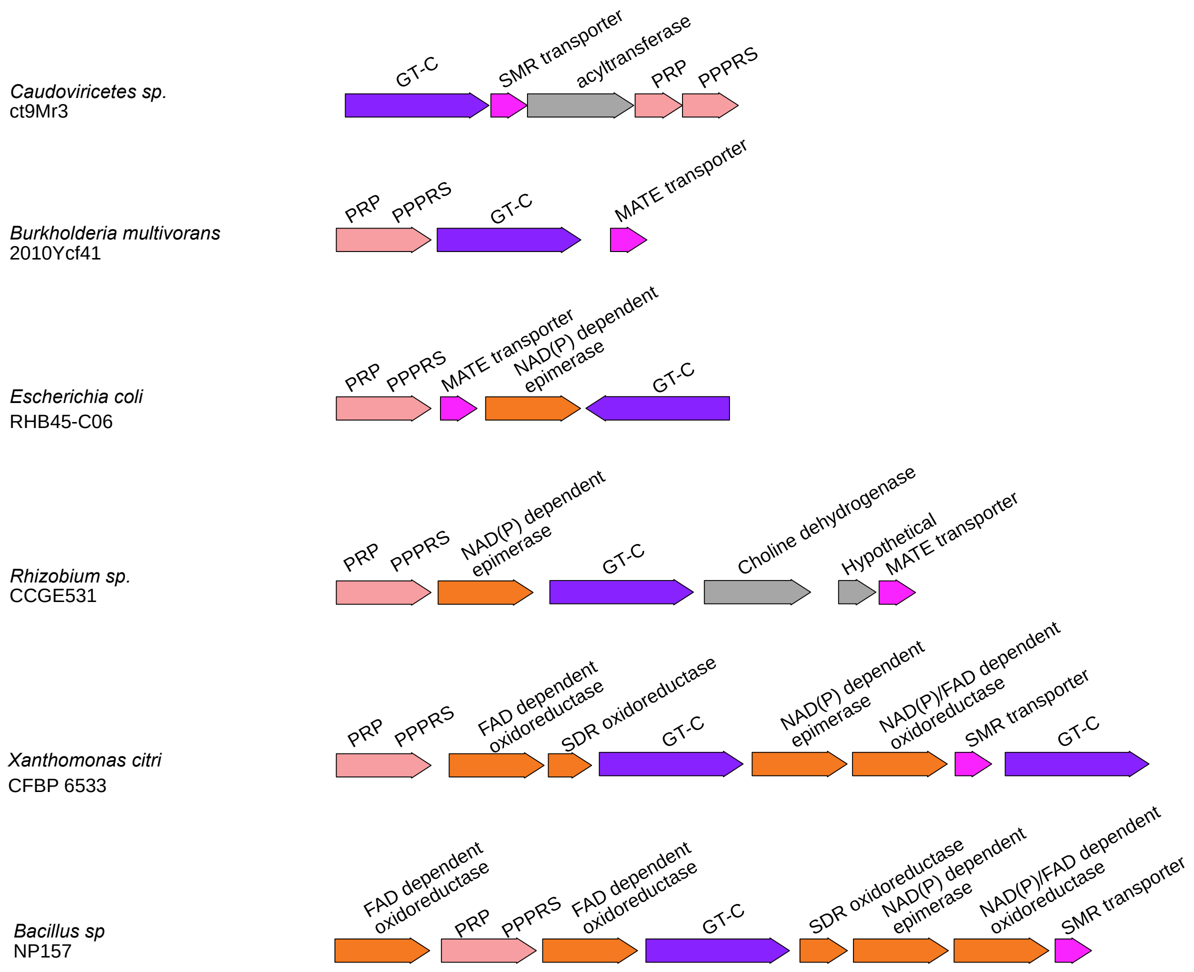

Figure S12: Genetic loci encoding candidate three-component α-pentofuranose side chain addition systems. These are present in clusters directing the synthesis of glycans with unknown structure. *C. youngae* O1 ORF15 was used as a seed in a tBLASTn search. Sequences flanking and nearby *orf15* homologs were scanned for genes predicted to encode a transporter and a GT-C enzyme. Notably most GT-C genes were labelled as hypothetical proteins, but the AlphaFold models of close homologs found in Uniprot allowed confident assignments of GT-C gene products. In one example (in *Caudoviricetes*) the PRP domain is separated from the PPPRS. The transporters in the systems varied, containing either a MATE family transporter (like *C. youngae* O1) or an SMR family transporter. Finally, several more elaborate systems were identified which contained multiple different epimerases and GT-C enzymes, suggesting these systems may be capable of more elaborate side-chain additions. Accessions for the identified systems are as follows: *Caudoviricetes sp.* ct9Mr3 (BK026208.1), *Burkholderia multivorans* 2010Ycf41 (CP090754.1), *Escherichia coli* RHB45-C06 (CP099034.1), *Rhizobium sp.* CCGE531 (CP032687.1), *Xanthomonas citri* CFBP 6533 (CP110301.1), *Bacillus sp.* strain NP157 (CP076546.1).

Table S1: Predicted function of proteins encoded by *C. youngae* O1 OPS cluster based on similarity to characterized enzymes.

***C. youngae* O1 ORF**	**Characterized Homolog (Accession #)**	**Protein identity/similarity**	**CDD Prediction/ Proposed Function**
ORF1	*E. coli* O9 ManC/ BAA07745.1	77%/87%	Mannose-1-phosphate guanylyltransferase mannose-6-phosphate isomerase / *GDP-mannose biosynthesis*
ORF2	*E. coli* O9 ManB/ BAA07746.1	78%/88%	Phosphomannomutase/ *GDP-mannose biosynthesis*
ORF3	*E. coli* O9 Wzm/ BAA07747.1	51%/73%	ABC-type polysaccharide export permease/ *O-antigen flippase transmembrane domain*
ORF4	*E. coli* O9 Wzt/ BAA07748.1	58%/76%	ABC-type polysaccharide phosphate transport system- ATPase component, Wzt-C/ *O-antigen flippase nucleotide binding domain*
ORF5	*E. coli* O9 WbdD/ BAA07749.1	39%/56%	SAM-dependent methyltransferase, kinase/ *O-antigen terminator*
ORF7	*E. coli* O99 putative polymerase/ UIM73163.1	59%/74%	GT-4, GT-1, GT-4/ *O-antigen polymerase*
ORF8	*E. coli* O9a Gmd/ CP089930.1	54%/71%	GDP-mannose 4,6-dehydratase/ *GDP-rhamnose biosynthesis*
ORF9	*E. coli* O9a Rmd/ ACV53840.1	84%/92%	NAD(P)(+) binding protein/ *GDP-rhamnose biosynthesis*
ORF10	*E. coli* O9 WbdB/ BAA07751.1	49%/67%	GT-4/ *Initiating mannosyltransferase*
ORF11	*E. coli* O9 WbdC/ BAA07752.1	55%/71%	GT-4/ *Initiating mannosyltransferase*

ORF12	No significantly similar characterized proteins	N/A	EpsG glycosyltransferase family, alkaline phosphatase like superfamily/ *GT-C glycosyltransferase*
ORF14	No significantly similar characterized proteins	N/A	MATE transporter/ *UndP-ribofuranose flippase*
ORF15	*M. tuberculosis* Rv3806c/ CCP46635.1	31%/54% (across 61% of ORF15)	UbiA family prenyltransferase/ *UndP-ribofuranose donor synthase*

[1]**Table S2**: ^{1}H and ^{13}C NMR chemical shifts for *C. youngae* mutants and complements

O1 Δ*orf15*

Sugar Residue		Chemical shift (ppm)					
		H-1<br>C-1	*H-2*<br>C-2	*H-3*<br>C-3	*H-4*<br>C-4	*H-5 (5a,b)*<br>C-5	*H-6 (6a,b)*<br>C-6
Repeat Unit							
→4)-α-D-Rha*p*-(1→	**A**	*5.05*	*4.13*	*3.97*	*3.65*	*3.94*	*1.31*
		103.3	71.0	70.2	83.4	69.0	17.9
→3)-β-D-Man*p*^{I}-(1→	**B**	*4.75*	*4.10*	*3.71*	*3.70*	*3.47*	*3.74, 3.94*
		101.2	71.7	82.0	67.2	77.5	62.2
→4)-β-D-Man*p*^{II}-(1→	**C**	*4.79*	*4.14*	*3.82*	*3.83*	*3.55*	*3.76, 3.90*
		101.6	71.3	72.8	77.8	76.3	61.8

O1 *orf15* complement

Sugar Residue		Chemical shift (ppm)					
		H-1<br>C-1	*H-2*<br>C-2	*H-3*<br>C-3	*H-4*<br>C-4	*H-5 (5a,b)*<br>C-5	*H-6 (6a,b)*<br>C-6
Repeat Unit							
→4)-α-D-Rha*p*-(1→	**A**	*5.05*	*4.12*	*3.97*	*3.65*	*3.96*	*1.32*
		103.6	71.0	70.2	83.3	69.0	17.9
→3,4)-β-D-Man*p*^{I}-(1→	**B**	*4.75*	*4.10*	*3.89*	3.80	3.57	*3.76, 3.94*
		101.0	71.7	83.3	73.7	76.1	62.2
→4)-β-D-Man*p*^{II}-(1→	**C**	*4.80*	*4.14*	*3.82*	*3.84*	*3.55*	*3.76, 3.90*
		101.6	71.3	72.8	77.4	76.3	61.9
-α-D-Rib*f*-(1→	**D**	*5.31*	*4.15*	4.03	4.11	*3.65, 3.71*	
		104.6	72.4	71.0	86.1	63.0	

O1 Δ*orf12*

Sugar Residue		Chemical shift (ppm)					
		H-1<br>C-1	*H-2*<br>C-2	*H-3*<br>C-3	*H-4*<br>C-4	*H-5 (5a,b)*<br>C-5	*H-6 (6a,b)*<br>C-6
Repeat Unit							
→4)-α-D-Rha*p*-(1→	**A**	*5.07*	*4.14*	*3.98*	*3.67*	*3.95*	*1.33*
		103.2	70.9	70.1	83.3	68.8	17.7
→3)-β-D-Man*p*^{I}-(1→	**B**	*4.76*	*4.12*	*3.72*	*3.72*	*3.48*	*3.75, 3.95*
		101.1	71.5	81.9	67.0	77.3	62.0
→4)-β-D-Man*p*^{II}-(1→	**C**	*4.81*	*4.15*	*3.83*	*3.85*	*3.56*	*3.77, 3.92*
		101.5	71.1	72.7	77.7	76.2	61.7

O1 *orf12* complement

Sugar Residue		Chemical shift (ppm)					
		H-1	*H-2*	*H-3*	*H-4*	*H-5 (5a,b)*	*H-6 (6a,b)*
		C-1	C-2	C-3	C-4	C-5	C-6
Repeat Unit							
→4)-α-D-Rha*p*-(1→	**A**	5.06	4.13	3.97	3.66	3.95	1.32
		103.2	70.9	70.1	83.2	68.8	17.8
→3,4)-β-D-Man*p*^{I}-(1→	**B**	4.75	4.11	3.89	3.80	3.58	3.78, 3.95
		101.0	71.6	83.2	73.5	76.0	62.2
→4)-β-D-Man*p*^{II}-(1→	**C**	4.80	4.14	3.83	3.84	3.55	3.76, 3.91
		101.4	71.1	72.7	77.7	76.5	61.8
-α-D-Rib*f*-(1→	**D**	5.31	4.15	4.04	4.11	3.65, 3.72	
		104.4	72.1	70.9	85.9	62.8	

O2 Δ*orf13*

Sugar Residue		Chemical shift (ppm)					
		H-1	*H-2*	*H-3*	*H-4*	*H-5 (5a,b)*	*H-6 (6a,b)*
		C-1	C-2	C-3	C-4	C-5	C-6
Repeat Unit							
→4)-α-D-Rha*p*-(1→	**A**	5.06	4.12	3.95	3.63	3.97	1.31
		103.6	71.0	70.3	83.7	68.9	17.7
→3,4)-β-D-Man*p*-(1→	**B**	4.81	4.11	3.91	3.81	3.58	3.78, 3.96
		101.3	71.8	83.2	73.7	76.0	62.3
→4)-β-D-Rha*p*-(1→	**C**	4.76	4.15	3.75	3.60	3.58	1.36
		101.5	71.3	72.6	83.2	72.2	18.0
-α-D-Rib*f*-(1→	**D**	5.32	4.16	4.04	4.12	3.66, 3.73	
		104.5	72.2	71.0	86.1	63.0	

O2 *orf13* complement

Sugar Residue		Chemical shift (ppm)					
		H-1	*H-2*	*H-3*	*H-4*	*H-5 (5a,b)*	*H-6 (6a,b)*
		C-1	C-2	C-3	C-4	C-5	C-6
Repeat Unit							
→4)-α-D-Rha*p*-(1→	**A**	5,01	4.08	3.95	3.61	3.94	1.30
		103.6	71.0	69.0	83.7	70.3	17.8
→3,4)-β-D-Man*p*-(1→	**B**	4.80	4.10	3.88	3.82	3.55	3.75, 3.90
		101.4	71.8	83.2	73.1	76.2	61.8
→4)-β-D-Rha*p*-(1→	**C**	4.74	4.14	3.74	3.59	3.56	1.35
		101.6	71.4	72.7	83.2	72.3	18.0
-α-D-Xyl*f*-(1→	**D**	5.29	4.15	4.33	4.26	3.70, 3.76	
		103.0	77.7	76.2	79.5	61.9	

[1]The signal for the putative methylphosphate group is at δ_H 3.61, δ_C 54.24

Table S3: Plasmids and bacterial strains

Strain or plasmid	Description	Reference
**Bacterial strains**		
E. coli DH5α	K-12 F⁻ φ80*lacZ* çM15 Δ(*lacZYA-argF*) U169 *deoR recA1 endA1 hsdR17* (r_K^-, m_K^+) *gal*⁻ *phoA supE44 thi*⁻ *gyrA96 relA1*	(20, 21)
E. coli Top10	F⁻, *mcrA*, Δ(*mrr-hsdRMS-mcrBC*), φ80, *lacZ*Δ*M15*, Δ*lacX74*, *deoR*, *nupG*, *recA1*, *araD139*, Δ(*ara-leu*)7697, *galU*, *galK*, *repsL*(Str^R), *endA1*	Invitrogen
C. youngae PCM 1492	Serotype O1	Polish Collection of Microorganisms
CWG1508	*C. youngae* O1 with *orf15* replaced with Kan^r cassette from pKD4	This study
CWG1509	*C. youngae* O1 with *orf12* replaced with Kan^r cassette from pKD4	This study
C. youngae PCM 1507	Serotype O2	Polish Collection of Microorganisms
CWG1510	*C. youngae* O2 with *orf13* replaced with Kan^r cassette from pKD4	This study
**Plasmids**		
pBAD18-Cm	Cloning vector with ʟ-arabinose-inducible promoter; Cm^r	(21)
pKD46	Temperature sensitive, encodes lambda Red genes (*exo*, *bet*, *gam*); arabinose-inducible promoter; Ap^r	(22)
pKD4	Source of Kanamycin resistance cassette; Kan^r	(22)
pWQ1081	pBAD18-Cm derivative encoding *C. youngae* O1 ORF15-His_6	This study

pWQ1082	pBAD18-Cm derivative encoding *C. youngae* O1 ORF12-His_6	This study
pWQ1083	pBAD18-Cm derivative encoding *C. youngae* O2 ORF13-His_6	This study
pWQ1084	pBAD18-Cm derivative encoding *C. youngae* O1 $ORF15^{D18A}$-His_6	This study
pWQ1085	pBAD18-Cm derivative encoding *C. youngae* O1 $ORF15^{N247A}$-His_6	This study
pWQ1086	pBAD18-Cm derivative encoding *C. youngae* O1 $ORF15^{D248A}$-His_6	This study
pWQ1087	pBAD18-Cm derivative encoding *C. youngae* O1 $ORF15^{D251A}$-His_6	This study
pWQ1088	pBAD18-Cm derivative encoding *C. youngae* O1 $ORF15^{D255A}$-His_6	This study
pWQ1089	pBAD18-Cm derivative encoding *C. youngae* O1 $ORF15^{R66A}$-His_6	This study
pWQ1090	pBAD18-Cm derivative encoding *C. youngae* O1 $ORF15^{E369A}$-His_6	This study
pWQ1091	pBAD18-Cm derivative encoding *C. youngae* O1 $ORF12^{D31A}$-His_6	This study
pWQ1092	pBAD18-Cm derivative encoding *C. youngae* O1 $ORF12^{E34A}$-His_6	This study
pWQ1093	pBAD18-Cm derivative encoding *C. youngae* O1 $ORF12^{Y35A}$-His_6	This study
pWQ1094	pBAD18-Cm derivative encoding *C. youngae* O1 $ORF12^{H106A}$-His_6	This study
pWQ1095	pBAD18-Cm derivative encoding *C. youngae* O1 $ORF12^{H174A}$-His_6	This study
pWQ1096	pBAD18-Cm derivative encoding *C. youngae* O1 $ORF12^{E176A}$-His_6	This study
pWQ1097	pBAD18-Cm derivative encoding *C. youngae* O1 $ORF12^{R349A}$-His_6	This study
pWQ1098	pBAD18-Cm derivative encoding *C. youngae* O1 $ORF12^{1\text{-}502}$-His_6	This study
pWQ1100	pBAD18-Cm derivative encoding *C. youngae* O1 $ORF12^{503\text{-}651}$-His_6	This study

Table S4: Oligonucleotide primers sequences[abc]

Plasmid Generated	Primer Sequence Forward/ Reverse	Description
	5′-tacttctcgattgagaatcgatgtagattctgcttagccaaggaatgtaaTGTGTAGGCTGGAGCTGCTTC<br><br>5′-cgcttgttaacgctagtaatagaacgatcaaactcatttttcaccacgacCATATGAATATCCTCCTTAG	Lamba red replace *orf15* with Kan
	5′-atcattcataactattctcataaaatattttaaatttctaaggttgaataTGTGTAGGCTGGAGCTGCTTC<br><br>5′-ctgctagatcctatcctctaatgatttgtcagaattatatttaactaactCATATGAATATCCTCCTTAG	Lambda red replace *orf12* with KanR
	5′-ggcttgtctctaatttgcgtcggaattgtatttatctctaggagtgcataaTGTGTAGGCTGGAGCTGCTTC<br><br>5′-gaaaaatcattagaggataggatctagcagatcctatcctttttttatgcCATATGAATATCCTCCTTAG	Lambda red replace *orf13* with KanR
pWQ1081	5′-GATCGCTAGCAGGAGGAATTCACCatg**CATCACCATCACCATCAC**tccaccaagccatttgatgc<br><br>5′-GATCAAGCTTttatagataacttgctatcataaaaccagcg	*C. youngae* O1 ORF15-His_6
pWQ1082	5′-GATCGCTAGCAGGAGGAATTCACCatggtcaataggtttagttctctattgtttattg<br><br>5′-GATCAAGCTTtta**GTGATGGTGATGGTGATG**ctgccaataaaactgaatcaatcccag	*C. youngae* O1 ORF12-His_6
pWQ1083	5′-<br>GATCGCTAGCAGGAGGAATTCACCatg**CATCACCATCACCATCAC**cgtcatattattactggcggaagtg<br><br>5′-GATCAAGCTTttactttcttatgaatgaatcgtatgcttcac	*C. youngae* O2 ORF13-His_6
pWQ1084	5′-CTTGTTGTTGcTTTAGACGGC<br><br>5′-AGGCACATTTATGTCGATAG	*C. youngae* O1 $ORF15^{D18A}$-His_6
pWQ1085	5′-CTATATTATTgcCGATCTAGCAGACCTTG<br><br>5′-GTTGCGGAGGCAATTAAG	*C. youngae* O1 $ORF15^{N247A}$-His_6
pWQ1086	5′-ATTATTAACGcTCTAGCAGACCTTG<br><br>5′-ATAGGTTGCGGAGGCAAT	*C. youngae* O1 $ORF15^{D248A}$-His_6
pWQ1087	5′-GTTAATAATATAGGTTGCGG<br><br>5′-CTTGAAGCCGcTCGTAGACATAAG	*C. youngae* O1 $ORF15^{D251A}$-His_6
pWQ1088	5′-CTTGAAGCCGcTCGTAGACATAAG<br><br>5′-GTCTGCTAGATCGTTAATAATATAG	*C. youngae* O1 $ORF15^{D255A}$-His_6
pWQ1089	5′-TCTGGCAAAAgcGCATGTTGAGATTC<br><br>5′-GCCAGTGAGAAAAATAGC	*C. youngae* O1 $ORF15^{R366A}$-His_6
pWQ1090	5′-CGGCATGTTGcGATTCTCAAATC<br><br>5′-TTTTGCCAGAGCCAGTGA	*C. youngae* O1 $ORF15^{E369A}$-His_6
pWQ1091	5′-CAACTTGGCGcTGGACATGAG<br><br>5′-TACTGGCTTACTTGTTAATC	*C. youngae* O1 $ORF12^{D31A}$-His_6
pWQ1092	5′-GATGGACATGcGTATTCACTCATAAC	*C. youngae* O1

	5′-GCCAAGTTGTACTGGCTT	$ORF12^{E34A}$-His_6
pWQ1093	5′-TGGACATGAGgcTTCACTCATAACAAAAGCATTTTTAAATAATC<br><br>5′-TCGCCAAGTTGTACTGGC	*C. youngae* O1 $ORF12^{Y35A}$-His_6
pWQ1094	5′-CTATGGATACgcCTTTTGGTTCTATCC<br><br>5′-TATTCATTAGTTTTAGATTTATATATACC	*C. youngae* O1 $ORF12^{H106A}$-His_6
pWQ1095	5′-TAAATGGTCGgcTCCAGAAGTAATGATATATTC<br><br>5′-AGATAGAATATGACTCCAC	*C. youngae* O1 $ORF12^{H174A}$-His_6
pWQ1096	5′-TCGCATCCAGctGTAATGATATATTC<br><br>5′-CCATTTAAGATAGAATATGACTC	*C. youngae* O1 $ORF12^{E176A}$-His_6
pWQ1097	5′-TGTCTTTCAAgcATATGCCTTGTATTCTGTTG<br><br>5′-TTCTGCCCTGAGTTCATATTG	*C. youngae* O1 $ORF12^{R349A}$-His_6
pWQ1098	5′-GATCGCTAGCAGGAGGAATTCACCatggtcaataggtttagttctctattgtttattg<br><br>5′-GATCAAGCTTtta**GTGATGGTGATGGTGATG**gcaatacatcgcccctgtaatatag	*C. youngae* O1 $ORF12^{1-502}$-His_6
pWQ1100	5′-GATCGCTAGCAGGAGGAATTCACCatggacgattttgctccagcgtac<br><br>5′-GATCAAGCTTtta**GTGATGGTGATGGTGATG**ctgccaataaaactgaatcaatcccag	*C. youngae* O1 $ORF12^{503-651}$-His_6

[a]restriction endonuclease cut sites are underlined
[b]DNA sequence encoding protein tags are bolded
[c]Mutations for alanine variants are in lower case

Supplementary Methods – Synthesis of 2–4.

General Methods. All reagents were purchased from commercial sources and used without further purification unless otherwise noted. Solvents used in reactions were purified by passage through alumina and copper columns under argon. Reactions were monitored by TLC on silica gel G-25 F_{254} (0.25 mm) and TLC spots were detected under UV light and charring after staining with *p*-anisaldehyde in ethanol, acetic acid, and H_2SO_4. Column chromatography was performed on silica gel 60 (40–60 μM) unless otherwise noted. ^{1}H NMR spectra were recorded at 500 MHz, 600 MHz or 700 MHz and were referenced to the solvent residual proton signal of $CDCl_3$ (7.26 ppm), D_2O (4.79 ppm) or CD_3OD (3.30 ppm). $^{13}C\{^1H\}$ NMR spectra were recorded at 126 MHz, 151 MHz or 176 MHz and were referenced to $CDCl_3$ (77.06 ppm), external acetone (31.07 ppm, D_2O), or CD_3OD (49.00 ppm). ^{31}P NMR spectra were ^{1}H decoupled and were recorded at 202 MHz. Protons and carbons on oligosaccharides were labelled starting from the reducing end with non-primed atoms and proceeding towards the non-reducing end with a prime (‘) added for each successive carbohydrate residue. Peak assignments were made based on 2D NMR analysis (^{1}H–^{1}H COSY, HSQC and HMBC). Optical rotations were measured at 22 ± 2 °C at the sodium D line (589 nm) in a microcell (10 cm, 1 mL) and are in units of deg·mL(dm·g)$^{-1}$. Electrospray ionization (ESI) mass spectra were recorded using samples in mixtures of THF with CH_3OH and added NaCl. For high resolution mass determination, spectra were obtained by voltage scan over a narrow range at a resolution of approximately 10,000.

Figure S13. Synthesis of **2**.

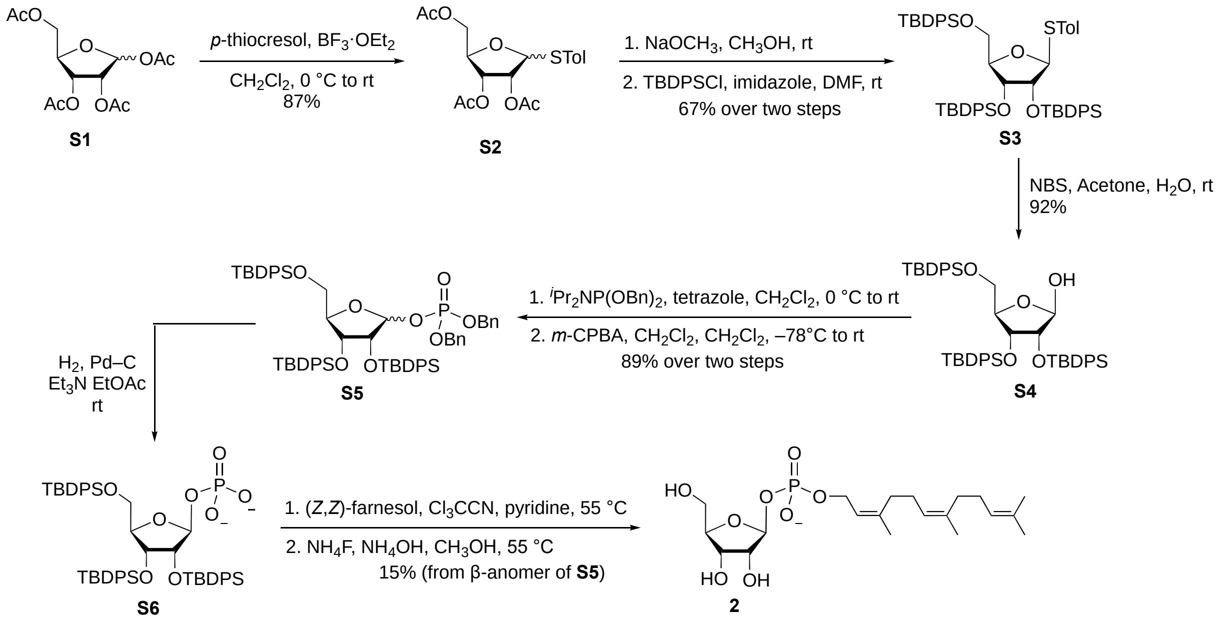

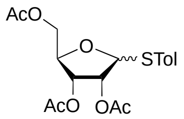

***p*-Tolyl 2,3,5-*O*-acetyl-1-thio-α/β-D-ribofuranoside (S2).** A solution of **S1** (1 g, 3.14 mmol, 1.0 equiv) and *p*-thiocresol (585.0 mg, 4.71 mmol, 1.5 equiv) in dry CH_2Cl_2 (30 mL) was cooled to 0 ºC and then $BF_3·OEt_2$ (0.5 mL, 4.71 mmol) was added dropwise. After stirring for 1 h at rt, the reaction was cooled to 0 ºC before satd aq sodium bicarbonate was added to quench the excess $BF_3·OEt_2$. The mixture was concentrated and dissolved again in EtOAc then was washed with satd aq sodium bicarbonate twice and then brine. The organic layer was dried with $MgSO_4$, filtered, and concentrated. The resulting crude residue was purified by column chromatography (hexane–EtOAc, 7:1 to 3:1) to yield **S2** (1.04 g, 87%) as white foam in a 1:3 α:β ratio: R_f 0.18 (hexane–EtOAc, 3:1); ^{1}H NMR (600 MHz, $CDCl_3$) δ 7.39–7.36 (d, *J* = 8.0 Hz, 1.5 H, Ar), 7.36–7.33 (d, *J* = 8.0 Hz, 0.5 H, Ar), 7.10 (d, *J* = 8.0 Hz, 1.5 H, Ar), 7.07 (d, *J* = 8.0 Hz, 0.5H), 5.72 (d, *J* = 5.6 Hz, 0.25H, H-1*α*), 5.41 (t, *J* = 5.9 Hz, 0.25 H, H-2*α*), 5.21 (d, *J* = 4.9 Hz, 0.75H, H-1*β*), 5.19–5.14 (m, 1.75 H, H-2*β*, H-3*β*, H-3*α*), 4.45–4.42 (m, 0.25 H, H-4*α*), 4.31 (dd, *J* = 12.2, 3.2 Hz, 0.25 H, H-5a*α*), 4.22–4.17 (m, 1.75 H, H-5b*α*, H-4*β*, H-5a*β*), 4.05–4.02 (m, 0.75 H, H-5b*β*), 2.30 (s, 6.75 H, $PhCH_3$*β*), 2.28 (s, 2.25 H, $PhCH_3$*α*), 2.13 (s, 2.25 H, (C=O)CH_3*α*), 2.09 (s, 2.25 H, (C=O)CH_3*α*), 2.06 (s, 6.75 H, (C=O)CH_3*β*), 2.03 (s, 2.25 H, (C=O)CH_3*α*), 2.03 (s, 6.75 H, (C=O)CH_3*β*), 1.99 (s, 6.75 H, (C=O)CH_3*β*); ^{13}C NMR (151 MHz, $CDCl_3$) δ 170.4 (C=O*β)*, 170.4 (C=O*α)*, 169.8 (C=O*β)*, 169.6 (C=O*α)*, 169.4 (C=O*β)*, 169.3 (C=O*α)*, 138.7 (Ar), 137.7 (Ar), 134.2 (Ar), 132.3 (Ar), 130.5 (Ar), 129.8 (Ar), 127.5 (Ar), 90.4 (C-1*β*), 87.9 (C-1*α*), 80.0 (C-4α), 77.9 (C-4*β*), 73.6 (C-2*α*), 71.4 (C-3*α*), 71.4 (C-2*β*), 70.6 (C-3*β*), 63.4 (C-5*α*), 62.9 (C-5*β*), 21.1 (Ar*C*H_3*α*), 21.0 (Ar*C*H_3*β*), 20.7 ((C=O)*C*H_3*α,* (C=O)*C*H_3*β*), 20.54 ((C=O)*C*H_3*β*), 20.5 (2 ×(C=O)*C*H_3*α*, (2 × (C=O)*C*H_3*β*); HRMS (ESI): *m/z* calcd for $C_{18}H_{22}O_7S$ $[M+Na]^+$ 405.0980; found 405.0978.

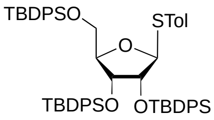

***p*-Tolyl 2,3,5-tri-*O*-*t*-butyldiphenylsilyl-1-thio-β-D-ribofuranoside (S3).** A solution of **S2** (688.0 mg, 1.80 mmol, 1.0 equiv) in CH_3OH (9 mL) was treated with sodium methoxide (29.0 mg, 0.54 mmol). After stirring at rt for 30 min, the reaction mixture was neutralized by the addition of Amberlite IR-120 H^+ resin, filtered, and concentrated. The residue was co-evaporated twice with CH_2Cl_2 and the resulting oil was then dissolved in anhydrous DMF, imidazole (1.1 g, 16.2 mmol, 9.0 equiv) and TBDPSCl (2.8 mL, 10.8 mmol, 6.0 equiv) were subsequently added. After stirring at rt for 2 days, satd aq NH_4Cl was added and the mixture was diluted in EtOAc and extracted with H_2O twice, satd aq NH_4Cl and

brine. The organic layer was dried with $MgSO_4$, filtered, and concentrated. The resulting crude product was purified by column chromatography (hexane–EtOAc, 70:1) to yield **S3** (1.17 g, 67%) as white foam: R_f 0.55 (hexane–EtOAc, 30:1); ^{1}H NMR (500 MHz, $CDCl_3$) δ 7.84–7.76 (m, 2 H, Ar), 7.79–7.72 (m, 2 H, Ar), 7.65 (m, 4 H, Ar), 7.62–7.59 (m, 2 H, Ar), 7.59–7.56 (m, 2 H, Ar), 7.52–7.33 (m, 15 H, Ar), 7.32–7.24 (m, 4 H, Ar), 7.14 (d, *J* = 8.1 Hz, 2 H, Ar), 7.01 (d, *J* = 8.0 Hz, 2 H, Ar), 5.44 (d, *J* = 4.0 Hz, 1 H, H-1), 4.41 (app t, *J* = 4.0 Hz, 1 H, H-2), 4.29 (td, *J* = 5.4, 2.9 Hz, 1 H, H-4), 4.22 (dd, *J* = 5.2, 4.1 Hz, 1 H, H-3), 3.39 (dd, *J* = 11.3, 3.0 Hz, 1 H, H-5a), 3.07 (dd, *J* = 11.3, 5.6 Hz, 1 H, H-5b), 2.35 (s, 3 H, $ArCH_3$), 1.21 (s, 9 H, $SiC(CH_3)$), 1.08 (s, 9 H, $SiC(CH_3)$), 1.00 (s, 9 H, $SiC(CH_3)$**;** ^{13}C NMR (126 MHz, $CDCl_3$) δ 136.8 (Ar), 136.4 (Ar), 136.2 (Ar), 136.0 (Ar), 135.8 (Ar), 133.8 (Ar), 133.5 (Ar), 133.4 (Ar), 133.3 (Ar), 132.1 (Ar), 131.3 (Ar), 129.9 (Ar), 129.8 (Ar), 129.7 (Ar), 129.5 (Ar), 127.7 (Ar), 127.7 (Ar), 90.9 (C-1), 84.4 (C-4), 78.1 (C-2), 74.3 (C-3), 64.8 (C-5), 27.2 ($SiC(CH_3)_3$), 27.1 ($SiC(CH_3)_3$), 26.9 ($SiC(CH_3)_3$), 21.2 ($ArCH_3$), 19.6 ($SiC(CH_3)_3$), 19.4 ($SiC(CH_3)_3$), 19.2 ($SiC(CH_3)_3$). HRMS (ESI): *m/z* calcd for $C_{60}H_{70}NaO_4SSi_3$ $[M+Na]^+$ 993.4195; found 993.4203.

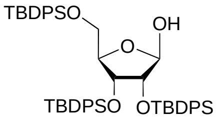

2,3,5-Tri-*O*-*t*-butyldiphenylsilyl-β-D-ribofuranose (S4). A solution of **S3** (0.29 g, 0.21 mmol, 1.0 equiv) in acetone–H_2O 9:1 (v/v, 5 mL) was treated with NBS (0.10 g, 0.58 mmol, 2,8 eqiuv). After stirring for 30 min, solid sodium bicarbonate (100 mg) was added and the reaction mixture was concentrated. The crude residue was dissolved in EtOAc and washed with H_2O until the pH of aqueous layer was neutral and then washed with brine. The organic layer was dried with $MgSO_4$, filtered and concentrated. The resulting crude product was purified by column chromatography (hexane–EtOAc, 40:1 to 30:1) to yield **S4** (231.0 mg, 92%) as white foam: R_f 0.18 (hexane–EtOAc, 30:1); ^{1}H NMR (500 MHz, $CDCl_3$) δ 7.96–7.89 (m, 2 H, Ar), 7.81 (m, 4 H, Ar), 7.73–7.68 (m, 2H, Ar), 7.56–7.33 (m, 16H, Ar), 7.33–7.25 (m, 4H, Ar), 7.22 (t, *J* = 7.6 Hz, 2H, Ar), 4.56 (d, *J* = 4.8 Hz, 1 H, H-3), 4.50 (d, *J* = 1.8 Hz, 2 H, H-1), 4.39 (app dt, *J* = 5.0, 1.9 Hz, 1 H, H-2), 3.87 (app t, *J* = 2.5 Hz, 1 H, H-4), 3.19 (dd, *J* = 11.4, 2.8 Hz, 1 H, H-5a), 2.66 (dd, *J* = 11.4, 2.3 Hz, 1 H, H-5b), 1.28 (s, 9 H, $SiC(CH_3)_3$), 1.23 (s, 9 H, $SiC(CH_3)_3$), 0.77 (s, 9 H, $SiC(CH_3)_3$); ^{13}C NMR (126 MHz, $CDCl_3$) δ 136.4 (Ar), 136.3 (Ar), 136.2 (Ar), 135.6 (Ar), 135.5 (Ar), 133.6 (Ar), 133.3 (Ar), 133.2 (Ar), 132.7 (Ar), 132.4 (Ar), 130.3 (Ar), 130.1 (Ar), 129.9 (Ar), 129.7 (Ar), 129.6 (Ar), 128.0 (Ar), 127.9 (Ar), 127.6 (Ar), 97.6 (C-1), 85.4 (C-4), 75.0 (C-3), 73.7 (C-2), 63.5 (C-5), 27.3 ($SiC(CH_3)_3$), 27.2 ($SiC(CH_3)_3$), 26.7 ($SiC(CH_3)_3$), 19.3 ($SiC(CH_3)_3$), 19.0 ($SiC(CH_3)_3$), 18.9 ($SiC(CH_3)_3$). HRMS (ESI): *m/z* calcd for $C_{53}H_{64}NaO_5Si_3$ $[M+Na]^+$ 887.3954; found 887.3951.

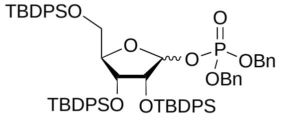

Dibenzyl (2,3,5-tri-*O*-*t*-butyldiphenyl-α/β-D-ribofuranosyl) phosphate (S5). To a solution of **S4** (231.0 mg, 0.27 mmol, 1.0 equiv) in dry CH_2Cl_2 (4 mL) was added tetrazole (4% in CH_3CN, 2.50 mL, 1.08 mmol, 4.0 equiv) at 0 ºC and then *i*-$Pr_2NP(OBn)_2$ (0.27 mL, 0.81 mmol, 1.0 equiv) was added dropwise. The reaction mixture was stirred at 0 ºC for 30 min and then was to warmed to rt. After stirring for 1.5 h, the reaction mixture was cooled to –78 ºC, *m*-CPBA (280.0 mg, 1.62 mmol) was added and then stirred for 2 h while warming to rt. The solution was diluted with CH_2Cl_2, washed with satd aq sodium bicarbonate and then brine. The organic layer was dried with Mg_2SO_4, filtered, and concentrated. The resulting crude product was purified by column chromatography (toluene–EtOAc, 45:1 to 35:1) to yield **S5** (270 mg, 89%) as a colorless oil in a 1:4 α:β ratio: Data for β-anomer; R_f 0.32 (toluene–EtOAc, 30:1); ^{1}H NMR (500 MHz, $CDCl_3$) δ 7.71 (m, 4 H, Ar), 7.57–7.48 (m, 6 H, Ar), 7.46–7.41 (m, 2 H, Ar), 7.41–7.36 (m, 4 H, Ar), 7.36–7.23 (m, 16 H, Ar), 7.18 (m, 4 H, Ar), 7.16–7.11 (m, 4 H, Ar), 5.73 (dd, *J* = 4.6, 1.6 Hz, 1 H, H-1), 4.85–4.70 (m, 4 H, 2 × ArC*H*$_2$), 4.38 (app td, *J* = 7.1, 2.5 Hz, 1 H, H-4), 4.25 (dd, *J* = 3.9, 1.7 Hz, 1 H, H-2), 4.10 (dd, *J* = 6.5, 3.9 Hz, 1 H, H-3), 3.41 (dd, *J* = 11.5, 2.5 Hz, 1 H, H-5b), 3.07 (dd, *J* = 11.5, 7.4 Hz, 1 H, H-5b), 1.14 (s, 9 H, $SiC(CH_3)_3$), 0.97 (s, 9 H, $SiC(CH_3)_3$), 0.92 (s, 9 H, $SiC(CH_3)_3$); ^{13}C NMR (126 MHz, $CDCl_3$) δ 136.2 (Ar), 136.1 (Ar), 136.0 (Ar), 135.9 (Ar), 135.7 (Ar), 135.6 (Ar), 133.5 (Ar), 133.4 (Ar), 133.2 (Ar), 132.9 (Ar), 130.0 (Ar), 129.9 (Ar), 129.7 (Ar), 129.6 (Ar), 128.5 (Ar), 128.4 (Ar), 128.3 (Ar), 128.0 (Ar), 127.9 (2 Ar), 127.8 (Ar), 127.7 (Ar), 104.0 (d, *J* = 6.4 Hz, C-1), 84.9 (C-4), 77.7 (d, *J* = 9.1 Hz, C-2), 73.2 (C-3), 69.0 (d, *J* = 5.3 Hz, Ar*C*H$_2$), 68.9 (d, *J* = 5.0 Hz, Ar*C*H$_2$), 65.5 (C-5), 27.1 (2 × SiC(*C*H$_3$)$_3$), 26.8 (SiC(*C*H$_3$)$_3$), 19.6 (Si*C*(CH$_3$)$_3$), 19.3 (Si*C*(CH$_3$)$_3$), 19.2 (Si*C*(CH$_3$)$_3$); ^{31}P NMR (202 MHz, $CDCl_3$) δ –1.93. HRMS (ESI): *m/z* calcd for $C_{67}H_{77}NaO_8PSi_3$ $[M+Na]^+$ 1147.4556; found 1147.4549.

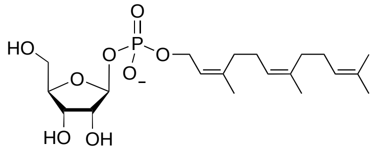

(2*Z*,6*Z*)-Farnesylphosphoryl-β-D-ribofuranose (2). A solution of **S5** (74.0 mg, 0.066 mmol, 1.0 equiv) in 10% EtOH–EtOAc (3 mL) were added Et_3N (230 µL, 1.65 mmol) and 5% palladium on carbon (wetted with water) (312.0 mg, 0.066 mmol). The reaction vessel was purged with argon and then equipped with a hydrogen-filled balloon. The reaction mixture was stirred at rt for 16 h before being filtered through a pad of Celite®, which was washed with 10% EtOH–EtOAc. The filtrate was concentrated to give a quantitative yield

of **S6**, which was directly used for the next step without purification or characterization. Thus, all of compound **S6** and (2*Z*,6*Z*)-farnesol (23) (44.0 mg, 0.198 mmol, 3.0 equiv) were azeotropically dried with toluene three times. The mixture was dissolved in pyridine (1.5 mL) and Cl_3CCN (67.0 μL, 0.66 mmol, 10.0 equiv) was added. The reaction mixture was stirred at 55 ºC for 18 h before being cooled to rt and concentrated. The crude phosphodiester was dissolved in a solution of 15% NH_4OH in CH_3OH (1.5 mL), then NH_4F (73.0 mg, 1.97 mmol, 30.0 equiv) was added. After stirring overnight at 55 ºC, the solution was filtered through a pad of Celite® using 15% NH_4OH in CH_3OH as the eluant and the filtrate was concentrated. The crude product then was purified by column chromatography (CH_2Cl_2–CH_3OH–H_2O, 9:2:0.1) to yield **2** (4.3 mg, 15% from **S5**) as a colorless oil: ^{1}H NMR (500 MHz, CD_3OD) δ 5.47 (d, *J* = 4.9 Hz, 1 H, H-1), 5.41 (app t, *J* = 7.1 Hz, 1 H, OCH_2CH=C), 5.15–5.11 (m, 1 H, CH_2CH=C), 4.40 (app t, *J* = 7.0 Hz, 1 H, OCH_2CH=C), 4.28 (dd, *J* = 7.6, 4.5 Hz, 1 H, H-3), 3.98 (d, *J* = 4.5 Hz, 1 H, H-2), 3.95 (ddd, *J* = 7.8, 5.1, 2.9 Hz, 1 H, H-4), 3.80 (dd, *J* = 12.2, 2.8 Hz, 1 H, H-5a), 3.61 (dd, *J* = 12.2, 5.2 Hz, 1 H, H-5b), 2.15–2.00 (m, 6 H, allylic CH_2), 1.74 (s, 3 H, CH_3), 1.68 (s, 4H), 1.61 (s, 3 H, CH_3). ^{31}P NMR (202 MHz, CD_3OD) δ -0.10; ^{13}C NMR (126 MHz, CD_3OD) δ 139.3 (OCH_2CH=*C*), 135.2 (CH_2CH=*C*), 131.0 (CH_2CH=*C*), 124.4 (CH_2*C*H=C), 124.0 (CH_2*C*H=C), 122.0 (d, *J* = 7.9 Hz, OCH_2*C*H=C), 103.0 (d, *J* = 5.7 Hz, C-1), 83.9 (C-4), 75.8 (d, *J* = 9.2 Hz, C-2), 69.9 (C-3), 62.3 (C-5), 61.9 (d, *J* = 5.5 Hz, O*C*H_2CH=C), 31.9 (allylic CH_2), 31.5 (allylic CH_2), 26.3 (allylic CH_2), 26.2 (allylic CH_2), 24.5 (CH_3), 22.3 (CH_3), 16.3 (CH_3). HRMS (ESI): *m/z* calcd for $C_{20}H_{34}O_8P$ $[M–H]^-$ 433.1997; found 433.1992.

Figure S14. Synthesis of **3**.

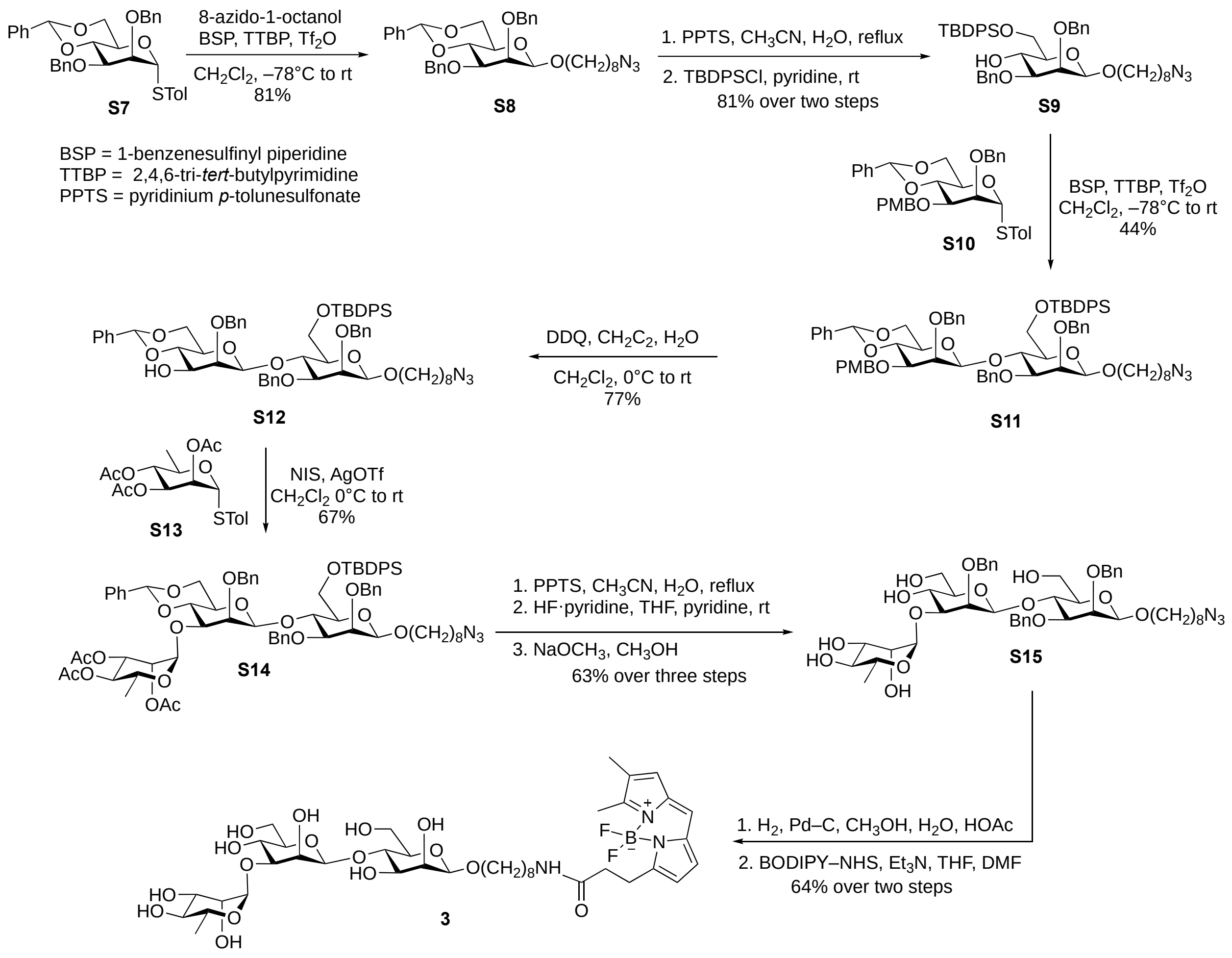

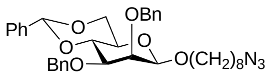

8-Azidooctyl 2,3-di-*O*-benzyl-4,6-*O*-benzylidene-β-D-mannopyranoside (S8). Thioglycoside donor **S7** (24, 25)(1.32 g, 2.42 mmol, 1.0 equiv), 1-benzenesulfinyl pyrrolidine (520 mg, 3.62 mmol, 1.1 equiv), and 2,4,6-tri-*t*-butyl-pyrimidine (1.20 g, 4.84 mmol, 2.0 equiv) were dissolved in dry CH_2Cl_2 (60 mL). Powdered 4 Å M.S. were added and the solution was stirred under argon for 20 min at rt before being cooled to –78 ℃. Trifluoromethanesulfonic anhydride (487 μL, 2.90 mmol, 1.2 equiv) was added and the reaction mixture stirred for 10 min at –78 °C. A solution of 8-azidooctanol (621 mg, 3.62 mmol, 1.5 equiv) in dry CH_2Cl_2 (5 mL) was then added dropwise over 5 min and the reaction mixture was warmed to rt over 1 h and then stirred at rt for 1 h. The excess acid was quenched by the addition of triethylamine (1 mL) and the reaction mixture was then filtered into a separatory funnel and washed with satd aq sodium bicarbonate solution. The organic layer was dried with sodium sulfate, filtered, and concentrated. The resulting

residue was purified by flash silica chromatography (hexanes–EtOAc, 9:1) to afford **S8** (1.18 g, 81%) as a clear, colourless oil: R_f = 0.25 (hexanes–EtOAc, 9:1); $[\alpha]^{21}_D$ –51.0 (*c* 0.85, $CHCl_3$); ^{1}H NMR (500 MHz, $CDCl_3$) δ 7.53–7.44 (m, 4 H, Ar), 7.40–7.24 (m, 11 H, Ar), 5.62 (s, 1 H, C*H*Ar), 4.99 (d, *J* = 12.4 Hz, 1 H, C*H*$_2$Ar), 4.88 (d, *J* = 12.4 Hz, 1 H, C*H*$_2$Ar), 4.69 (d, *J* = 12.6 Hz, 1 H, C*H*$_2$Ar), 4.59 (d, *J* = 12.6 Hz, 1 H, C*H*$_2$Ar), 4.45 (d, *J* = 0.7 Hz, 1 H, H-1), 4.31 (dd, *J* = 10.4, 4.9 Hz, 1 H, H-6a), 4.22 (app t, *J* = 9.6 Hz, 1 H, H-4), 4.00–3.89 (m, 3 H, H-6b, octyl OCH_2, H-2), 3.59 (dd, *J* = 9.9, 3.1 Hz, 1 H, H-3), 3.43 (dt, *J* = 9.3, 6.7 Hz, 1 H, octyl OCH_2), 3.33 (app td, *J* = 10.0, 4.9 Hz, 1 H, H-5), 3.26 (t, *J* = 6.9 Hz, 2 H, CH_2N_3), 1.69–1.56 (m, 4 H, octyl CH_2), 1.44–1.29 (m, 8 H, octyl CH_2); ^{13}C NMR (126 MHz, $CDCl_3$) δ 138.5, 138.4, 137.7, 128.9, 128.7, 128.3, 128.2, 128.1, 127.59, 127.56, 126.1, 102.4 (*C*HAr), 101.5 (C-1, $^1J_{C-1,-1}$ = 155.3 Hz), 78.7 (C-4), 78.0 (C-3), 75.8 (C-6), 74.7 (*C*H$_2$Ar), 72.4 (*C*H$_2$Ar), 70.3 (octyl OCH_2), 68.7 (C-6), 67.7 (C-5), 51.5 (CH_2N_3), 29.7 (octyl CH_2), 29.3 (octyl CH_2), 29.1 (octyl CH_2), 28.9 (octyl CH_2), 26.7 (octyl CH_2), 26.1 (octyl CH_2); HRMS (ESI): *m/z* calcd for $C_{35}H_{43}NaN_3O_6$ [M + Na]$^+$ 624.3044; found 624.3032.

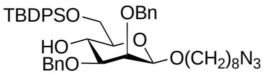

8-Azidooctyl 2,3-di-*O*-benzyl-6-*O*-*t*-butyldiphenylsilyl-β-D-mannopyranoside (S9). Glycoside **S8** (1.55 g, 2.58 mmol, 1.0 equiv) and pyridinium *p*-toluenesulfonate (1.30 g, 5.16 mmol, 2.0 equiv) were dissolved in CH_3CN–H_2O (9:1, 30 mL). The resulting solution was heated at reflux overnight. The reaction mixture was cooled to rt, diluted with CH_2Cl_2 (100 mL), and transferred to a separatory funnel where it was washed with satd aq sodium bicarbonate solution. The organic layer was dried with sodium sulfate, filtered, and concentrated. The resulting crude residue was dissolved in pyridine (15 mL) and to this solution triethylamine (1.07 mL, 7.74 mmol, 2.0 equiv) and *t*-butyl(chloro)diphenylsilane (1.00 mL, 3.87 mmol, 1.5 equiv) were added. The reaction mixture was stirred overnight then the excess *t*-butyl(chloro)diphenylsilane was quenched by the addition of CH_3OH (5 mL), and the solution was then diluted with EtOAc (75 mL) and transferred to a separatory funnel. The organic layer was washed with 1M aqueous HCl solution and satd aq sodium bicarbonate solution and then dried with sodium sulfate, filtered, and concentrated. The resulting residue was purified by flash silica chromatography (hexanes–EtOAc, 6:1) to afford **S9** (1.58 g, 81%) as a clear, colourless oil: R_f = 0.23 (hexanes–EtOAc, 5:1); $[\alpha]^{21}_D$ –51.7 (*c* 0.82, $CHCl_3$); ^{1}H NMR (500 MHz, $CDCl_3$) δ 7.73–7.67 (m, 4 H, Ar), 7.48–7.21 (m, 16 H, Ar), 4.97 (d, *J* = 12.4 Hz, 1 H, C*H*$_2$Ar), 4.77 (d, *J* = 12.4 Hz, 1 H C*H*$_2$Ar), 4.54 (d, *J* = 11.9 Hz, 1 H, C*H*$_2$Ar), 4.44 (d, *J* = 11.9 Hz, 1 H, C*H*$_2$Ar), 4.40 (s, 1 H, H-1), 4.05 (app td, *J* = 9.4, 1.7 Hz, 1 H, H-4), 4.00 (dd, *J* = 10.6, 5.0 Hz, 1 H, H-6a), 3.95 (dd, *J* = 10.6, 5.6 Hz, 1 H, H-6b), 3.93–3.90 (m, 1 H, octyl OCH_2), 3.88 (d, *J* = 3.1 Hz, 1 H, H-2), 3.43–3.37 (m, 1 H, octyl OCH_2), 3.38–3.34 (m, 1 H, H-5), 3.33 (dd, *J* = 9.4, 3.0 Hz, 1 H,

H-3), 3.23 (t, J = 7.0 Hz, 2 H, CH_2N_3), 2.85 (d, J = 1.7 Hz, 1 H, OH), 1.66–1.55 (m, 4 H, octyl CH_2), 1.40–1.28 (m, 8 H, octyl CH_2), 1.05 (s, 9 H, $C(CH_3)_3$); ^{13}C NMR (126 MHz, $CDCl_3$) δ 139.0, 138.q, 135.8, 135.7, 133.3, 133.2, 129.8, 129.7, 128.5, 128.3, 128.1, 127.79, 127.77, 127.7, 127.4 (Ar), 101.8 (C-1), 81.6 (C-3), 75.7 (C-5), 73.9 (CH$_2$Ar), 73.6 (C-2), 71.3 (CH$_2$Ar), 69.8 (octyl CH_2), 69.0 (C-4), 65.3 (C-6), 51.5 (CH_2N_3), 29.7 (octyl CH_2), 29.3 (octyl CH_2), 29.2 (octyl CH_2), 28.9 (octyl CH_2), 26.8 (C(CH$_3$)$_3$), 26.7 (octyl CH_2), 26.1 (octyl CH_2), 19.3 (C(CH$_3$)$_3$); HRMS (ESI): *m/z* calcd for $C_{44}H_{57}NaN_3O_6$ [M + Na]$^+$ 774.3909; found 774.3901.

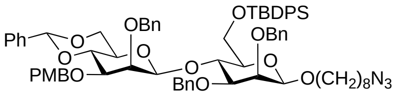

8-Azidooctyl 2-*O*-benzyl-4,6-*O*-benzylidene-3-*O*-*p*-methoxybenzyl-β-D-mannopyrannosyl-(1→4)-2,3-di-*O*-benzyl-6-*O*-*t*-butyldiphenylsilyl-β-D-mannopyranoside (S11). Thioglycoside donor **S10** (26)(0.96 g, 1.64 mmol, 1.5 equiv), 1-benzenesulfinyl pyrrolidine (351 mg, 3.62 mmol, 1.65 equiv), and 2,4,6-tri-*t*-butylpyrimidine (0.81 g, 3.23 mmol, 3.0 equiv) were dissolved in dry CH_2Cl_2 (40 mL). Powdered 4 Å M.S. were added and the solution was stirred under argon for 20 min at rt before being cooled to –78 °C. Trifluoromethanesulfonic anhydride (0.33 mL, 1.94 mmol, 1.8 equiv) was added and the reaction mixture stirred for 10 min at –78 °C. Separately, **S9** (0.81 g, 1.08 mmol, 1.0 equiv) was dissolved in dry CH_2Cl_2 (5 mL) and added to the reaction mixture dropwise over 5 min. The reaction mixture was warmed to –45 °C over 30 min and then stirred for 1 h. The excess acid was then quenched by the addition of triethylamine (1.0 mL), warmed to rt, filtered into a separatory funnel and then washed with satd aq sodium bicarbonate solution. The organic layer was dried with sodium sulfate, filtered, and concentrated. The resulting residue was purified by flash silica chromatography (hexanes–EtOAc, 9:1) to afford **S11** (0.58 g, 44%) as a clear, colourless oil: R_f = 0.32 (hexanes–EtOAc, 4:1); $[\alpha]^{21}_D$ –39.6 (*c* 1.00, $CHCl_3$); ^{1}H NMR (500 MHz, $CDCl_3$) δ 7.77–7.71 (m, 2 H, Ar), 7.71–7.64 (m, 2 H, Ar), 7.49–7.40 (m, 4 H, Ar), 7.40–7.17 (m, 24 H, Ar), 6.86–6.80 (m, 2 H, Ar), 5.52 (s, 1 H, C*H*Ar), 4.91 (d, J = 12.2 Hz, 1 H, C*H*$_2$Ar), 4.82 (d, J = 12.2 Hz, 1 H, C*H*$_2$Ar), 4.78–4.71 (m, 2 H, C*H*$_2$Ar), 4.68 (d, J = 11.9 Hz, 1 H, C*H*$_2$Ar), 4.66 (d, J = 1.0 Hz, 1 H, H-1'), 4.62 (d, J = 11.8 Hz, 1 H, C*H*$_2$Ar), 4.58 (d, J = 12.0 Hz, 1 H, C*H*$_2$Ar), 4.51 (d, J = 11.8 Hz, 1 H, C*H*$_2$Ar), 4.41 (d, J = 0.8 Hz, 1 H, H-1), 4.31 (app t, J = 9.0 Hz, 1 H, H-4), 4.08 (app t, J = 9.5 Hz, 1 H, H-4'), 4.05 (dd, J = 10.4, 4.9 Hz, 1 H, H-6a'), 3.93 (dd, J = 11.0, 2.3 Hz, 1 H, H-6a), 3.90 (dt, J = 9.3, 6.6 Hz, 1 H, octyl OCH_2), 3.86 (d, J = 3.2 Hz, 1 H, H-2), 3.83 (dd, J = 11.0, 4.5 Hz, 1 H, H-6b), 3.79 (s, 3 H, OCH_3), 3.77 (d, J = 3.0 Hz, 1 H, H-2'), 3.65 (app t, J = 10.3 Hz, 1 H, H-6b'), 3.53 (dd, J = 9.0, 3.2 Hz, 1 H, H-3), 3.46 (dd, J = 9.8, 3.0 Hz, 1 H, H-3'), 3.39 (app dt, J = 9.3, 6.6 Hz, 1 H, octyl OCH_2), 3.31 (ddd, J = 9.0, 4.5, 2.2 Hz, 1 H, H-5), 3.23 (t, J = 7.0

Hz, 2 H, CH_2N_3), 3.13 (app td, J = 9.9, 4.9 Hz, 1 H, H-5'), 1.66–1.55 (m, 4 H, octyl CH_2), 1.40–1.24 (m, 8 H, octyl CH_2), 1.02 (s, 9 H, $C(CH_3)_3$); ^{13}C NMR (126 MHz, $CDCl_3$) δ 159.2, 139.3, 139.1, 138.8, 137.76, 136.0, 135.6, 134.0, 133.3, 130.6, 129.7, 129.6, 129.1, 128.8, 128.22, 128.18, 128.09, 128.05, 127.0, 127.9, 127.8, 127.6, 127.4, 127.33, 127.31, 127.1, 126.2, 113.8 (Ar), 102.3 (C-1', $^1J_{C\text{-}1,\,H\text{-}1}$ = 153.4 Hz), 101.39 (C-1), 101.37 (*C*HAr), 80.0 (C-3), 78.8 (C-4'), 78.3 (C-3'), 77.7 (C-2'), 76.2 (C-5), 75.9 (C-4), 75.3 (C-2), 75.1 (*C*H_2Ar), 73.8 (*C*H_2Ar), 72.22 (*C*H_2Ar), 72.17 (*C*H_2Ar), 69.4 (octyl OCH_2), 68.7 (C-6'), 67.4 (C-5'), 63.3 (C-6), 55.3 (OCH_3), 51.5 (CH_2N_3), 29.8 (octyl CH_2), 29.4 (octyl CH_2), 29.2 (octyl CH_2), 28.9 (octyl CH_2), 26.8 ($C(CH_3)_3$), 26.7 (octyl CH_2), 26.2 (octyl CH_2), 19.4 (*C*$(CH_3)_3$); HRMS (ESI): *m/z* calcd for $C_{72}H_{85}NaN_3O_{12}$ [M + Na]$^+$ 1234.5795; found 1234.5795.

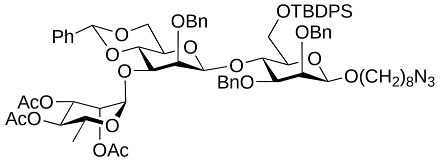

8-Azidooctyl 2,3,4-tri-*O*-acetyl-6-deoxy-α-D-mannopyranosyl-(1→3)-2-*O*-benzyl-4,6-*O*-benzylidene-β-D-mannopyrannosyl-(1→4)-2,3-di-*O*-benzyl-6-*O*-*t*-butyl-diphenylsilyl-β-D-mannopyranoside (S14). Disaccharide **S11** (723 mg, 0.596 mmol, 1.0 equiv) was dissolved in CH_2Cl_2–H_2O (19:1, 10 mL) and cooled to 0 ℃ before 2,3-dichloro-5,6-dicyano-1,4-benzoquinone (338 mg, 1.49 mmol, 2.5 equiv) was added in a single portion. The reaction mixture was subsequently warmed to rt and stirred for 80 min before the addition of satd aq sodium bicarbonate solution. The resulting biphasic solution was diluted with CH_2Cl_2 (30 mL) and transferred to a separatory funnel. The organic layer was separated, dried with sodium sulfate, filtered, and concentrated. The resulting residue was purified by flash silica chromatography (hexanes–EtOAc, 5:1) to afford **S12** (498 mg, 77%) as a clear, yellow oil: R_f = 0.21 (hexanes–EtOAc, 4:1); HRMS (ESI): *m/z* calcd for $C_{64}H_{77}N_3O_{11}Na$ [M + Na]$^+$ 1114.5220, found 1114.5204. Disaccharide alcohol **S12** (93 mg, 0.085 mmol, 1.0 equiv) and thioglycoside **S13**[4] (40 mg, 0.102 mmol, 1.2 equiv) were dissolved in dry CH_2Cl_2 (5 mL) and stirred with 4 Å M.S. under argon for 15 min at rt. The solution was then cooled to 0 ℃ and *N*-iodosuccinimide (29 mg, 0.128 mmol, 1.5 equiv) and silver trifluoromethanesulfonate (2.3 mg, 0.009 mmol, 0.1 equiv) were added. The reaction mixture was warmed to rt and stirred for 45 min before the excess acid was quenched by the addition of triethylamine (0.2 mL). The solution was diluted with EtOAc and filtered into a separatory funnel where it was washed with satd solutions of sodium thiosulfate and sodium bicarbonate. The organic layer was dried with sodium sulfate, filtered, and concentrated. The resulting residue was purified by flash silica chromatography (hexanes–EtOAc, 4:1) to afford **S14** (78 mg, 67%) as a clear, colourless oil: R_f = 0.17 (hexanes–EtOAc, 3:1); $[\alpha]^{21}_D$ –39.1 (c 0.24, $CHCl_3$); ^{1}H NMR (500

MHz, $CDCl_3$) δ 7.78–7.65 (m, 4 H, Ar), 7.47–7.41 (m, 2 H, Ar), 7.41–7.17 (m, 24 H, Ar), 5.47 (s, 1 H, C*H*Ar), 5.42 (dd, *J* = 3.5, 1.9 Hz, 1 H, H-2''), 5.35 (dd, *J* = 10.1, 3.5 Hz, 1 H, H-3''), 5.17 (d, *J* = 1.9 Hz, 1 H, H-1''), 5.04 (app t, *J* = 9.9 Hz, 1 H, H-4''), 4.93 (d, *J* = 12.3 Hz, 1 H, CH_2Ar), 4.81 (d, *J* = 12.2 Hz, 1 H, CH_2Ar), 4.76 (s, 1 H, H-1'), 4.75 (d, *J* = 11.6 Hz, 1 H, CH_2Ar), 4.70 (d, *J* = 12.0 Hz, 1 H, CH_2Ar), 4.64 (d, *J* = 11.5 Hz, 1 H, CH_2Ar), 4.59 (d, *J* = 12.0 Hz, 1 H, CH_2Ar), 4.44 (s, 1 H, H-1), 4.35 (app t, *J* = 9.0 Hz, 1 H, H-4), 4.08 (app t, *J* = 9.6 Hz, 1 H, H-4'), 4.00 (dd, *J* = 10.2, 4.7 Hz, 1 H, H-6a'), 3.98 (dd, *J* = 11.1, 2.3 Hz, 1 H, H-6a), 3.95–3.87 (m, 3 H, H-6b, octyl OCH_2, H-2), 3.81–3.73 (m, 3 H, H-3', H-5'', H-2'), 3.65–3.55 (m, 2 H, H-6b', H-3), 3.44–3.35 (m, 2 H, octyl OCH_2, H-5), 3.23 (t, *J* = 7.0 Hz, 2 H, CH_2N_3), 3.07 (app dt, *J* = 9.9, 5.0 Hz, 1 H, H-5'), 2.07 (s, 3 H, CH_3CO), 2.05 (s, 3 H, CH_3CO), 2.00 (s, 3 H, CH_3CO), 1.67–1.55 (m, 4 H, octyl CH_2), 1.39–1.29 (m, 8 H, octyl CH_2), 1.12 (d, *J* = 6.2 Hz, 3 H, H-6'''), 1.04 (d, *J* = 2.0 Hz, 9 H, $C(CH_3)_3$); ^{13}C NMR (126 MHz, $CDCl_3$) δ 169.9 (C=O), 169.8 (C=O), 169.7 (C=O), 139.2, 138.9, 138.4, 137.3, 136.0, 135.5, 133.9, 133.3, 129.8, 129.7, 128.8, 128.32, 128.28, 128.2, 128.1, 128.0, 127.9, 127.84, 127.79, 127.6, 127.53, 127.47, 127.4, 127.3, 127.2, 126.0 (Ar), 101.7 (C-1'), 101.4 (C-1), 101.1 (*C*HAr), 98.3 (C-1'', $^1J_{C-1, H-1}$ = 176.6 Hz), 80.1 (C-3), 79.0 (C-2'), 78.8 (C-4'), 76.1 (C-5), 76.0 (C-3'), 75.6 (CH_2Ar), 75.3 (C-4), 75.1 (C-2), 73.9 (CH_2Ar), 71.9 (CH_2Ar), 71.2 (C-4''), 69.6 (C-2''), 69.5 (octyl OCH_2), 68.9 (C-3''), 68.4 (C-6'), 67.0 (C-5'), 66.9 (C-5''), 63.3 (C-6), 51.5 (CH_2N_3), 29.8 (octyl CH_2), 29.4 (octyl CH_2), 29.2 (octyl CH_2), 28.9 (octyl CH_2), 26.8 ($C(CH_3)_3$), 26.7 (octyl CH_2), 26.2 (octyl CH_2), 20.9 (CH_3CO), 20.79 (CH_3CO), 20.75 (CH_3CO), 19.4 ($C(CH_3)_3$), 17.6 (C-6''); HRMS (ESI): *m/z* calcd for $C_{76}H_{93}NaN_3O_{18}Si$ $[M + Na]^+$ 1386.6116; found 1386.6125.

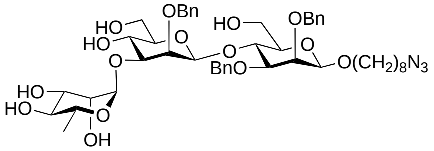

8-Azidooctyl 6-deoxy-α-D-mannopyranosyl-(1→3)-2-*O*-benzyl-β-D-mannopyrannosyl-(1→4)-2,3-di-*O*-benzyl-β-D-mannopyranoside (S15). Trisaccharide **S14** (137 mg, 0.104 mmol, 1.0 equiv) and pyridinium *p*-toluenesulfonate (63 mg, 0.251 mmol, 2.5 equiv) were dissolved in CH_3CN–H_2O (9:1, 20 mL), and heated at reflux overnight. The reaction mixture was cooled to rt, diluted with CH_2Cl_2 (100 mL), and transferred to a separatory funnel where it was washed with satd aq sodium bicarbonate solution. The organic layer was dried with sodium sulfate, filtered, and concentrated. The resulting residue was dissolved in THF–pyridine (5:1, 6 mL). Hydrogen fluoride (70% in pyridine, 0.18 mL) was added, and the reaction mixture stirred for 24 h before the excess acid was quenched by the addition of satd aq sodium bicarbonate solution (100 mL). The resulting solution was diluted with CH_2Cl_2 (60 mL) and transferred to a separatory funnel where the organic layer was separated, dried with sodium sulfate,

filtered, and concentrated. The resulting crude residue was dissolved in CH_3OH (20 mL) and sodium methoxide (cat., 0.1M in CH_3OH) was added. The reaction mixture stirred for 3 h before the sodium methoxide was neutralized by the addition of prewashed Amberlite® IR 120 acidic resin. The solution was filtered, concentrated and the resulting residue was purified by flash silica chromatography (CH_2Cl_2–CH_3OH, 9:1) to afford **S15** (56 mg, 63% over three steps) as a clear, colourless oil: R_f = 0.10 (CH_2Cl_2–CH_3OH, 9:1); ^{1}H NMR (500 MHz, CD_3OD) δ 7.43–7.37 (m, 4 H, Ar), 7.33–7.16 (m, 11 H, Ar), 5.09 (d, *J* = 1.8 Hz, 1 H, H-1''), 4.88 (d, *J* = 11.4 Hz, 1 H, CH_2Ar), 4.84–4.76 (m, 4 H, CH_2Ar), 4.74 (s, 1 H, H-1'), 4.54 (d, *J* = 11.8 Hz, 1 H, CH_2Ar), 4.54 (s, 1 H, H-1), 4.10 (app t, *J* = 9.6 Hz, 1 H, H-4), 4.02–3.97 (m, 2 H, H-2'', H-2), 3.93 (dt, *J* = 9.9, 6.7 Hz, 1 H, octyl OCH_2), 3.91–3.87 (m, 2 H, H-6a, H-2'), 3.80–3.73 (m, 1 H, H-5''), 3.74–3.67 (m, 4 H, H-6a', H-3'', H-4', H-6b), 3.64 (dd, *J* = 9.6, 3.3 Hz, 1 H, H-3), 3.61 (dd, *J* = 9.6, 3.0 Hz, 1 H, H-3'), 3.56 (dd, *J* = 11.9, 6.6 Hz, 1 H, H-6b'), 3.49 (dt, *J* = 9.4, 6.4 Hz, 1 H, octyl OCH_2), 3.41 (app t, *J* = 9.5 Hz, 1 H, H-4''), 3.36 (ddd, *J* = 9.6, 4.5, 2.2 Hz, 1 H, H-5), 3.25 (t, *J* = 6.9 Hz, 2 H, CH_2N_3), 3.15 (ddd, *J* = 9.3, 6.6, 2.4 Hz, 1 H, H-5'), 1.67–1.52 (m, 4 H, octyl CH_2), 1.45–1.32 (m, 8 H, octyl CH_2), 1.26 (d, *J* = 6.2 Hz, 3 H, H-6''); ^{13}C NMR (126 MHz, CD_3OD) δ 140.3, 139.9, 129.5, 129.32, 129.26, 129.2, 129.0, 128.9, 128.6, 128.5 (Ar), 103.8 (C-1''), 102.8 (C-1), 101.5 (C-1'), 81.5 (C-3'), 80.7 (C-3), 80.5 (C-2'), 78.6 (C-5'), 77.6 (C-5), 77.3 (C-2), 76.2 (*C*H_2Ar), 75.7 (*C*H_2Ar), 75.6 (C-4), 73.9 (C-4''), 73.7 (*C*H_2Ar), 72.4 (C-3''), 72.1 (C-2''), 70.8 (octyl OCH_2), 70.5 (C-5''), 68.9 (C-4'), 63.2 (C-6'), 62.5 (C-6), 52.5 (CH_2N_3), 30.8 (octyl CH_2), 30.4 (octyl CH_2), 30.2 (octyl CH_2), 29.9 (octyl CH_2), 27.7 (octyl CH_2), 27.2 (octyl CH_2), 18.1 (C-6''). HRMS (ESI): *m/z* calcd for $C_{47}H_{65}NaN_3O_{15}$ $[M + Na]^+$ 934.4308; found 934.4307.

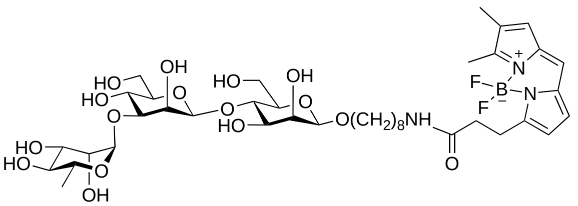

8-Aminooctyl 6-deoxy-α-D-mannopyranosyl-(1→3)-β-D-mannopyrannosyl-(1→4)-β-D-mannopyranoside BODIPY conjugate (3). Trisaccharide **S15** (26 mg, 0.029 mmol, 1.0 equiv) was dissolved in CH_3OH–H_2O (1:1, 4 mL), AcOH (3 drops) was added, and the reaction flask was purged with argon before Pd/C (26 mg) was added to the solution and the reaction flask was purged and charged with H_2 (3 x) and stirred overnight. The reaction mixture was filtered and the filtrate was concentrated. The resulting crude residue was dissolved in THF–DMF (1:1, 4 mL) and cooled to 0 ℃ before BODIPY-NHS ester (23 mg, 0.059 mmol, 2.0 equiv) and triethylamine (1 drop) were added to the solution. The reaction mixture was stirred for 1 h, and then AcOH (3 drops) was added before concentration. The crude residue was purified by C_{18} column chromatography

(Waters 5 g SepPak, 1% AcOH in H_2O to 1% AcOH in H_2O–CH_3OH, 1:1) and then size exclusion chromatography (Sephadex, LH-20, CH_3OH) to afford **3** (16 mg, 64%) as a dark red solid: R_f = 0.41 (EtOAc–CH_3OH–AcOH–H_2O, 12:3:3:2); ^1H NMR (700 MHz, CD_3OD) δ 7.42 (s, 1 H, Ar), 7.00 (d, *J* = 4.1 Hz, 1 H, Ar), 6.31 (d, *J* = 4.1 Hz, 1 H, Ar), 6.21 (s, 1 H, alkene CH), 4.99 (d, *J* = 1.8 Hz, 1 H, H-1''), 4.66 (d, *J* = 0.9 Hz, 1 H, H-1'), 4.50 (d, *J* = 1.0 Hz, 1 H, H-1), 3.99 (dd, *J* = 3.1, 1.0 Hz, 1 H, H-2'), 3.97 (dd, *J* = 3.5, 1.8 Hz, 1 H, H-2''), 3.93–3.85 (m, 4 H, H-2, H-4, H-6a, octyl CH_2), 3.85–3.78 (m, 2 H, H-6a', H-5''), 3.75 (dd, *J* = 9.5, 3.5 Hz, 1 H, H-3''), 3.70 (dd, *J* = 12.1, 4.2 Hz, 1 H, H-6b'), 3.67 (dd, *J* = 12.0, 6.5 Hz, 1 H, H-6b), 3.64 (app t, *J* = 9.7 Hz, 1 H, H-4'), 3.63 (dd, *J* = 9.4, 3.1 Hz, 1 H, H-3), 3.54–3.50 (m, 2 H, H-3', octyl OCH_2), 3.39 (app t, *J* = 9.5 Hz, 1 H, H-4''), 3.33–3.28 (m, 2 H, H-5', H-5), 3.21 (t, *J* = 7.7 Hz, 2 H, BODIPY-CH_2), 3.15 (t, *J* = 7.0 Hz, 2 H, $CH_2N(C{=}O)$), 2.59 (t, *J* = 7.6 Hz, 2 H, BODIPY-CH_2), 2.51 (s, 3 H, ArCH_3), 2.28 (s, 3 H, Ar CH_3), 1.63–1.56 (m, 2 H, octyl CH_2), 1.46 (p, *J* = 7.2 Hz, 2 H, octyl CH_2), 1.36 (td, *J* = 6.4, 2.2 Hz, 2 H, octyl CH_2), 1.33–1.27 (m, 6 H, octyl CH_2), 1.25 (d, *J* = 6.3 Hz, 3 H, H-6''); ^{13}C NMR (176 MHz, CD_3OD) δ 174.5 (C=O), 161.3, 158.6, 145.8, 136.5, 134.9, 129.6, 125.8, 121.4 (Ar), 117.7 (alkene CH), 104.3 (C-1''), 101.8 (C-1), 101.5 (C-1'), 82.6 (C-3'), 78.7 (C-5'), 77.3 (C-4), 76.8 (C-5), 74.0 (C-4''), 73.6 (C-3), 72.4 (C-2'), 72.2 (C-3''), 72.1 (C-2''), 72.0 (C-2), 70.7 (octyl OCH_2), 70.2 (C-5''), 67.5 (C-4'), 62.8 (C-6), 62.1 (C-6'), 40.4 (*C*H_2NCO), 36.1 (BODIPY-*C*H_2), 30.7 (octyl CH_2), 30.5 (octyl CH_2), 30.3 (octyl CH_2), 27.9 (octyl CH_2), 27.1 (octyl CH_2), 25.7 (octyl CH_2), 21.2 (BODIPY- *C*H_2), 18.0 (C-6''), 14.9 (CH_3), 11.2 (CH_3); HRMS (ESI): *m/z* calcd for $C_{40}H_{62}BF_2NaN_3O_{16}$ $[M + Na]^+$ 912.4083; found 912.4092.

Figure S15. Synthesis of **4**.

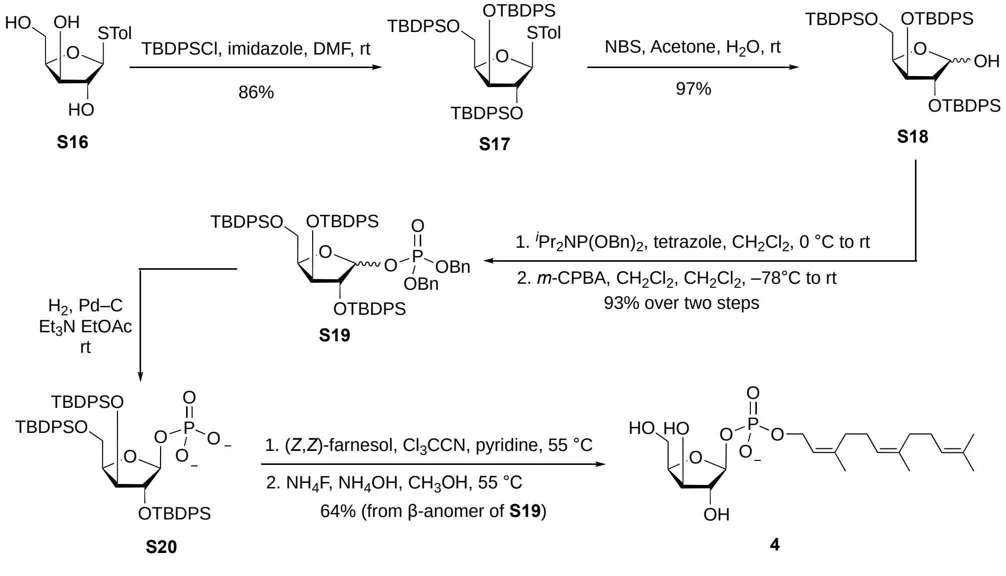

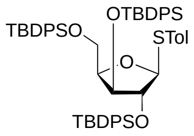

***p*-Tolyl 2,3,5-tri-*O*-*t*-butyldiphenyl-1-thio-β-D-xylofuranoside (S17).** To a solution of **S16** (27)(2.6 g, 10.0 mmol, 1.0 equiv) in dry CH_2Cl_2–DMF 10:1 (v/v, 110.0 mL) was added imidazole (6.1 g, 90.0 mmol, 9.0 equiv), followed by TBDPSCl (15.6 mL, 60.0 mmol, 6.0 equiv). After stirring overnight, excess TBDPSCl was quenched by the addition of ice-cold water and the solution was extracted with EtOAc. The organic layer was washed with brine, dried with $MgSO_4$, filtered, and concentrated. The resulting crude residue was purified by column chromatography (hexane–EtOAc, 40:1) to yield **S17** (8.4 g, 86%) as white solid: R_f 0.36 (hexane–EtOAc, 40:1); ^{1}H NMR (500 MHz, $CDCl_3$) δ 7.75–7.72 (m, 2 H, Ar), 7.59 (m, 2 H, Ar), 7.52–7.37 (m, 15 H, Ar), 7.37–7.27 (m, 5 H, Ar), 7.27–7.22 (m, 3 H, Ar), 7.17–7.13 (m, 5 H, Ar), 7.09 (dd, *J* = 7.8, 6.1 Hz, 2 H, Ar), 5.30 (s, 1 H, H-1), 4.50 (s, 1 H, H-2), 4.41–4.38 (m, 1 H, H-4), 4.13 (d, *J* = 3.1 Hz, 1 H, H-3), 4.09 (dd, *J* = 11.0, 7.8 Hz, 1 H, H-5a), 3.68 (dd, *J* = 11.1, 3.9 Hz, 1 H, H-5b), 2.36 (s, 3 H, $ArCH_3$), 1.11 (s, 9 H, $SiC(CH_3)_3$), 0.97 (s, 9 H, $SiC(CH_3)_3$), 0.91 (s, 9 H, $SiC(CH_3)_3$); ^{13}C NMR (126 MHz, $CDCl_3$) δ 136.4 (Ar) 136.1 (Ar), 136.0 (Ar), 135.8 (Ar), 135.8 (Ar), 135.7 (Ar), 134.0 (Ar), 133.6 (Ar), 133.2 (Ar), 132.9 (Ar), 132.6 (Ar), 131.3 (Ar), 130.9 (Ar), 129.9 (Ar), 129.8 (Ar), 129.6 (Ar), 129.5 (Ar), 127.8 (Ar) 127.7 (Ar) 127.6 (Ar), 95.2 (C-1), 85.1 (C-4), 83.8 (C-2), 77.7 (C-3), 64.5 (C-5), 27.1 ($SiC(\mathit{C}H_3)_3$), 27.1 ($SiC(\mathit{C}H_3)_3$), 26.9 ($SiC(\mathit{C}H_3)_3$), 21.2 ($Ar\mathit{C}H_3$), 19.4 ($Si\mathit{C}(CH_3)_3$), 19.3 ($Si\mathit{C}(CH_3)_3$), 19.2 ($Si\mathit{C}(CH_3)_3$); HRMS (ESI): *m/z* calcd for $C_{60}H_{70}NaO_4SSi_3$ $[M+Na]^+$ 993.4195; found 993.4187.

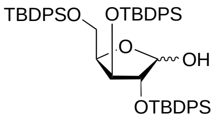

2,3,5-Tri-*O*-(*t*-butyldiphenyl)silyl-α/β-D-xylofuranose (S18). A solution of **S17** (204 mg, 0.21 mmol, 1.0 equiv) in acetone–H_2O 9:1 (v/v, 2.1 mL) was treated with NBS (75 mg, 0.42 mmol, 2.0 equiv). After stirring for 30 min, solid sodium bicarbonate (100 mg) was added and the reaction mixture was concentrated. The crude mixture was dissolved in EtOAc and washed with water until the pH of the aqueous layer was neutral, and then brine. The organic layer was dried with $MgSO_4$, filtered and concentrated. The resulting crude residue was purified by column chromatography (hexane–EtOAc, 40:1 to 30:1) to yield **S18** (176 mg, 97%) as white foam. R_f 0.05 (hexane–EtOAc, 35:1); ^{1}H NMR (500 MHz, $CDCl_3$) δ 7.74–7.67 (m, 2 H, Ar), 7.67–7.60 (m, 2 H, Ar), 7.51–7.28 (m, 20 H, Ar), 7.28–7.07 (m, 6 H, Ar), 5.41 (dd, *J* = 12.2, 3.1 Hz, 0.45 H, H-1*α*), 5.12 (d, *J* = 12.3 Hz, 0.55 H, H-1*β*), 4.33 (ddd, *J* = 7.2, 5.1, 2.5 Hz, 0.55 H, H-4*β*), 4.26–4.21 (m, 0.9 H, H-3*α*, H-4*α*), 4.13 (app s, 1.1, H-2*β*, H-3*β*), 4.05–3.98 (m, 1 H, H-2α, H-5a*β*), 3.84 (m, 1 H, H-

5b*β*, 5-aα), 3.76 (dd, *J* = 10.7, 6.0 Hz, 0.45 H, H-5b*α*), 3.72 (d, *J* = 12.2 Hz, 0.45 H, OH-1*α*), 3.34 (d, *J* = 12.3 Hz, 0.55 H, OH-1*β*), 1.11 (s, 4.95 H, $SiC(CH_3)_3$*β*), 1.09 (s, 4.05 H, $SiC(CH_3)_3$*α*), 1.01 (s, 4.05 H, $SiC(CH_3)_3$*α*), 0.97 (s, 4.55 H, $SiC(CH_3)_3$*β*), 0.91 (s, 4.05 H, $SiC(CH_3)_3$*α*), 0.89 (s, 4.55 H, $SiC(CH_3)_3$*β*). ^{13}C NMR (126 MHz, $CDCl_3$) δ 135.9 (Ar), 135.8 (Ar) 135.7 (Ar), 135.6 (Ar), 133.8 (Ar), 133.6 (Ar), 133.5 (Ar), 133.4 (Ar), 133.0 (Ar), 132.8 (Ar), 132.4 (Ar), 132.3 (Ar), 131.8 (Ar), 131.5 (Ar), 130.2 (Ar), 130.1 (Ar), 130.0 (Ar), 129.9 (Ar), 129.7 (Ar), 129.6 (Ar), 128.0 (Ar), 127.9 (Ar), 127.8 (Ar), 127.7 (Ar), 103.8 (C-1*β*), 97.8 (C-1*α*), 83.8 (C-4*β*), 80.6 (C-4*α*), 80.5 (C-2*β*), 77.3 (C-3*β*), 76.8 (C-3*α*), 76.5 (C-2*α*), 64.1 (C-5*β*), 62.5 (C-5*α*), 27.1 (2 × $SiC(\mathit{C}H_3)_3$), 27.0 ($SiC(\mathit{C}H_3)_3$), 27.0 ($SiC(\mathit{C}H_3)_3$), 27.0 ($SiC(\mathit{C}H_3)_3$), 26.9 ($SiC(\mathit{C}H_3)_3$), 19.3 ($Si\mathit{C}(CH_3)_3$), 19.3 ($Si\mathit{C}(CH_3)_3$), 19.3 ($Si\mathit{C}(CH_3)_3$), 19.3 (2 × $Si\mathit{C}(CH_3)_3$), 19.2 ($Si\mathit{C}(CH_3)_3$). HRMS (ESI): *m/z* calcd for $C_{53}H_{64}NaO_5Si_3$ $[M+Na]^+$ 887.3954; found 887.3959.

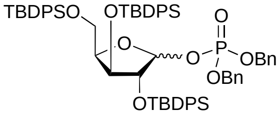

Dibenzyl (2,3,5-tri-*O*-*t*-butyldiphenylsilyl-α/β-D-xylofuranosyl) phosphate (S19). To a solution of **S18** (176 mg, 0.2 mmol, 1.0 equiv) in dry CH_2Cl_2 (4 mL) was added tetrazole (4% in CH_3CN, 2.1 mL, 0.80 mmol, 4.0 equiv) at 0 ºC then *i*-$Pr_2NP(OBn)_2$ (0.23 mL, 0.6 mmol, 3.0 equiv) was added dropwise. The reaction mixture was stirred at 0 ºC for 30 min and then warmed to rt. After stirring for 1.5 h, the reaction mixture was cooled to –78 ºC before *m*-CPBA (207.0 mg, 1.2 mmol) was added. The reaction mixture was stirred for 2 h while warming to rt, before being diluted with CH_2Cl_2, washed with satd aq sodium bicarbonate and brine. The organic layer was dried with Mg_2SO_4, filtered and concentrated. The resulting crude residue was purified by column chromatography (hexane–EtOAc, 10:1 to 5:1) to yield **S19α** (111 mg, 49%) and **S19β** (98 mg, 44%). Data for **S19β**: R_f 0.33 (hexane–EtOAc, 4:1); ^{1}H NMR (500 MHz, $CDCl_3$) δ 7.69–7.63 (m, 2 H, Ar), 7.63–7.54 (m, 2 H, Ar), 7.46–7.21 (m, 30 H, Ar), 7.18–7.13 (m, *J* = 7.8 Hz, 4 H, Ar), 7.01 (t, *J* = 7.7 Hz, 2 H, Ar), 5.72 (d, *J* = 4.5 Hz, 1 H, H-1), 5.04 (dd, *J* = 11.9, 6.6 Hz, 1 H, $O\mathit{CH_2}Ph$), 4.95 (dd, *J* = 11.9, 7.4 Hz, 1 H, $O\mathit{CH_2}Ph$), 4.84 (dd, *J* = 11.8, 6.2 Hz, 1 H, $O\mathit{CH_2}Ph$), 4.76 (dd, *J* = 11.8, 6.6 Hz, 1 H, $O\mathit{CH_2}Ph$), 4.45–4.42 (m, 1 H, H-4), 4.25 (d, *J* = 3.5 Hz, 1 H, H-3), 4.20 (s, 1 H, H-2), 4.03 (dd, *J* = 11.1, 6.6 Hz, 1 H, H-5a), 3.98 (dd, *J* = 11.1, 5.4 Hz, 1 H, H-5b), 1.06 (s, 9 H, $SiC(CH_3)_3$), 0.91 (s, 9 H, $SiC(CH_3)_3$), 0.84 (s, 9 H, $SiC(CH_3)_3$); ^{13}C NMR (126 MHz, $CDCl_3$) δ 136.2 (Ar), 136.1 (Ar), 135.9 (Ar), 135.8 (Ar), 135.7 (Ar), 133.5 (Ar), 133.2 (Ar), 132.8 (Ar), 132.4 (Ar), 130.0 (Ar), 129.9 (Ar), 129.8 (Ar), 129.7 (Ar), 128.5 (Ar), 128.3 (Ar), 128.2 (Ar), 127.9 (Ar), 127.8 (Ar), 127.8 (Ar), 127.7 (Ar), 127.6, 105 (d, *J* = 6.1 Hz, C-1), 86.5 (C-4), 81.6 (d, *J* = 10.6 Hz, C-2), 76.5 (C-3), 68.9 (d, *J* = 5.1 Hz, $Ar\mathit{C}H_2$), 68.7 (d, *J* = 5.0 Hz, $Ar\mathit{C}H_2$), 63.5 (C-5), 27.1 ($SiC(\mathit{C}H_3)_3$), 26.9

(SiC(CH_3)$_3$), 26.8 (SiC(CH_3)$_3$), 19.4 (Si*C*(CH_3)$_3$), 19.3 (Si*C*(CH_3)$_3$), 19.2 (Si*C*(CH_3)$_3$); ^{31}P NMR (202 MHz, $CDCl_3$) δ –1.49; HRMS (ESI): *m/z* calcd for $C_{67}H_{77}NaO_8PSi_3$ [M+Na]$^+$ 1147.4556; found 1147.4555.

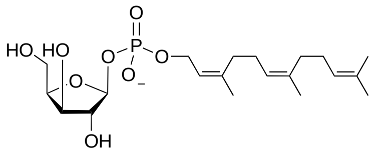

(2*Z*,6*Z*)-Farnesylphosphoryl-β-D-xylofuranose (4). To a solution of **S19β** (98.0 mg, 0.087 mmol, 1.0 equiv) in 10% EtOH in EtOAc (3 mL) were added Et_3N (0.3 mL, 2.175 mmol) and 5% palladium on carbon (wetted with 55% water) (411 mg, 0.870 mmol). The reaction vessel was purged with argon and then equipped with a hydrogen-filled balloon. The reaction mixture was stirred at rt for 16 h before being filtered through a pad of Celite®, which was washed with 10% EtOH in EtOAc. The filtrate was concentrated to give quantitative yield of **S20**, which was used directly for the next step without further purification or characterization. This all of compound **7** and (2*Z*,6*Z*)-farnesol (23) (78.0 mg, 0.348 mmol, 4.0 equiv) were azeotropically dried with toluene three times. The mixture was dissolved in pyridine (2 mL) and Cl_3CCN (87 μL, 0.87 mmol, 10.0 equiv) was added. The reaction mixture was stirred at 55 ºC for 18 h before being cooled to rt and concentrated. The crude phosphodiester was then dissolved in a solution of 15% NH_4OH in CH_3OH (3 mL), before NH_4F (97.0 mg, 2.61 mmol, 30.0 equiv) was added. After stirring overnight, the solution was filtered through a pad of Celite®, which was then washed with a solution 15% NH_4OH in CH_3OH and the filtrate was concentrated. The crude product was then was purified by column chromatography (hexane–EtOAc. 5:1 to 1:1) to yield **4** (25 mg, 64%) as a colorless oil. R_f 0.19 (9:2:0.1 CH_2Cl_2–CH_3OH–H_2O); ^{1}H NMR (500 MHz, CD_3OD) δ 5.48 (d, *J* = 5.7 Hz, 1 H, H-1), 5.42 (app t, *J* = 7.0 Hz, 1 H, OCH_2CH=C), 5.13 (m, 2 H, 2 × CH_2CH=C), 4.42 (t, *J* = 7 Hz, 2 H, OCH_2CH=C), 4.30–4.27 (m, 1 H, H-5a), 4.15 (s, 1 H, H-3), 4.12–4.09 (m, 1 H, H-4), 3.87–3.82 (m, 2 H, H-5b, H-2), 2.16–1.98 (m, 8 H, allylic CH_2), 1.74 (s, 3 H, CH_3), 1.68 (s, 6 H, 2 × CH_3), 1.61 (s, 3 H, CH_3); ^{13}C NMR (126 MHz, CD_3OD) δ 140.7 (OCH_2CH=*C*), 136.6 (CH_2CH=*C*), 132.4 (CH_2CH=C), 125.8 (CH_2*C*H=C), 125.4 (CH_2*C*H=C), 123.4 (d, *J* = 8.3 Hz, OCH_2*C*H=C), 105.3 (d, *J* = 5.6 Hz, C-1), 84.8 (C-2), 83.2 (d, C-3), 77.9 (C-4), 63.3 (d, *J* = 5.6 Hz, O*C*H_2CH=C), 62.6 (C-5), 33.3 (allylic CH_2), 32.9 (allylic CH_2), 27.7 (allylic CH_2), 27.6 (allylic CH_2), 25.9 (CH_3), 23.7 (2 × CH_3), 17.7 (CH_3); ^{31}P NMR (202 MHz, CD_3OD) δ –0.58; HRMS (ESI): *m*/z calcd for $C_{20}H_{34}O_8P$ [M – H]$^-$ 433.1997; found 433.2001.

References

1. Hitchcock, P. J., and Brown, T. M. (1983) Morphological heterogeneity among *Salmonella* lipopolysaccharide chemotypes in silver-stained polyacrylamide gels. *J. Bacteriol.* **154**, 269–77
2. Westphal, O., and Jann, K. (1965) Bacterial lipopolysaccharide-extraction with phenol water and further application of procedure. *Methods Carbohydr. Chem.* **5**, 83–91
3. Lira, L. M., Vasilev, D., Pilli, R. A., and Wessjohann, L. A. (2013) One-pot synthesis of organophosphate monoesters from alcohols. *Tetrahedron Lett*. **54**, 1690–1692
4. Ravenscroft, N., Haeuptle, M. A., Kowarik, M., Fernandez, F. S., Carranza, P., Brunner, A., Steffen, M., Wetter, M., Keller, S., Ruch, C., and Wacker, M. (2016) Purification and characterization of a *Shigella* conjugate vaccine, produced by glycoengineering *Escherichia coli*. *Glycobiol.* **26**, 51–62
5. Kelly, S. D., Williams, D. M., Nothof, J. T., Kim, T., Lowary, T. L., Kimber, M. S., and Whitfield, C. (2022) The biosynthetic origin of ribofuranose in bacterial polysaccharides. *Nat. Chem. Bio.* **18**, 530–537
6. Mann, E., Kelly, S. D., Al-Abdul-Wahid, M. S., Clarke, B. R., Ovchinnikova, O. G., Liu, B., and Whitfield, C. (2019) Substrate recognition by a carbohydrate-binding module in the prototypical ABC transporter for lipopolysaccharide O-antigen from *Escherichia coli* O9a. *J. Biol. Chem.* **294**, 14978–14990
7. Thompson, J. D., Higgins, D. G., and Gibson, T. J. (1994) CLUSTAL W: improving the sensitivity of progressive multiple sequence alignment through sequence weighting, position-specific gap penalties and weight matrix choice. *Nucleic Acids Res.* **22**, 4673–4680
8. Di Tommaso, P., Moretti, S., Xenarios, I., Orobitg, M., Montanyola, A., Chang, J.-M., Taly, J.-F., and Notredame, C. (2011) T-Coffee: a web server for the multiple sequence alignment of protein and RNA sequences using structural information and homology extension. *Nucleic Acids Res.* **39**, W13–W17
9. Robert, X., and Gouet, P. (2014) Deciphering key features in protein structures with the new ENDscript server. *Nucleic Acids Res.* **42**, W320–W324
10. Hallgren, J., Tsirigos, K. D., Damgaard Pedersen, M., Juan, J., Armenteros, A., Marcatili, P., Nielsen, H., Krogh, A., and Winther, O. (2022) DeepTMHMM predicts alpha and beta transmembrane proteins using deep neural networks. *bioRxiv*. 10.1101/2022.04.08.487609
11. Jumper, J., Evans, R., Pritzel, A., Green, T., Figurnov, M., Ronneberger, O., Tunyasuvunakool, K., Bates, R., Žídek, A., Potapenko, A., Bridgland, A., Meyer, C., Kohl, S. A. A., Ballard, A. J., Cowie, A., Romera-Paredes, B., Nikolov, S., Jain, R., Adler, J., Back, T., Petersen, S., Reiman, D., Clancy, E., Zielinski, M., Steinegger, M., Pacholska, M., Berghammer, T., Bodenstein, S., Silver, D., Vinyals, O., Senior, A. W., Kavukcuoglu, K., Kohli, P., and Hassabis, D. (2021) Highly accurate protein structure prediction with AlphaFold. *Nat.* **596**, 583–589
12. Mirdita, M., Schütze, K., Moriwaki, Y., Heo, L., Ovchinnikov, S., and Steinegger, M. (2022) ColabFold: making protein folding accessible to all. *Nat. Methods*. **19**, 679–682

13. Ashkenazy, H., Erez, E., Martz, E., Pupko, T., and Ben-Tal, N. (2010) ConSurf 2010: calculating evolutionary conservation in sequence and structure of proteins and nucleic acids. *Nucleic Acids Res.* **38**, W529–W533
14. Holm, L., and Rosenstrom, P. (2010) Dali server: conservation mapping in 3D. *Nucleic Acids Res.* **38**, W545–W549
15. Kocharova, N. A., Mieszała, M. S., Zatonsky, G. V., Staniszewska, M., Shashkov, A. S., Gamian, A., and Knirel, Y. A. (2004) Structure of the O-polysaccharide of *Citrobacter youngae* O1 containing an α-D-ribofuranosyl group. *Carbohydr. Res.* **339**, 321–325
16. Mieszała, M., Lipiński, T., Kocharova, N. A., Zatonsky, G. V., Katzenellenbogen, E., Shashkov, A. S., Gamian, A., and Knirel, Y. A. (2003) The identity of the O-specific polysaccharide structure of *Citrobacter* strains from serogroups O2, O20 and O25 and immunochemical characterisation of *C. youngae* PCM 1507 (O2a,1b) and related strains. *FEMS Microbiol. Immunol.* **36**, 71–76
17. Thoden, J. B., Frey, P. A., and Holden, H. M. (1996) Molecular structure of the NADH/UDP-glucose abortive complex of UDP-galactose 4-epimerase from *Escherichia coli*: implications for the catalytic mechanism. *Biochemistry*. **35**, 5137–5144
18. Kokoulin, M. S., Romanenko, L. A., Kuzmich, A. S., and Chernikov, O. (2021) Structure of the cell-wall-associated polysaccharides from the deep-Sea marine bacterium *Devosia submarina* KMM 9415T. *Marine Drugs 2021, Vol. 19, Page 665*. **19**, 665
19. Neelamegham, S., Aoki-Kinoshita, K., Bolton, E., Frank, M., Lisacek, F., Lütteke, T., O'Boyle, N., Packer, N. H., Stanley, P., Toukach, P., Varki, A., Woods, R. J., Group, T. S. D., Darvill, A., Dell, A., Henrissat, B., Bertozzi, C., Hart, G., Narimatsu, H., Freeze, H., Yamada, I., Paulson, J., Prestegard, J., Marth, J., Vliegenthart, J. F. G., Etzler, M., Aebi, M., Kanehisa, M., Taniguchi, N., Edwards, N., Rudd, P., Seeberger, P., Mazumder, R., Ranzinger, R., Cummings, R., Schnaar, R., Perez, S., Kornfeld, S., Kinoshita, T., York, W., and Knirel, Y. (2019) Updates to the Symbol Nomenclature for Glycans guidelines. *Glycobiology*. **29**, 620–624
20. Bethesda Research Laboratories (1986) BRL pUC host: *E. coli* DH5αTM competent cells. *Focus (Madison)*. **8**, 9–12
21. Guzman, L. M., Belin, D., Carson, M. J., and Beckwith, J. (1995) Tight regulation, modulation, and high-level expression by vectors containing the arabinose PBAD promoter. *J. Bacteriol.* **177**, 4121–4130
22. Datsenko, K. A., and Wanner, B. L. (2000) One-step inactivation of chromosomal genes in *Escherichia coli* K-12 using PCR products. *Proc. Natl. Acad. Sci.* **97**, 6640–6645
23. Snyder, S. A., Treitler, D. S., and Brucks, A. P. (2010) Simple reagents for direct halonium-induced polyene cyclizations. *J Am Chem Soc*. **132**, 14303–14314
24. Crich, D., Banerjee, A., Li, W., and Yao, Q. (2005) Improved synthesis of 1-benzenesulfinyl piperidine and analogs for the activation of thioglycosides in conjunction with trifluoromethanesulfonic anhydride. *J Carbohydr Chem*. **24**, 415–424

25. Walvoort, M. T. C., De Witte, W., Van Dijk, J., Dinkelaar, J., Lodder, G., Overkleeft, H. S., Codée, J. D. C., and Van Der Marel, G. A. (2011) Mannopyranosyl uronic Acid donor reactivity. *Org Lett*. **13**, 4360–4363
26. Sun, B., Srinivasan, B., and Huang, X. (2008) Pre-Activation-Based One-Pot Synthesis of an α-(2,3)-Sialylated Core-Fucosylated Complex Type Bi-Antennary N-Glycan Dodecasaccharide. *Chemistry – A European Journal*. **14**, 7072–7081
27. Tilekar, J. N., and Lowary, T. L. (2004) An efficient route to thioglycosides with the 2,3-anhydro-D-ribo stereochemistry. *Carbohydr Res*. **339**, 2895–2899
